# Alexidine, identified as a Z-DNA inducer by the NanoZ screening platform, acts as a transcriptional regulator

**DOI:** 10.1093/nar/gkag281

**Published:** 2026-04-08

**Authors:** Vinod Kumar Subramani, Shrute Kannappan, Shiyu Wang, Yan Xu, Subramaniyam Ravichandran, Boi Hoa San, Jung Heon Lee, Kyeong Kyu Kim

**Affiliations:** Department of Precision Medicine, Institute for Antimicrobial Resistance Research and Therapeutics, Sungkyunkwan University School of Medicine, Suwon 16419, Korea; Department of Precision Medicine, Institute for Antimicrobial Resistance Research and Therapeutics, Sungkyunkwan University School of Medicine, Suwon 16419, Korea; Research Center for Advanced Materials Technology, Core Research Institute, Suwon 16419, Korea; Division of Chemistry, Department of Medical Sciences, Faculty of Medicine, University of Miyazaki, 5200 Kihara, Kiyotake, Miyazaki 889-1692, Japan; Division of Chemistry, Department of Medical Sciences, Faculty of Medicine, University of Miyazaki, 5200 Kihara, Kiyotake, Miyazaki 889-1692, Japan; Department of Precision Medicine, Institute for Antimicrobial Resistance Research and Therapeutics, Sungkyunkwan University School of Medicine, Suwon 16419, Korea; Department of Precision Medicine, Institute for Antimicrobial Resistance Research and Therapeutics, Sungkyunkwan University School of Medicine, Suwon 16419, Korea; Research Center for Advanced Materials Technology, Core Research Institute, Suwon 16419, Korea; Department of Advanced Material Science and Engineering, Sungkyunkwan University, Suwon 16419, Korea; Department of Precision Medicine, Institute for Antimicrobial Resistance Research and Therapeutics, Sungkyunkwan University School of Medicine, Suwon 16419, Korea

## Abstract

Z-DNA is a left-handed DNA helix implicated in gene regulation, genome stability, and immune responses, yet effective small-molecule modulators in cells remain scarce. To address this gap, we developed NanoZ, a two-step screening platform designed to identify Z-DNA-inducing molecules. The first step, a DNA condensation assay, detects ligand-induced DNA condensation using gold nanoparticles functionalized with Z-DNA-forming sequences, providing a rapid optical readout through localized surface plasmon resonance. The second step, a biophysical validation pipeline, employs circular dichroism, 2-aminopurine fluorescence, and NMR spectroscopy to confirm Z-DNA formation. Using this workflow, we screened 2000 compounds from the Natural Product and Prestwick Chemical Libraries, identifying 18 positive hits in the condensation assay and selecting Alexidine dihydrochloride as a Z-DNA inducer. Alexidine efficiently promoted the B-to-Z transition and DNA condensation *in vitro*, and markedly increased Z-DNA formation in cells, as confirmed by immunofluorescence and ChIP-seq. Genome-wide analysis revealed that Alexidine-induced Z-DNA localized to transcriptionally active, purine-pyrimidine repeat-rich loci, leading to gene repression. Molecular dynamics simulations showed that Alexidine bridges adjacent Z-DNA helices, facilitating condensation. Collectively, our findings highlight Alexidine as the first small-molecule Z-DNA inducer that modulates transcription in cells and establish NanoZ as a versatile platform for discovering Z-DNA modulators.

## Graphical Abstract

**Graphical Abstract:**
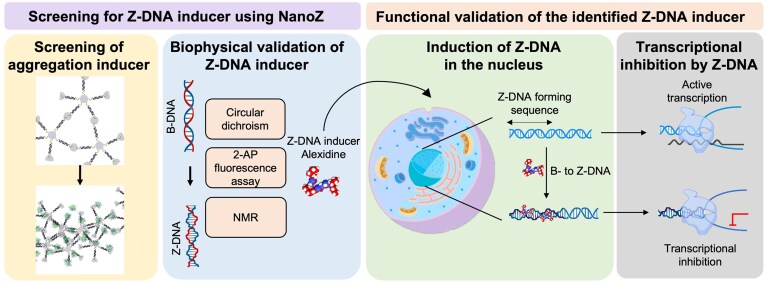

## Introduction

DNA in the human genome can adopt several non-canonical conformations in addition to the predominant B-DNA with a right-handed helical conformation known as Watson-Crick DNA [1]. An alternating purine-pyrimidine dinucleotide repeat sequence can undergo helical transition into left-handed Z-DNA [2], which is one of the most important non-canonical conformations. Cellular events, such as negative supercoiling and binding of Z-DNA-binding proteins, such as ADAR1, DAI, and PKZ, can induce the B-to-Z DNA transition [3–10]. During this transition, base extrusion at the junction is necessary [11–13]. Z-DNA is known to control many key cellular events such as transcriptional gene regulation [14–16], differentiation [17], genetic instability [18–20], and immune responses [21]. Consequently, Z-DNA has been implicated in several diseases such as Cancer, Aicardi-Goutières syndrome, and Systemic Lupus Erythematosus [22, 23].

However, there are few reports on Z-DNA-targeted intervention strategies that affect changes in gene regulation, genetic instability, immune response, or disease manifestation, largely due to the limited number and low efficacy of available Z-DNA modulators. For example, Z-DNA-binding proteins and antibodies are well characterized *in vitro* but are ineffective *in vivo* or in live cells because of their size and delivery methods. Although Z-DNA-modulating chemicals are useful, they are generally unsuitable for cell studies because of their low membrane permeability and poor stability under physiological conditions [24]. Therefore, novel Z-DNA modulators that are active under physiological conditions need to be identified for understanding and investigating the cellular functions of Z-DNA. Current tools for identifying Z-DNA modulators detect conformational changes in DNA, either B-to-Z or Z-to-B, by applying various physicochemical or biochemical methods [25]. However, these strategies are labor-intensive, expensive, and sometimes require expertise, making them unsuitable for high-throughput testing of thousands of compounds in large chemical libraries. Therefore, an efficient screening method for identifying potent Z-DNA modulators is required.

To accommodate the minuscule volume within the cellular environment, double-stranded DNA (dsDNA) undergoes highly condensed organization at various levels of genome organization, such as the nucleoid in prokaryotes [26], chromatin and chromosomes in eukaryotes [27], and within viral capsids [28, 29]. This raises a fundamental question regarding the mechanism by which highly negatively charged dsDNA molecules can compact together, overcoming the unfavorable electrostatic repulsive interactions of phosphates. Several factors have been reported to drive DNA condensation. For example, electrostatic interactions involve neutralization of negatively charged phosphates on DNA by cations or ligands, including multivalent cations, polyamines, or proteins. Various experimental studies have demonstrated that DNA condensation can be achieved using positively charged small molecules such as spermine [30] and hexammine [31], which compensate for the repulsion between negatively charged DNA backbones. In addition, macromolecular crowding, which drives DNA to occupy a smaller volume through excluded volume effects and restructuring of nearby water molecules, helps DNA pack tightly, has also been proposed to contribute to molecular condensation. It is reasoned that a combinatorial effect of one or more of these cation-driven interactions drives DNA condensation [32–34]. Since some positively charged molecules are known to enhance the stabilization of certain secondary structures of DNA, such as Z-DNA and G-quadruplexes [35–38], these non-canonical DNA structures may also be involved in DNA condensation. Thus, the condensation of Z-DNA is mediated by the binding of cationic molecules [39–51]. Interestingly, it has been proposed that an intermediate DNA conformation called Z*-DNA, which exhibits properties similar to those of Z-DNA, is formed during condensation [52–54], suggesting the involvement of various DNA structures in DNA condensation. Furthermore, there is growing evidence to support the idea that Z-DNA conformations are involved in the higher-order structural organization of the genome [52, 55–59]. Therefore, molecules that induce DNA condensation may also possess the property to stabilize Z-DNA or vice versa.

In this study, we hypothesized that small-molecule Z-DNA inducers could induce DNA condensation. After confirming this hypothesis using several known Z-DNA inducers, we postulated that certain DNA condensation inducers may also function as Z-DNA inducers. To investigate this notion, we developed a novel screening platform, NanoZ (Supplementary Fig. S1), which employs DNA-conjugated gold nanoparticles (DCGNPs) and detects ligand-induced condensation through bathochromic shifts in localized surface plasmon resonance (LSPR). Using NanoZ, we screened chemical libraries to identify candidate molecules that trigger DNA condensation comparable to known Z-DNA inducers. These candidates were subsequently validated through multiple biophysical methods to confirm their ability to promote Z-DNA formation both *in vitro* and in cells. Using this platform, we successfully identified Alexidine dihydrochloride as a potent Z-DNA inducer and further analyzed its binding mode and condensation mechanism via molecular dynamics (MD) simulations. Moreover, by applying Chromatin immunoprecipitation followed by sequencing (ChIP-seq) and gene expression analyses with Alexidine, we uncovered new cellular functions of Z-DNA, demonstrating that chemically induced Z-DNA formation can inhibit gene expression in specific genomic contexts. This provides the first direct evidence that a small-molecule Z-DNA inducer can modulate transcription in cells. Collectively, this work establishes the NanoZ platform as a powerful and necessary approach for discovering Z-DNA modulators and validating their biological relevance in cells.

## Materials and methods

### Protein preparation

hZα_ADAR1_: The human Zα_ADAR1_ (residues 140–202; Genbank ID NP_001102.2) was expressed and purified as described in [60]. Briefly, the coding sequence was cloned into a pET28a(+) bacterial expression plasmid. Subsequently, the protein was expressed in *Escherichia coli* BL21 DE3 strain with a fused His-tag at the N-terminal end and subsequently purified. The expressed cells were harvested and lysed by sonication. The released protein was first affinity-purified on a HiTrap metal-chelating column (GE Healthcare, NJ, USA), after which the His-tag was enzymatically cleaved using thrombin (Merck Millipore, USA). Subsequently, the tag-free protein was purified by ion exchange chromatography using a HiTrap SP column (GE Healthcare, NJ, USA). The resultant pure protein was collected, and its concentration was measured spectroscopically using extinction coefficient of 6990 M^−1^cm^−1^ at 280 nm. Denatured Zα protein was prepared by boiling Zα_ADAR1_ protein for 5 min, followed by cooling on ice. Similarly, goldfish Zα PKZ (caZα_PKZ_, 1–75 residues, GenBank accession number AAP49830.1) was purified as reported previously [61].

Histone: Total histones were prepared from HeLa cells grown in culture using an acid extraction protocol [62]. Total histone concentration was determined by Bradford assay (Sigma–Aldrich, USA) using a standard curve against bovine serum albumin (BSA).

Albumin: BSA was purchased from Sigma–Aldrich, USA (Cat. A9418), and a stock solution of 1 mg/ml was prepared in autoclaved ultrapure water.

### B-to-Z DNA transition activity by circular dichroism spectroscopy

Circular dichroism (CD) spectroscopy was used to check B-to-Z DNA transition activity in the presence of Zα [63]. All experiments were conducted at 25°C in a JASCO J-810 CD spectrometer (MD, USA) between 230 and 320 nm at 1 nm intervals. The average of three measurements was recorded. (CG)_6_, double-stranded (CG)_6_ oligonucleotide in Table 1, was prepared by incubating the self-complementary ODN at room temperature (RT), and its concentration was measured using a spectrophotometer (Nanodrop, Thermo Scientific, MA, USA). For B-to-Z transition activity testing, 10 or 15 µM of (CG)_6_ was treated with four molar excess (40 or 60 µM of purified Zα, respectively) concentration determined from crystal structure information [64]. For testing of small-molecule compounds, the samples were prepared similar to that of Zα with the indicated molar excess concentration of small molecules as described in the respective figure caption. To evaluate the equilibrium binding, the wavelength was fixed at 255 nm and ellipticity changes were recorded over 1 h to obtain rate constants using a one-phase association model. From the endpoint equilibrium ellipticities of the B-to-Z transition, the fraction of Z-DNA was calculated as:


\begin{eqnarray*}
\textit{Fraction}\ Z\ = \ \ \frac{{\left( {\theta {\mathrm{\ }} - {\mathrm{\ }}{{\theta }_B}} \right)}}{{\left( {{{\theta }_Z} - {{\theta }_B}} \right)}}
\end{eqnarray*}

**Table 1.** List of oligodeoxynucleotides used in this study

Name	Sequence	Size of dsDNA (base pair)	Size of spacer(base)
(CG)_6_	5′-CG CG CG CG CG CG-3′3′-GC GC GC GC GC GC-5′	12	NA
(TA)_6_	5′-TA TA TA TA TA TA-3′3′-AT AT AT AT AT AT-5′	12	NA
NZ-1	5′-^/5ThioMC6-D^/AAAA(CG)_6_–3′	12	4
NZ-2	5′-^/5ThioMC6-D^/AAAAAAAAAA(CG)_6_–3′	12	10
NZ-3	5′-^/5ThioMC6-D^/AAAA(CG)_24_–3′	48	4
NZ-4	5′-^/5ThioMC6-D^/AAAA(CG)_48_–3′	96	4
ZB-2ap0	5′-CGCGCGCGCGCG(2AP)TAAACCACTCGG-3′3′-GCGCGCGCGCGC(T)ATTTGGTGAGCC-5′	25	NA
ZB-2ap4	5′-CGCGCGCGCGCGATAA(2AP)CCACTCGG-3′3′-GCGCGCGCGCGCTATT(T)GGTGAGCC-5′	25	NA
FG-op	5′-CG C^F^GCG-3′3′-GC^F^G CGC-5′	6	NA
FG-sm	5′-C^F^GCAC^F^GCG-3′3′-G C GTG CGC-5′	8	NA

2AP = 2-aminopurine; ^F^G = 8-trifluoromethyl-2′-deoxyguanosine

where θ_B _= ellipticity of pure B-DNA; θ_Z _= ellipticity of pure Z-DNA induced by the Zα protein at maximum concentration (10×). The resulting Fraction Z values were plotted as a function of Z-inducer concentration and fitted by non-linear regression using a one-site specific binding model to determine the *K*_D_.

### DNA condensation assay

DNA condensation assay was performed to assess the extent of spermine-induced condensation as reported [65]. Briefly, an inducer such as Z⍺ protein, spermine (Sigma–Aldrich, USA; Cat. S4513), or Alexidine (Sigma–Aldrich, USA Cat. A8986) was incubated with (CG)_6_ or salmon sperm DNA. Z⍺ protein was prepared in 5 mM HEPES (pH 7.5; Thermo Scientific, MA, USA) and 10 mM NaCl (Glentham Life Sciences, UK), and salmon sperm DNA was prepared in a buffer containing 20 mM Tris–HCl (Glentham Life Sciences, UK), 1 mM EDTA (Glentham Life Sciences, UK), 50 mM NaCl (Glentham Life Sciences, UK). After incubation of inducers with DNA for 1 h at RT, the samples were centrifuged at 21 000 × *g* for 5 min. The concentration of the DNA in the supernatant was measured from 1 µl of supernatant carefully taken, using a spectrophotometer (Nanodrop, Thermo Scientific, MA, USA). The data obtained are expressed as the fold change in DNA absorbance (*A*_260_) in the presence of the small molecule and plotted for analysis. For all assays, appropriate concentrations of nucleic acids and inducers (Z⍺, spermine, alexidine) were used according to the experimental design.

### Colloidal gold nanoparticles

The 20 nm gold nanoparticle (GNP) used in this study was purchased as a ready-to-use colloid from BBI Solutions (Cardiff, UK).

### DNA-conjugated gold nanoparticle probe preparation

Oligodeoxynucleotides (ODNs) for DCGNP synthesis were purchased as 5′ thiol-modified variants from Integrated DNA Technologies (IDT, Singapore). They were dissolved in sterile, autoclaved ultrapure water (DW), and their concentrations were measured using a spectrophotometer (NanoDrop, Thermo Scientific, MA, USA). All ODNs used in DCGNP preparation are self-complementary and readily form double helices at RT (Table 1). Functionalization of GNP with DNA was performed by slightly modifying the protocol described by Taton *et al.* [66]. The required amount of ODN was calculated as described in the protocol [66] based on the size of GNP (0.3 µM of NZ-3 ODN was used for conjugating with 1.16 nM of 20 nm GNP).

Equation: mol conjugated ODN = A_n_ × *c*_n_ × *D*_o_ × *V*

where *A*_n_ is the surface area of the nanoparticle given by 4π*r*^2^ (*r* is the radius of the nanoparticles), *c*_n_ is the concentration of the nanoparticle solution (in nanoparticles per liter), *D*_o_ is the oligonucleotide density on each particle (∼35 pmol oligonucleotide per cm^2^), and *V* is the volume of nanoparticle solution (in L).

As recommended by Taton [66], a 1.5-fold molar excess of thiolated ODN relative to the calculated requirement was used to ensure saturation of the available gold surface. In practice, 0.45 µM NZ-3 ODN (approximately 8 µg) in a 50 µl volume was reduced by adding 10 µl of 1 N dithiothreitol (DTT; Thermo Scientific, MA, USA) and incubating at room temperature for 15 min. The reduced ODNs were purified from excess DTT by three sequential extractions with anhydrous ethyl acetate (Sigma–Aldrich, USA), and the aqueous phase containing the deprotected thiol-ODNs was immediately combined with the colloidal GNP suspension. The mixture was incubated overnight at room temperature with orbital shaking at 300 rpm to allow formation of Au–S bonds and high-density ODN monolayers .​

After conjugation, excess free thiol-ODN was removed by repeated centrifugation and resuspension in ultrapure water, analogous to the purification schemes used in Taton [66] and in Wang *et al.* [67]. For 20 nm GNPs, DCGNPs were pelleted at 10 000 × *g* for 20 min in each cycle (conditions consistent with Taton’s size-dependent recommendations), and the supernatant containing unbound ODN was discarded. The final DCGNP pellet was resuspended in 500 µl of ultrapure water and stored at 4°C in the dark until use. Immediately before experiments, DCGNP suspensions were subjected to brief low-frequency sonication (30 Hz, 3 s) to redisperse reversible aggregates and obtain a reproducible dispersion state for all assays.

### Ultraviolet–visible (UV–Vis) spectroscopy

A total of 50 µl sample reactions were prepared in duplicates by mixing 40 µl synthesized DCGNP probe (0.8 µg DNA), with either 10 µl buffer, 10 µg protein (28 µM), or 50 µM (1–5 µg) chemical or small molecule in buffer containing 5 mM HEPES (pH 7.5; Thermo Scientific, MA, USA) and 10 mM NaCl (Glentham Life Sciences, UK) within 96-well microplates capable of UV–Visible extinction spectra measurement (sum of both absorbance and scattering phenomena). UV–Vis extinction measurements were performed using a Synergy NEO multiplate reader machine (BioTek Instruments Inc., VT, USA).

### Dynamic light scattering and microscopic observation of DNA condensates

The hydrodynamic diameter of the reaction mixture was measured using DynaPro (Wyatt Technologies, CA, USA). All measurements were performed at 25°C. Inverted light microscopy images and movies were recorded using a standard inverted light microscope (Olympus, TKY, Japan) with a ×10 objective equipped with a digital camera.

### Chemical screening

The chemicals in the Natural Product Library (Microsource Discovery System, CT, USA) and Prestwick Chemical Library (Prestwick Chemical, France) were screened at a final concentration of 50 µM using 96-well microplates with the prepared DCGNP probe. Their UV–Vis extinction profiles were scanned to identify the LSPR maximum shift toward the red region. Test chemicals that showed a red-shifted LSPR maximum comparable to that in the presence of Zα were considered positive hits. These hits were further tested by CD spectroscopy as a confirmatory test in the presence of Z-DNA-forming (CG)_6_ ODN. Based on the resultant Z-DNA-specific CD profile, the test chemical was categorized as a candidate Z-DNA inducer and further validated.

### 2-Aminopurine (2AP) base extrusion fluorescence assay (2AP–BEFA)

2AP–BEFA was performed as described by Subramani *et al.* [68]. Briefly, two separate dsODNs with 2AP at the BZ junction site—zero and four were prepared by annealing the complementary ZB-2ap0 and ZB-2ap4 ODNs, respectively (Table 1), and the BZ junction was readily induced in the presence of purified Zα protein in both the probes. However, because the 2AP at the junction site position “0” is present only in the ZB-2ap0 probe, the ZB-2ap0 probe can give junction formation signal, while in the ZB-2ap4 probe, as the 2AP is at position “4,” this probe will not give the 2AP signal as it forms part of the region in the B-DNA conformation. Fluorescence from 2AP exposed at the BZ junction was measured at 25°C in a buffer containing 5 mM HEPES (pH 7.5; Thermo Scientific, MA, USA) and 10 mM NaCl (Glentham Life Sciences, UK), using a Synergy NEO multiplate reader machine (BioTek Instruments Inc., VT, USA). Excitation and emission wavelengths were 320 and 380 nm, respectively.

### Nuclear magnetic resonance (^19^F-NMR) Spectroscopy

Prior to the ^19^F-NMR experiments, CD experiments were performed on a JASCO model J-820 (MD, USA) CD spectrophotometer. Duplex DNA FG-op or FG-sm (Table 1) was prepared at 10 µM in 10 mM NaCl and 1 mM NaPO_4_ (pH 7.0) at 10°C. For the study of spermine-induced Z-DNA formation assay, the DNA duplex was prepared at 10 µM in 10 mM NaCl and 1 mM NaPO_4_ buffer (pH 7.0). Spermine was added into the DNA solution and kept at room temperature for 2 h before each measurement. In a similar way, Alexidine was added into the DNA solution for experiments.

The ^19^F-NMR experiments were performed as described in an earlier report [69]. Briefly, DNA duplex samples were dissolved in 150 µl of a designed solution containing 1 mM NaPO_4_ buffer (pH 7.0) and 10% D_2_O in 10 mM NaCl. The ^19^F-NMR spectrum was measured on a Bruker AVANCE 500 MHz spectrometer at a frequency of 376.05 MHz and referenced to the internal standard CF3COOH (–75.66 ppm). The experimental parameters are recorded as follows: spectral width 89.3 kHz, ^19^F excitation pulse 15.0 µs, relaxation delay 1.5 s, acquisition time 0.73 s, scan numbers 1024–4096, and line width 3. For the small-molecule-induced Z-form DNA formation experiment, the DNA duplex sample was prepared at 0.1 mM concentration in the presence of 10 mM NaCl and 1 mM NaPO_4_ buffer (pH 7.0). Spermine or Alexidine was added into the DNA solution and kept at room temperature for 2 h before each measurement.

### Quantitative assay of B- and Z-form DNA by ^19^F-NMR spectroscopy

The ^19^F signals at –61.08 and –61.51 ppm were assigned to the Z-form and B-form DNA, respectively, when analysing the small-molecule-induced B-to-Z transition. For the quantitative analysis of the B- and Z-form DNA, the ^19^F signals were calibrated, and the percentage of Z-DNA was calculated using the following formula:


\begin{eqnarray*}
&&{{\mathrm{ Percentage\ of\ Z}}} \mathrm{-} {\mathrm{DNA}}\left( {\mathrm{\% }} \right)\\&&= \tfrac{{^{{\mathrm{19}}}{\mathrm{F\ signal\,}}{{{\left( { - 61.08{\mathrm{\,ppm}},{\mathrm{Z}} \mathrm{-} {\mathrm{DNA}}} \right)}}_{{\mathrm{Calibration}}}}}}{{^{{\mathrm{19}}}{\mathrm{F\ signal\,}}{{{\left[ {\left( { - 61.08{\mathrm{\,ppm}},{\mathrm{Z}} \mathrm{-} {\mathrm{DNA}}} \right) + \left( { - 61.51{\mathrm{ppm}},{\mathrm{B}} \mathrm{-} {\mathrm{DNA}}} \right)} \right]}}_{{\mathrm{Calibration}}}}}}\times \mathrm{ 100{{\% }}}
\end{eqnarray*}

### Cell culture

Mouse embryonic fibroblasts (MEFs) were isolated from pregnant mice on embryonic day 13.5 (E13.5) from CellapeuticsBio, KR. Primary MEFs were maintained in DMEM (Welgene, KR) supplemented with 10% FBS (Gibco, USA) and 1% penicillin–streptomycin (Welgene, KR). The cells were cultured at 37°C in a 5% CO_2_ incubator.

### Immunofluorescence microscopy

MEFs were seeded in a confocal dish (SPL Life Sciences, KR) at a density of 0.37 × 10^4^ cells/cm^2^ and allowed to adhere for 12 h. Cells were treated with spermine (5 μM, Sigma–Aldrich, USA; Cat. S4513), Alexidine (1 μM, Sigma–Aldrich, USA; Cat. A8986), or CBL0137 (5 μM, Cayman Chemical, MI, USA; Cat. 19 110) for 24 h. Following treatment, cells were washed thrice with 1 × phosphate-buffered saline (PBS, Welgene, KR) and incubated for 10 min in 4% (w/v) formaldehyde (Sigma–Aldrich, USA) in PBS at RT for fixation. The cells were washed with PBS and permeabilized with 0.1% (v/v) Triton-X-100 (USB Corporation, OH, USA) in PBS for 10 min at RT. After three PBS washes, the permeabilized cells were incubated in a blocking solution containing 1% BSA (Sigma–Aldrich, USA), 22.52 mg/ml glycine (Affymetrix, CA, USA), and 0.1% Tween 20 (Affymetrix, CA, USA) in PBS for 1 h at RT. The cells were subsequently incubated overnight at 4°C in primary rabbit anti-Z-DNA (Z22) antibody (Absolute antibody, UK; Cat. Ab00783-23.0) diluted 1:10 000 [20] in a dilution buffer containing 1% BSA and 0.1% Tween 20 in PBS. Further, the cells were washed thrice with PBST (0.1% Tween 20 in PBS) and incubated with goat anti-rabbit IgG secondary antibody Alexa Fluor 488 (Thermo Scientific, MA, USA; Cat. A-11034) at a dilution of 1:2000 [20] in dilution buffer for 1 h at RT. The cells were washed thrice with PBST and incubated in 1 μg/ml of DAPI (Sigma–Aldrich, USA) in PBS for 5 min at RT to stain the nuclei. Samples were visualized using a confocal microscope (LSM710, ZEISS, Germany). All confocal images were analyzed using Fiji image processor [70]. The relative fluorescence intensities for the different conditions were calculated and plotted as the fold change in the difference between the area-integrated intensity and the background mean gray value.

### Cytotoxicity assay

Cell viability was assessed using a WST-8-based colorimetric assay according to the manufacturer’s instructions (Abcam, UK). Briefly, MEFs were seeded in 96-well plates and treated with the indicated concentrations of Z-DNA inducer for 24 h. WST-8 reagent was then added to each well and incubated for 2 h at 37°C, after which absorbance was measured at 450 nm using the Synergy NEO multiplate reader machine (BioTek Instruments Inc., VT, USA). Background-corrected absorbance values were normalized to untreated controls and expressed as percentage viability.

### ChIP-seq

MEFs treated with or without Alexidine were subjected to ChIP using the ChIP-IT Express Enzymatic kit (Active Motif) based on the manufacturer’s instructions. Briefly, the cells were fixed for 10 min with 1% (w/v) formaldehyde in DMEM. Following this, the crosslinked DNA was sheared with an enzymatic shearing cocktail (provided in the kit) for 10 min to aid digestion of chromatin, yielding DNA of about 200–500 bp in length. Further, 10 μg of the sheared DNA was immunoprecipitated with 2 μg of anti-Z-DNA (Z22) antibody overnight at 4°C. Library preparation, paired-end DNA sequencing, and preliminary raw data pre-processing were performed by ebiogen (Korea). Briefly, ChIP DNA libraries were prepared using the NEBNext^®^ Ultra^™^ DNA Library Prep Kit for Illumina (New England Biolabs, UK) according to the manufacturer’s protocol. Briefly, ChIP-enriched DNA fragments were end-repaired, adaptor-ligated, and PCR-amplified using indexed primers for multiplexing. Libraries were purified using magnetic beads, and fragment size distribution was assessed using an Agilent 2100 Bioanalyzer (Agilent Technologies, Netherlands). Paired-end 100 bp sequencing was performed on an Illumina NovaSeq 6000 platform. Raw sequencing reads were processed using fastp (v0.23.1) to remove adapter sequences and low-quality reads [71]. Clean reads were aligned to the mouse reference genome (mm10) using Bowtie2 (v2.3.4.3) [72]. PCR duplicates were removed using SAMtools (v1.17), and ENCODE blacklist regions were excluded from downstream analyses [73, 74]. Peak calling was performed using MACS2 with default parameters. Differential peak analysis was conducted using the Python package conorm (v1.2.0) [75]. Only high-confidence peaks passing statistical thresholds were retained for further analysis. Quality control metrics, including read filtering, mapping rates, duplicate removal, and blacklist filtering, were evaluated in accordance with ENCODE guidelines. Processed alignment files and peak files have been deposited in GEO under accession number GSE312055, and UCSC genome browser session files are provided in the data availability section.

### Gene expression analysis by qRT-PCR

Total RNA from MEFs was extracted using the RNeasy Mini kit (QIAGEN, Germany). RNA purity and integrity were assessed by measuring the A260/280 and A260/230 ratios with a Nanodrop spectrophotometer, and samples with ratios between 1.9 and 2.1 were used for downstream analysis. A total of 1 μg of total RNA was reverse transcribed into complementary DNA using HiScript III RT SuperMix (Vazyme, China) following the manufacturer’s protocol, which also included a genomic DNA removal step. Further, qRT-PCR was performed using the CFX Connect machine (Bio-Rad, CA, USA) with the Taq Pro Universal SYBR qPCR Master Mix (Vazyme, China) according to the manufacturer’s instructions. Each reaction contained 20 ng of cDNA template and was run in technical triplicate with three independent biological replicates. The primers used for qRT-PCR are listed in Supplementary Table S1. Primer specificity was confirmed by melt curve analysis, demonstrating a single amplification peak. No-template controls (NTCs) were included for each primer pair to exclude contamination or primer-dimer formation. Primer efficiencies were determined using five-point serial dilutions (50–0.16 ng) of pooled cDNA. Standard curves were generated by plotting Ct values against log10-transformed template input. Efficiencies were calculated using the equation:


\begin{eqnarray*}
E = \left( {{{{10}}^{\left( {\frac{{ - 1}}{{\textit{slope}}}} \right)}} - 1} \right) \times \ 100
\end{eqnarray*}

and ranged between 90% and 110% with *R*^2 ^≥ 0.98. Reference gene stability was evaluated by comparing the Ct variation of GAPDH and L32 across experimental groups. GAPDH demonstrated minimal variation (<0.1 Ct) and low intra-group variability and was therefore selected as the normalization control. The relative gene expression of the tested genes was normalized to GAPDH using the 2^−ΔΔCT^ method.

### Structural analyses and MD simulation

#### DNA models

The initial Z-DNA model of one turn – 12 bp (CG)_6_ and the B-DNA model of one turn – 10 bp (CG)_5_ were built using Web 3DNA 2.0 (http://web.x3dna.org/) [76]. One/three identical DNA duplexes were placed within a cubic water box of dimensions of either 4.46 × 10.0 × 6.0 nm^3^ (Z-DNA) or 3.4 × 10.0 × 6.0 nm^3^ (B-DNA) with periodic boundary conditions imposed in all directions. The helices were aligned along the *x*-axis, with the end nucleotides of each strand forming a long bond across the box length. Therefore, effectively mimicking infinite DNA along the *x*-axis, the DNA was free to rotate about the *x*-axis and translate in all directions. To build an array of 64 Z-DNA, a single infinitely long Z-DNA duplex within a simulation box of 4.46 × 2.8 × 2.8 nm^3^ was replicated 64 times to yield a system that measures 4.46 × 22.4 × 22.4 nm^3^. All-atom MD simulations were performed in salt-free systems to eliminate the effects of salt-induced inter-DNA attractions [59]. To maintain the chemical neutrality of the system, a suitable number of Mg^2+^ counterions were added to the water box.

#### All-atom MD protocol

All-atom MD simulations were performed using the GROMACS 2021 package [77]. All simulations were performed using AMBER ff99-derived force fields: bsc0 for DNA [78], Joung and Cheatham for ions [79], GAFF for ligands, and TIP3P for water [80]. Custom-pair-specific van der Waals parameter (CUFIX) corrections were used for various non-bonded interactions [81, 82]. The SHAKE constraints were applied to all bonds involving hydrogen atoms. Long-range electrostatic interactions were computed using the Particle Mesh Ewald (PME) method with a Fourier grid spacing of 0.16, and a cutoff distance of 12 Å was considered for the van der Waals interactions. Constant pressure and temperature (300 K, 1 atm) were maintained in the system, with the non-bonded pair list updated every 10 fs and an integration time step of 2 fs. Preliminary studies were performed by adding five ligands (spermine/Alexidine) at random positions within a simulation box consisting of three duplex infinite Z-DNA/B-DNA. In the system with 64 duplexes, for the low ligand concentration, 40 ligands (spermine or Alexidine) were added to the simulation box at random positions. For the maximum ligand concentration studies, 322 Alexidines or 500 spermines were added to the simulation box. Upon addition of the ligands, the system was equilibrated by pressure and temperature coupling for 500 ps. Finally, a 50 ns MD simulation was performed. The first 10 ns of the trajectory in all simulations were discarded from further analysis. Hydrogen-bond maps, inter-DNA distances, and ligand density maps were evaluated using appropriate GROMACS-associated tools.

#### All-atom potential of mean force calculation

The infinite Z-DNA system previously mentioned was used for potential of mean force (PMF) calculations using umbrella sampling, except that it was restricted to two Z-DNA duplexes. The system was equilibrated using 100 mM MgCl_2_ for 50 ns. The last snapshot from the equilibration run was used as the starting conformation for the umbrella sampling. The inter-Z-DNA distance in the *y*–*z* plane was constrained by a harmonic spring. The reaction coordinate along the *y*-axis varied from 1.6 to 3.5 nm in increments of 0.1 nm with a force constant of 1000 kJ mol^−1^ nm^−2^. At each distance, a 50 ns umbrella sampling simulation was performed. The last 10 ns of the trajectory for each inter-Z-DNA distance were used for weighted histogram analysis (WHAM) to determine the free energy profile.

#### Binding energy calculations

The binding energy of the ligand was estimated by the single trajectory protocol (STP) with the Molecular Mechanics – Poisson Boltzmann Surface Area (MM-PBSA) method with the gmx_MMPBSA module [83]. The MD snapshots extracted from the last 10 ns of the 50 ns MD simulation (single/three duplex system), at an interval of 500 ps, were used to calculate the enthalpy of binding of the ligand to DNA. As the charge of the system was very high, a non-linear PB solver was used [84]. The internal dielectric constant was set to 1, the theoretical salt concentration was chosen to be 100 mM, and a non-bonded cutoff of 12 Å was considered. The electrostatic energy and forces in the system were estimated using the particle–particle–particle–mesh (P3M) method [85]. Structures and figures representing the structural analyses were visualized and drawn using ChimeraX program [86].

#### Statistical method and analysis

Statistical analysis was performed using GraphPad Prism 9. Unless otherwise stated, comparisons between two groups were evaluated using a two-tailed unpaired Student’s *t*-test. Data are presented as mean ± standard deviation (SD) from three independent biological replicates (*n* = 3). A *P*-value < 0.05 was considered statistically significant.

## Results

### Testing the condensation of Z-DNA under known Z-DNA inducers

**Figure 1.**
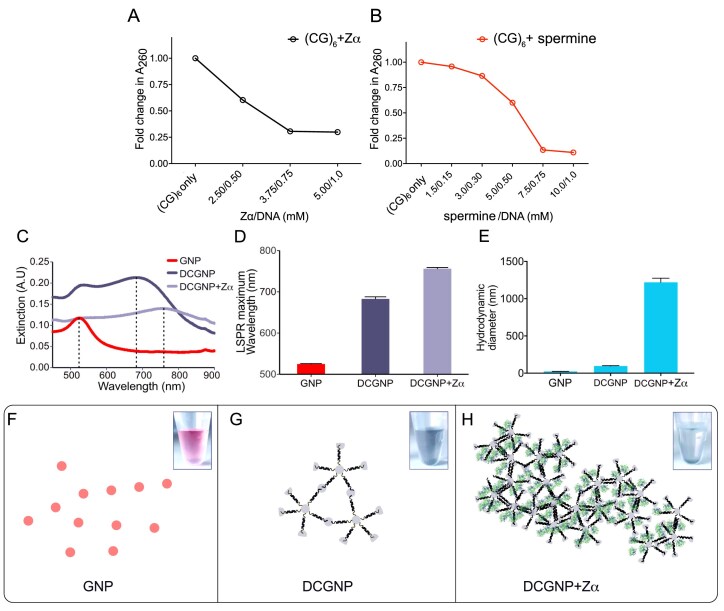
The effect of Z-DNA inducers on DNA condensation and DCGNP. Condensation assay of (CG)_6_ in the presence of Z⍺ protein (**A**) and spermine (**B**) at 5-molar and 10-molar excesses relative to (CG)_6_, respectively. Condensation is displayed as a fold change in DNA absorbance (A_260_) in the solution. (**C**) The UV–Visible extinction spectra of free colloidal 20 nm GNP (red), and DCGNP probes in the absence (dark blue) and presence of Zα (light blue). The vertical dotted line drawn at the LSPR maximum indicates the corresponding wavelength. (**D**) Histogram of the LSPR maximum wavelength from (C). (**E**) The hydrodynamic diameter of GNP and DCGNPs in the presence and absence of Zα measured by DLS. (**F–H**) Schematic representation of GNP (F), DCGNP (G), and DCGNP in the presence of Zα protein (H) with their corresponding colors inset. GNP represented as red circles, aggregate to form a DCGNP (dark blue) when conjugated by a DNA linker and further aggregated when Zα induces the condensation of DNA (DCGNP + Zα, light blue). Labels: Zα, Zα domain from human ADAR1 protein; GNP, 20 nm gold nanoparticle; and DCGNP, DNA-conjugated gold nanoparticle.

Based on the previous studies, we hypothesized that molecules that induce DNA condensation possess the property to stabilize Z-DNA or vice versa. Under this hypothesis, it is plausible to find novel Z-DNA inducers from molecules that induce DNA condensation. To assess this possibility, we first tested if Z-DNA inducers can induce DNA condensation using spermine, a well-known small-molecule Z-DNA inducer, and the Z-DNA binding domain of the human adenosine deaminase RNA-specific 1 protein (hZα_ADAR1_, henceforth called Zα) as model inducers (Fig. 1A and B).

First, we confirmed that both Z-DNA inducers have the B-to-Z inducing activity by CD analyses (Supplementary Fig. S2). We performed condensation experiments using (CG)_6_, double-strand oligonucleotides listed in Table 1, in the presence of Zα and spermine (Fig. 1A and B). The extent of DNA condensation was determined by measuring the amount of free DNA remaining in the supernatant after centrifugation, with condensed DNA precipitated in the presence of the ligand. Our initial experiments with a 10:1 spermine:DNA or a 5:1 Zα:DNA molar ratio at a low micromolar DNA content show less evident DNA condensation. However, when the amount of DNA was increased from micromolar to millimolar amounts, while maintaining the same molar ratio of the Z-DNA inducers to DNA, dramatic condensation of the DNA occurred. With this positive outcome, we confirmed the feasibility of screening Z-DNA inducers via the condensation assay.

### Design, synthesis, and characterization of DCGNP for the condensation assay

For the effective screening of DNA condensation, a more efficient testing system that can accommodate the use of significantly smaller amounts of DNA and small molecules was required, which is essential for testing hundreds to thousands of compounds in a chemical library. Therefore, we prepared DCGNPs as probes to monitor DNA condensation. Given that (CG)_6_ was confirmed to undergo a conformational change from B-DNA to Z-DNA (Supplementary Fig. S2) and exhibited condensation in the presence of Zα or spermine (Fig. 1A and B), we used NZ ODNs (NZ-1 to NZ-4, Table 1), which are self-complementary CG dinucleotide repeats with a 5′ thiol group and a poly-adenine spacer. Furthermore, between the two representative Z-DNA inducers, we primarily utilized Zα for validating the condensation assay system, as it demonstrated a more robust response compared to spermine.

The first DCGNP was made by conjugating 20 nm GNP with NZ-1, carrying a thiol-modified tetra-A spacer (Table 1). While the free GNP showed an extinction maximum at 520 nm, DCGNP functionalized with NZ-1 showed a shift to near 650 nm, while the addition of Z⍺ shifted the extinction maximum further to 730 nm (Fig. 1C and D). This was supported by dynamic light scattering (DLS), which revealed an increase in the hydrodynamic diameter of DCGNP after functionalization with DNA, which was further increased in the presence of Z⍺ (Fig. 1E), with a corresponding change in solution color from red to dark blue (Fig. 1F and G, and inset). Interestingly, incubation of the DCGNP solution with Zα resulted in a light blue colored solution (Fig. 1 H, inset). These results suggest that DCGNP formed colloidal agglomerates (a model in Fig. 1G), as evident by a bathochromic shift (Fig. 1C) and a hydrodynamic change (Fig. 1E) due to the multivalent conjugation of self-complementary NZ-1 to GNP (a model in Fig. 1G), which is similar to the results of previous studies on DNA-conjugated nanoparticles [87–89]. Furthermore, enhanced bathochromic shift and hydrodynamic diameter change in the presence of Zα (Fig. 1C and E) suggest the formation of larger DCGNP agglomerates, accompanied by the condensation of the dsDNA conjugated on the surface of DCGNP (Fig. 1H).

### Monitoring Z-DNA inducer–dependent DNA condensation with DCGNP

**Figure 2.**
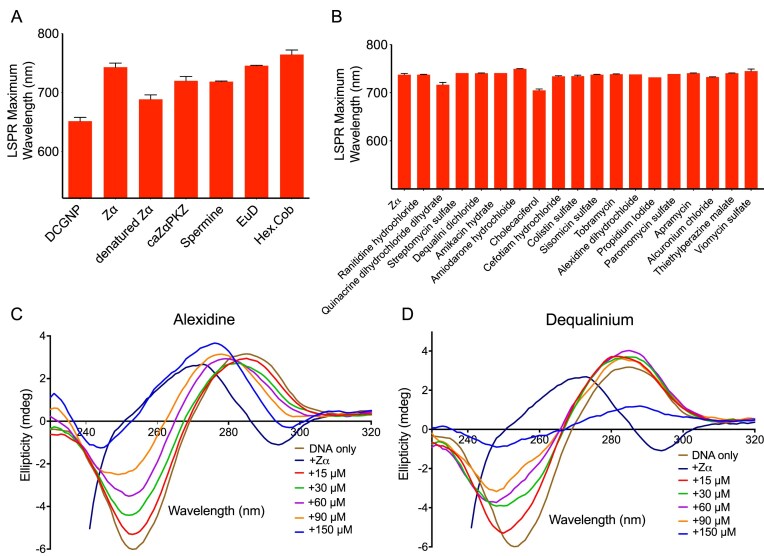
Evaluation of DCGNP probe for screening DNA condensation inducers and validation of positive hits as Z-DNA inducers. (**A**) LSPR maxima of known Z-DNA inducers on DCGNP. (**B**) LSPR maxima of candidate compounds post-screening with the DCGNP probe, showing a red-shifted LSPR maximum comparable (± 5%) to that observed in the presence of Zα, indicating positive hits. (**C** and **D**) CD spectra of 15 µM (CG)_6_ and in the presence of the indicated amounts of candidate Z-DNA inducers – Alexidine (C) and Dequalinium (D). CD spectra of (CG)_6_ in the presence of 60 µM (four molar excess) Zα (+Zα) are also shown as a positive control for the B-to-Z transition. Labels: DNA only, (CG)_6_; caZα, goldfish Zα domain from PKZ protein; EuD, europium-L-aspartate; and Hex. Cob, hexammine cobalt chloride.

The increased size of DCGNP and bathochromic shifts followed by treatment with Z⍺ can be interpreted as Z⍺ inducing the condensation of DNA bound to GNP, and correlated with the change in biophysical character of DCGNP. We further tested this possibility using other known Z-DNA inducers, including the Zα domain of the goldfish PKZ protein (caZα_PKZ_) [61], spermine, europium-l-aspartate (EuD) [90], and hexammine cobalt chloride [91] (Fig. 2A). We used denatured Z⍺ as a negative control. Our experiments showed that DCGNP treated with chemical or protein Z-DNA inducers commonly exhibited an extinction shift comparable to that induced by Zα, while DCGNP with denatured Zα showed less extinction shift (Fig. 2A), suggesting that the bathochromic shifts in DCGNP were highly correlated with the DNA condensation upon treatment of Z-DNA inducers. Thus, DCGNP provides a sensitive readout for ligand-induced DNA condensation and is suitable for identifying Z-DNA inducers.

### Optimization of DCGNP for the screening of DNA condensation inducers

To introduce DCGNP as a screening platform for identifying new DNA condensation inducers, it is necessary to optimize variables such as the lengths of ODNs and the linker, respectively, and confirm their role in the bathochromic shifts. First, we examined the effect of the protein Z-DNA inducer (Zα) on bathochromic shifts in comparison to control proteins: albumin (the most abundant protein with non-specific binding affinity to various molecules) and histone (the most abundant non-specific DNA-binding protein) (Supplementary Fig. S3A and B). We observed that Zα caused the maximum red shift on NZ-1 compared to other control proteins (Supplementary Fig. S3A), while its effect on the bare GNP was neither significant nor different from other control proteins (Supplementary Fig. S3B). These results suggest that the bathochromic shift induced by Zα depends on its Z-DNA inducing ability and occurs specifically with DCGNP. Next, ODNs with different spacer lengths were tested; NZ-1 and NZ-2, containing (CG)_6_ with tetra- (4A) and deca-adenylate (10A) spacers, respectively (Table 1), were conjugated to 20 nm GNP (Supplementary Fig. S3C). In this experiment, NZ-1 and NZ-2 exhibited similar red shifts (Supplementary Fig. S3C) under Z-DNA-inducing conditions by Zα. Finally, varying lengths of CG repeats were tested; NZ-1, NZ-3, and NZ-4, containing 6, 24, and 48 CG dinucleotide repeats, respectively, with a tetra-adenylate spacer (Table 1), were conjugated to 20 nm GNP (Supplementary Fig. S3D). Under the same experimental conditions as NZ-1, DCGNP with NZ-3 showed the highest red shift compared to DCGNP with NZ-1 or NZ-4 (Supplementary Fig. S3D). Finally, different sizes of the GNP were tested: 10, 20, and 40 nm, containing (CG)_24_ and tetra-adenylate spacer (Supplementary Fig. S3E). Under the same experimental conditions as NZ-3, DCGNP made from 40 nm GNP showed the highest red shift compared to that made from 10 to 20 nm. However, based on several practical considerations, we selected 20 nm size of the GNP for the DCGNP preparation. The 20 nm size provided an optimal balance between adequate signal response (∼750 nm for Z⍺), reproducibility across screening iterations, and long-term colloidal stability essential for a screening platform [92]. For a robust two-step screening system like our NanoZ platform, consistency and reproducibility across multiple assay cycles are paramount. While 40 nm GNP offers maximum signal, their aggregation kinetics are less controllable and prone to spontaneous clustering, which would compromise screening accuracy. The 20 nm particles maintained stable performance across all DNA sequences tested (Supplementary Fig. S3) without a baseline drift [93–95]. Based on these results, we conclude that DCGNP with NZ-3 containing (CG)_24_ and tetra-adenylate spacer is suitable for small molecule screening.

### Identification of novel Z-DNA inducers using the NanoZ platform

For the screening of Z-DNA inducers, we introduced the NanoZ platform, consisting of a DNA condensation assay and biophysical validation. In the condensation assay, DCGNPs detect ligand-induced DNA condensation through changes in LSPR, observed as characteristic spectral red shifts. The initial hits identified from this assay were subsequently subjected to a complementary biophysical validation pipeline, including CD spectroscopy, 2AP–BEFA assay, and NMR, to evaluate whether the induced conformational changes are consistent with Z-DNA formation.

#### Identification of the compounds that induce DNA condensation

We employed the DCGNP probe to screen chemical libraries, including the Natural Product Library (800 small molecules) and Prestwick Chemical Library (1200 small molecules), for the identification of novel DNA condensation inducers. A 96-well microplate multiplate reader machine capable of spectrum scanning in the UV–Vis range was used for the screening, which enabled fast recording of extinction spectra in a small reaction volume (50 µl). In the initial NanoZ screening stage, chemicals that induced red-shifted extinction changes comparable to or exceeding the effect of Zα by ± 5% were identified as positive hits. Using this approach, 18 chemicals were selected from a pool of 2000 chemicals (Fig. 2B).

#### Identification of new Z-DNA inducers among DNA condensing inducers by applying CD spectroscopy

CD spectroscopic investigation of the positive hits from screening using DCGNP was conducted using (CG)_6_ (Supplementary Fig. S4A and B). Out of the 18 chemicals, only two, alexidine dihydrochloride (Alexidine) and dequalinium dichloride (Dequalinium), showed a similar propensity to induce the Z-DNA conformation in terms of inverted CD spectra (Fig. 2C and D). ODN induced by Alexidine exhibited the characteristic CD features of Z-DNA, a positive ellipticity at 255 nm and a negative ellipticity at 292 nm, or an increased ellipticity near 255 nm and a decreased ellipticity near 292 nm, compared to the typical CD spectra of B-DNA. However, ODN incubated with Dequalinium showed an upward shift in ellipticity at 255 nm, indicating the presence of *syn* nucleobases, but did not show negative ellipticity at 292 nm. As a negative control, we examined the effects of Alexidine and Dequalinium on (TA)_6_, a double-stranded ODN with B-DNA-forming property (Table 1), using CD spectroscopy (Supplementary Fig. S4C and D). However, Alexidine and Dequalinium did not induce any spectral changes, suggesting that the positive hits identified using the DCGNP solution were stringently filtered using CD spectroscopy. In line with this, we further compared Alexidine with chlorhexidine, a closely related bis-biguanide that shares similar cationic charge, hydrophobicity, and DNA-binding properties. CD measurements showed that, although both compounds cause an upward shift near 255 nm reminiscent of known Z-DNA inducers, chlorhexidine does not generate the accompanying negative ellipticity at 292 nm that is required for a complete B-to-Z CD inversion in the (CG)_6_ motif, and it also failed to emerge as a positive hit in the NanoZ screen (Supplementary Fig. S4E). This chemical-analogy control indicates that Z-DNA induction by Alexidine cannot be explained simply by its bis-biguanide scaffold or general cationic DNA binding, but instead depends on specific structural features that uniquely stabilize the left-handed Z-DNA conformation.

#### Characterization of new Z-DNA inducers by 2AP–BEFA

**Figure 3.**
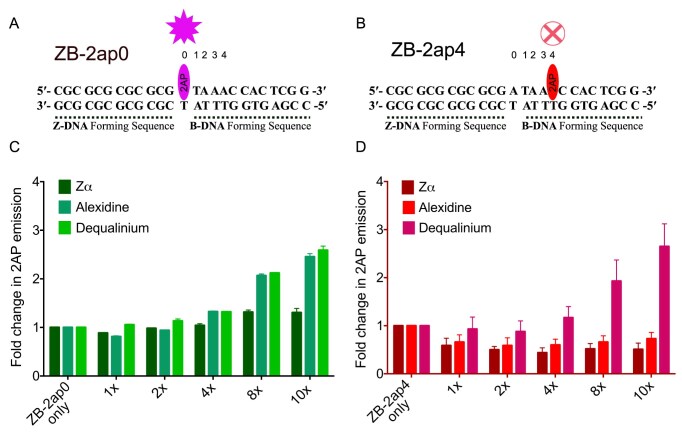
Validation of identified molecules as Z-DNA inducers using 2AP–BEFA assay. (**A** and **B**) Schematic design of the ZB-2ap0 and ZB-2ap4 fluorescent probes used in the 2AP–BEFA assay. (**C** and **D**) Fold change in 2AP fluorescence signal at 380 nm after excitation of 2AP in the ZB-2ap0 and ZB-2ap4 probes at 320 nm in the presence of Zα, Alexidine, or Dequalinium. The values are represented as fold changes relative to the baseline emission from the respective probes in the absence of any Z-DNA inducer.

2AP–BEFA has been successfully used to detect B-to-Z transitions accompanied by Z-DNA formation [68, 96]. We used this assay to test Alexidine and Dequalinium as novel Z-DNA inducers. We used the ZB-2ap0 with 2AP at the base extrusion junction site at position zero (Table 1 and Fig. 3A). As a control, we used a second BZ junction probe, ZB-2ap4 (Table 1 and Fig. 3B), in which the fourth adenine (position four) in the B-DNA section of the probe was replaced with 2AP. The signal from the ZB-2ap4 probe would remain unaffected upon successful Z-DNA induction, while it would emit a signal if there was a non-specific protein–DNA interaction or a conformational change in the B-DNA part of the probe. When Alexidine was incubated with ZB-2ap0 ODN, a strong 2AP signal was observed (Fig. 3C and Supplementary Fig. S5), possibly caused by the extrusion of the 2AP base at the BZ junction site because of Z-DNA formation. A similar spectral change was observed after Dequalinium treatment (Fig. 3C). However, when the ZB-2ap4 ODN was used as a probe for the 2AP–BEFA assay, Alexidine did not cause any 2AP signal emission, whereas Dequalinium showed an increased 2AP signal (Fig. 3D and Supplementary Fig. S5). These results were compared with those of experiments using Zα as a Z-DNA-inducing positive control (Fig. 3C and D, and Supplementary Fig. S5). Taken together, it can be concluded that Alexidine is more likely to have a B-to-Z transition activity than Dequalinium.

### Verification of B-to-Z DNA transition induced by Alexidine by NMR using fluorine-modified CG-rich duplexes

**Figure 4.**
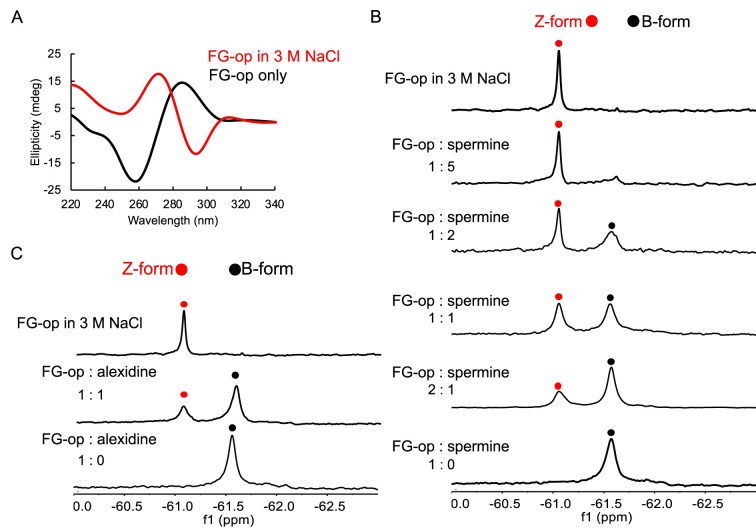
Validation of identified Z-DNA inducers using ^19^F-NMR. (**A**) CD spectrum of FG-op DNA in the presence and absence of 3 M NaCl. (**B** and **C**) ^19^F-NMR titration at the indicated molar excess of spermine (B) or Alexidine (C). FG-DNA was used at 10 µM, and the ratio indicates the molar excess of the respective chemicals used. The reactions were buffered in a solution containing 10 mM NaCl, 1 mM NaPO_4_ (pH 7.0) at 10°C.

Next, we examined Alexidine-induced B-to-Z DNA transition using fluorine-modified CG-rich duplex DNAs, FG-op (6-mer) and FG-sm (8-mer) (Table 1) by ^19^F-NMR spectroscopy. In this experiment, spermine was used as a positive control. To determine the ligand-to-DNA ratio required for the B-to-Z transition, we analyzed the fluorinated duplex DNAs by CD spectroscopy. Even in the absence of spermine, FG-op exhibited a Z-DNA–like CD spectrum in 10 mM NaCl, as reported previously [67]. With stepwise addition of spermine, the characteristic Z-DNA signal became more pronounced and reached saturation at a 5:1 spermine-to-DNA molar ratio (Supplementary Fig. S6A). In the case of the FG-sm duplex, an enhanced negative ellipticity at 295 nm was observed as spermine concentration increased, confirming the B-to-Z transition (Supplementary Fig. S6A). When the same experiments were performed with Alexidine, spectral changes similar to those seen with spermine were observed at lower ligand ratios (Supplementary Fig. S6B). However, at ratios exceeding 1:1 (Alexidine:DNA), the CD spectra progressively flattened, indicating DNA condensation rather than further Z-DNA stabilization, which is consistently reported in the previous studies [51, 88, 89]. As a control, we confirmed the B-to-Z transition of both FG-op and FG-sm in the presence of 3M NaCl (Fig. 4A and Supplementary Fig. S6C).

After confirming the transition by CD, we performed ^19^F-NMR spectroscopy to monitor the characteristic B-to-Z conversion. In FG-op DNA, only one signal was observed at −61.5 ppm in the absence of spermine (Fig. 4B), indicating the presence of B-form DNA. However, a new signal appeared (−61.2 ppm) when spermine was added into the FG-op DNA solution (Fig. 4B). The new signal is clearly observed at a two-fold molar excess of spermine to FG-op (Fig. 4B). The intensity of this new signal becomes the prominent signal while the previous peak disappears completely at the ratio of 1:5 FG-op: spermine. The emergent peak matched the ^19^F spectrum obtained in 3 M NaCl (Fig. 4B), consistent with Z-DNA formation inferred from CD (Fig. 4A). Additionally, in the case of FG-sm DNA, two ^19^F-NMR peaks are evident in the absence of spermine (−61.7 and − 61.9 ppm), representing B-DNA structure (Supplementary Fig. S6D). The two ^19^F-NMR peaks result from two asymmetric ^F^G (8-trifluoromethyl-2′-deoxyguanosine) due to their different positions within the 8-mer duplex sequence. With increasing spermine/DNA ratio, the two peaks characteristic of B-DNA progressively decreased and ultimately disappeared, while two intense new peaks emerged at −61.3 and −61.4 ppm (Supplementary Fig. S6D). The same pattern was observed in 3 M NaCl, consistent with Z-DNA formation indicated by the CD spectrum (Supplementary Fig. S6C). Similarly, ^19^F-NMR spectroscopy was used to monitor Alexidine-induced B-to-Z DNA transition. In the 6-mer FG-op DNA, only one signal was observed at −61.5 ppm in the absence of Alexidine (Fig. 4C), indicating that the B-DNA was confirmed by the CD result (Supplementary Fig. S6B). A new signal appears (−61.1 ppm) when Alexidine was added to the DNA solution at a 1:1 ratio (Fig. 4C). This signal matches the chemical shift observed for the duplex in 3 M NaCl (Fig. 4C) and is consistent with a Z-DNA–like conformational state under our conditions, in agreement with the CD results (Fig. 4A). Additionally, in case of 8-mer FG-sm DNA, two ^19^F-NMR signals were observed in the absence of Alexidine (−61.7 and − 61.9 ppm), representing B-form structure (Supplementary Fig. S6E). Two ^19^F-NMR peaks result from two asymmetric ^F^G due to their different positions within the 8-mer duplex sequence. With increasing molar excess of Alexidine to FG-sm DNA, two new peaks appeared (−61.3 and −61.4 ppm) (Supplementary Fig. S6E). These peaks exhibit chemical shifts similar to those observed in 3 M NaCl (Supplementary Fig. S6E) and are consistent with a Z-DNA–like conformational transition, supported by the corresponding CD measurements (Supplementary Fig. S6C).

To quantify the extent of this conformational conversion, we assigned the peak at −61.1 ppm to Z-form DNA and the peak at −61.5 ppm to B-form DNA in the FG-op DNA and determined the fraction of Z-DNA at each Alexidine: DNA ratio from the relative peak intensities (Supplementary Fig. S6F and G). These analyses show that Alexidine drives a progressive and efficient shift from B- to Z-form comparable to that observed with spermine, supporting its robust B-to-Z transition activity.

#### Identification of alexidine as a new Z-DNA inducer using the NanoZ platform

From the combined efforts of DCGNP, CD spectroscopy, the 2AP–BEFA assay, and the ^19^F-NMR experiments on the NanoZ platform, we identified Alexidine as a small molecule that promotes structural transitions consistent with Z-DNA in CG-rich sequences. Although Dequalinium can induce the conformational change of DNA, it cannot be defined as a Z-DNA inducer since it also affects the conformation of B-DNA (Fig. 3D and Supplementary Fig. S5). To further confirm the nature of the DNA conformation induced by this new chemical, we monitored the CD spectra of the ZB-2ap0 or ZB-2ap4 induced by Alexidine. The CD spectral changes upon Alexidine treatment were consistent with a B-to-Z transition (increased ellipticity near 255 nm and decreased ellipticity near 292 nm relative to B-DNA), although the changes were less pronounced than those induced by Zα (Supplementary Fig. S5D and H). These observations were further supported by our ^19^F-NMR experiments (Fig. 4 and Supplementary Fig. S6). Particularly, Alexidine induces the characteristic B-to-Z CD signature selectively in CG-rich (CG)_6_ DNA, but not in TA-rich (TA)_6_ DNA, demonstrating sequence-dependent stabilization of Z-DNA-forming motifs (Fig. 2C and D, and Supplementary Fig. S4C and D). In addition, orthogonal readouts, including the 2AP–BEFA base-flipping assay and negative controls such as Dequalinium, distinguish the B-to-Z transition signature from non-specific DNA condensation, indicating that Alexidine induces sequence-dependent Z-DNA formation rather than general DNA compaction (Fig. 3C and D, and Supplementary Fig. S5). Consistent with these observations, equilibrium binding measurements showed that Zα binds (CG)_6_ duplex DNA with a dissociation constant of 1.05 µM, whereas Alexidine binds the same sequence with a *K*_D_ of 8.78 µM, indicating micro-molar binding affinities with CG-rich duplexes (Supplementary Fig. S7). Although Zα binds more tightly than Alexidine, the weaker-binding small molecule is nevertheless sufficient to induce robust B-to-Z transition and DNA condensation. Taken together, these results support the conclusion that Alexidine promotes structural transitions consistent with Z-DNA and can serve as a cell-active chemical modulator in this context.

### Characterization of alexidine as the novel Z-DNA inducer

#### Alexidine can increase the agglomeration of DCGNP

Since Z-DNA inducing molecules, such as Z⍺ enhanced the agglomeration of DCGNP (Fig. 1C), we also tested this capability of Alexidine as well as spermine by light microscopy, in addition to measuring the hydrodynamic size (Supplementary Fig. S8A–C, and Supplementary Movie S1). Consistent with the study of Zα, we observed that both spermine and Alexidine can enhance the agglomeration of DCGNP, easily visible under light microscopy. Alexidine demonstrated a stronger agglomeration activity of DCGNP compared to spermine.

#### Alexidine can induce the condensation of DNA

Having confirmed Alexidine-induced agglomeration of DCGNP, we next examined whether Alexidine directly promotes DNA condensation. As with spermine-driven condensation of (CG)_6_ (Fig. 1B), Alexidine robustly condensed (CG)_6_ (Supplementary Fig. S8D). Notably, under these conditions, spermine showed little or no activity, indicating that Alexidine is a more potent inducer in this assay. In addition, we further tested whether Alexidine also has DNA-condensation activity on larger DNA and therefore evaluated salmon sperm DNA (Supplementary Fig. S8E). Alexidine also condensed salmon sperm DNA, an effect observed with spermine as well, though to a lesser extent than with Alexidine (Supplementary Fig. S8E). Collectively, these data indicate that Alexidine condenses DNA irrespective of sequence or length, similar to the well-known Z-DNA inducer spermine.

#### Alexidine can induce the Z-DNA formation in cells

**Figure 5.**
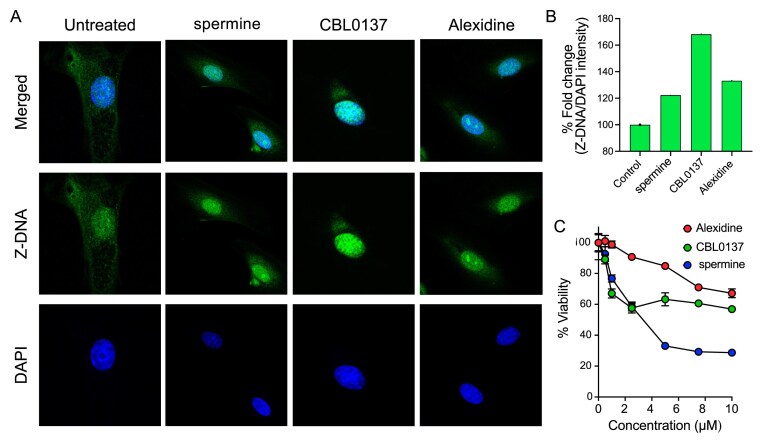
Validation of Alexidine as a Z-DNA inducer in cells. (**A**) Immunostaining for Z-DNA in untreated MEFs and treated with spermine (5 µM), CBL0137 (5 µM), or Alexidine (1 µM) for 24 h. (**B**) Quantification of fluorescent intensity of Z-DNA signals relative to the nuclear stain (DAPI) (*n* = 10). (**C**) Dose-dependent cytotoxicity of MEFs treated with different concentrations of Z-DNA inducer for 24 h.

One of the objectives of this study was to identify small-molecule Z-DNA inducers that could be used to modulate the structure and function of Z-DNA in cells. The finding that Alexidine is a novel Z-DNA inducer highlights that the probable mechanism of its action may involve the induction and stabilization of Z-DNA in cells. To investigate the Z-DNA-inducing activity of the newly identified Z-DNA-inducing small molecule, we treated Alexidine to MEFs and quantified the amount of Z-DNA in the genome using Z22, an anti-Z-DNA antibody (Fig. 5A and B) as reported previously [97]. We used spermine and CBL0137, well-known Z-DNA inducers in cells [97], as controls. All three small molecules including Alexidine increased nuclear Z-DNA signals relative to untreated controls, indicating efficient cellular uptake and genomic Z-DNA induction (Fig. 5A and B). In parallel, we verified that the concentration of Alexidine used for cellular Z-DNA induction does not elicit cytotoxicity in MEFs, as 1 µM Alexidine treatment for 24 h showed no significant loss of cell viability (Fig. 5C). This working concentration is substantially lower than the compound’s IC₅₀, indicating that the observed Z-DNA induction is attributable to specific Z-DNA stabilization rather than non-specific cytotoxic stress (Fig. 5C).

### Functional consequences of alexidine-induced Z-DNA formation in cells

#### Alexidine-induced Z-DNA colocalizes in repeat regions bound by PolR2A

**Figure 6.**
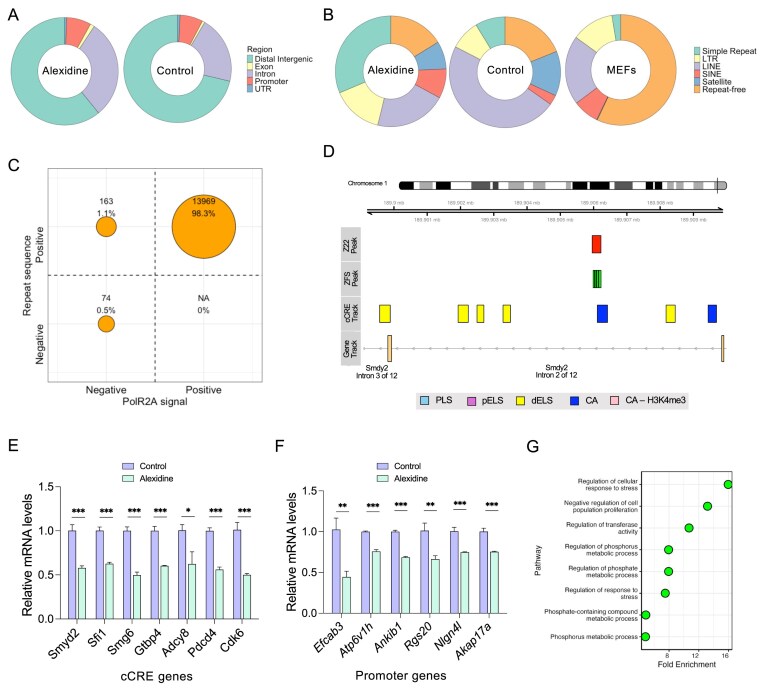
Cellular effects of Z-DNA induced by Alexidine in MEFs. (**A**) Distribution of genomic locations of Z22-enriched peaks following Alexidine treatment (1 μM) for 24 h compared to untreated MEFs. (**B**) Proportion of repeat elements in the mouse genome (right, MEFs) compared with the distribution of Z22 pulldowns in untreated (middle) and 1 μM Alexidine-treated (left) MEFs. (**C**) Quadrant plot showing the distribution of Z22-enriched peaks from ChIP of 1 μM Alexidine-treated MEFs. The *x*-axis denotes PolR2A occupancy status, and the *y*-axis indicates the presence or absence of repeat sequences. The size of the circle is proportional to the number of peaks in each quadrant, with absolute counts shown inside each circle. (**D**) Representative genome browser view of Z22 ChIP-seq peak in Alexidine-treated MEFs overlapping with Z-hunter predicted Z-DNA forming sequence (ZFS), cCREs, and gene annotation. (**E** and **F**) RT-qPCR analysis of representative genes whose genomic regions overlapped with Z22-enriched (E) cCREs or (F) promoters in Alexidine-treated MEFs (*n* = 3). (**G**) Pathway enrichment analysis of genes whose genomic regions overlapped Z22-enriched loci following Alexidine treatment in MEFs.

To identify the genomic locations of Z-DNA induced by Alexidine treatment in MEFs, immunoprecipitation was performed with Z22 antibody and sequenced. Relative enrichment of Z22 peaks was observed in the introns and promoter regions in Alexidine-treated cells compared to the untreated cells (Fig. 6A). Since Z-DNA has a higher propensity to form at alternating purine-pyrimidine repeats [98–100], we further analyzed the class of repeat elements present in the Z22 peaks. In comparison to the normal mouse genome, which majorly contains repeat-free regions, Z22 pulldowns in both the untreated and Alexidine-treated cells showed relative enrichment of multiple repeat elements, the majority being LINE, LTR, and simple repeats (Fig. 6B). In particular, Alexidine-treated cells show considerable enrichment of simple repeats and LTRs compared to the untreated cells pulled down with Z22. In addition, the top 10 motifs found in the Z22 peaks of Alexidine-treated cells were long stretches of TG repeats, a typical sequence profile found in Z-DNA-forming regions (Supplementary Fig. S9) [101, 102]. Because negative supercoiling downstream of RNA polymerase II is known to favor Z-DNA formation [103, 104], we further analyzed whether the repeat regions colocalize with PolR2A (gene encoding the larger subunit of RNA polymerase II) – positive sites. It was found that 98% of the Alexidine-treated cells’ Z22 peaks consisted of repeat sequences and were positive for PolR2A signal, suggesting a role of Alexidine-induced Z-DNA in transcriptional activity (Fig. 6C).

#### Alexidine-stabilized Z-DNA inhibits gene expression

Given the importance of promoters and cCREs (candidate *cis*-regulatory elements) in transcriptional regulation, we next investigated several candidate genes whose regulatory regions overlapped with ChIP-seq peaks from Alexidine-treated cells and assessed their transcriptional activity (Fig. 6D). All the genes assessed in the cCRE and promoter regions were downregulated upon Alexidine treatment (Fig. 6E and F). However, there was no significant change in gene expression when treated with the known Z-DNA inducer spermine or the non-Z-DNA inducer Dequalinium (Supplementary Fig. S10). In addition, analysis of the GO terms associated with the closest genes of the Z22 peaks from Alexidine-treated cells revealed enrichment of pathway terms related to cell cycle and response of cells to cell stress (Fig. 6G). Overall, current results suggest that Alexidine specifically binds to transcriptionally active purine–pyrimidine repeat-rich regions in the genome, resulting in inhibition of gene expression.

### Investigation of the binding mode of Alexidine to Z-DNA by computational approaches

#### The binding mode of Alexidine to Z-DNA

**Figure 7.**
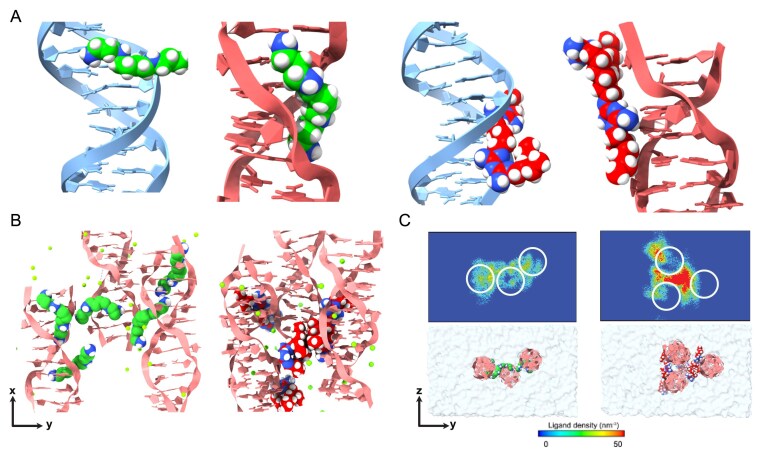
MD simulation-based verification of side-by-side attraction of Z-DNA/B-DNA in the presence of spermine and Alexidine. Final MD snapshot at 50 ns showing ligands in the presence of a (**A**) single duplex of Z-DNA/B-DNA, from left to right—spermine/B-DNA, spermine/Z-DNA, Alexidine/B-DNA, and Alexidine/Z-DNA, (**B**) three duplexes of Z-DNA, from left to right—spermine/Z-DNA and Alexidine/Z-DNA. (**C**) Top: Ligand density map of spermine (left) and Alexidine (right) projected along the Z-DNA helical axes (*x*-axis), with DNA represented as white circles. Bottom: Representative simulation models of the three Z-DNA duplexes system in an explicit water box, oriented to match the density maps above. DNA is shown as ribbon models (B-DNA, blue; Z-DNA, pink), and ligands are shown as space-filling models (carbon atoms in green for spermine and red for Alexidine).

To computationally evaluate the binding mode of the Z-DNA inducing ligands to DNA, we constructed a single-duplex MD system comprising either one copy of B-DNA of (CG)_10_ or Z-DNA of (CG)_12_, each representing nearly one turn of the B-DNA or Z-DNA conformations, respectively, in a simulation box. MD simulations were then conducted by introducing one molecule of the Z-DNA inducing ligands at a random position within the simulation box. An effectively infinite DNA configuration across the periodic boundary condition was considered to eliminate the effects of bending fluctuations, strand crossover, and base-stacking interactions of the ligand, as the simulations aimed to validate only the binding mode of ligands to DNA (B-DNA/Z-DNA) [105, 106]. First, MD simulations were performed with spermine as a ligand because its binding mode to B-DNA/Z-DNA is characterized by its crystal structures, which serve as positive controls (Supplementary Fig. S11A and C, left columns) [107, 108]. MD analysis revealed a major groove binding mode of spermine to a single duplex of B-DNA, and groove binding to Z-DNA (Fig. 7A, two models on the left). The developed MD system was well validated since the binding mode of spermine to B-DNA/Z-DNA revealed by MD analysis (Supplementary Fig. S11B and D, left columns) closely replicates the ligand-binding modes in the crystal structure (Supplementary Fig. S11A and C, left columns). Owing to their similarity in size and charge properties, we hypothesized that Alexidine has a binding mode comparable to that of spermine. Accordingly, Alexidine was also simulated in the presence of B-DNA/Z-DNA in the same way as the MD simulation of the spermine/DNA complex by introducing one molecule of Alexidine at a random position within the simulation box (Supplementary Fig. S12A and B, left columns). Alexidine was found to bind to both B-DNA and Z-DNA via minor groove and groove binding, respectively (Fig. 7A, two models on the right). Furthermore, MM-PBSA binding energy calculations from snapshots taken over the final 10 ns of the 50 ns MD simulation indicate that Alexidine binds more favorably to a Z-DNA duplex than to B-DNA, as evidenced by the lower MM-PBSA binding energy in Table 2, consistent with its greater stabilizing effect on Z-DNA [82].

**Table 2.** DNA–ligand binding energy

DNA–ligand binding energy (kcal/mol)				
Ligand	DNA			
	1 Helix		3 Helices	
	Z-DNA	B-DNA	Z-DNA	B-DNA
Spermine	−7.74 ± 1.19	−7.39 ± 1.41	−5.64 ± 2.15	−12.46 ± 1.37
Alexidine	0.25 ± 0.77	10.11 ± 1.49	−8.67 ± 0.33	−5.46 ± 0.30

The binding energy (kcal/mol) of ligands to a single duplex or three duplexes of Z-DNA/B-DNA was calculated from the MD snapshots extracted from the last 10 ns of the 50 ns MD simulation at an interval of 500 ps using the MM-PBSA method.

#### Alexidine induces the side-by-side Z-DNA attraction

Furthermore, to identify the ability of the ligands to induce the DNA condensation or DNA–DNA attraction, we modified the single-duplex MD system to a three-duplex MD system by adding three dsDNA duplexes at an inter-duplex distance (center of mass, COM) of 2 nm (Supplementary Fig. S13). The MD simulations were performed in the presence of five spermine/Alexidine molecules added to the simulation box at random locations. At the end of 50 ns, in the presence of either of the ligands, Z-DNA was found to be attracted towards each other (Fig. 7B and C), supporting our view that Z-DNA-inducing ligands cause DNA condensation via side-by-side association of DNA. Model of the bound ligands to Z-DNA viewed along the z-axis (Fig. 7B) and density maps of ligands projected onto the *y*–*z* axis produced by averaging 100 snapshots taken during the last 1 ns of the simulation period revealed that spermine bound to the groove and inter-helical region of Z-DNA, whereas Alexidine was predominantly localized only in the inter-helical region of Z-DNA, bringing them closer by associating the DNA pair (Fig. 7C). In this model, spermine rendered a long chain-like arrangement of Z-DNA, whereas Alexidine mediated the formation of a more confined packing of the helices (Supplementary Fig. S14A and B), and the ligands were found in closer proximity to DNA than to the counter ions, suggesting ligand-mediated DNA–DNA attraction (Supplementary Fig. S14C and D). Furthermore, analysis of the hydrogen bonds between the ligands and Z-DNA validated the well-known groove binding (spermine 2 and spermine 4) and the inter-Z-DNA bridging behavior of spermine, typically through phosphate backbones (Supplementary Fig. S15A). In contrast, Alexidine was found to form a higher number of hydrogen bonds with nucleobases (N7 of guanine) and a higher absolute number of hydrogen bonds with Z-DNA than spermine (Supplementary Fig. S15B). Furthermore, MM-PBSA binding energy calculations using the MD snapshots extracted from the last 10 ns of the 50 ns MD simulation at an interval of 500 ps revealed that the binding of Alexidine to Z-DNA was much stronger than that of spermine, as characterized by a more negative enthalpy change (Table 2) [83]. As expected, the binding of Alexidine to Z-DNA was much stronger than the binding of Alexidine to B-DNA, further validating that Alexidine is a strong Z-DNA inducer (Supplementary Figs S14 and S16).

The inter-Z-DNA distance plays a vital role in describing the strength of the inter-Z-DNA attraction. Since it is now clear that Alexidine not only binds strongly to Z-DNA but also induces Z-DNA attraction, we further evaluated the inter-Z-DNA attraction energy in the presence of ligands by umbrella sampling by varying the distances between the two duplexes of Z-DNA from 1.6 to 3.5 nm (Supplementary Fig. S17). In the absence of ligand, the global minima of the system was found at 1.7 nm with a local minima at 2.0 nm which is in close accordance with the distances required for the formation of direct contacts between the Z-DNA via salt bridges as reported by Li *et al. *[59]. However, in the presence of Alexidine, the global minima of the system shifted to 1.8 nm characterized by a deep potential well of −5.6 kcal/mol accounting for the ligand bridging in addition to a second potential well at around 2.4 nm corresponding to the inter-Z-DNA distance obtained in our classical MD simulations (Supplementary Fig. S17). In the presence of spermine, a similar attractive potential was observed at 1.85 nm with a second potential at 2.2 nm (Supplementary Fig. S17).

#### Alexidine induces aggregation of Z-DNA arrays

Furthermore, we intended to computationally mimic the experimental conditions of the study characterized by the DNA condensation in the presence of Alexidine and spermine (Fig. 1A and B, and Supplementary Fig. S8D and E), which correlated well with the resulting hydrodynamic diameter of the agglomerates. To achieve this, we modified the MD system with 64 copies of DNA arranged in an 8 × 8 array with an inter-duplex distance (COM) of 2.8 nm (Supplementary Fig. S18). Using this system, we analyzed the ligand density map and the distances of ions and ligands from the duplex DNA at low (40 Alexidine and 40 spermine molecules, respectively) (Supplementary Figs S19 and S20) and maximum possible ligand density (322 Alexidine and 500 spermine molecules, respectively) (Supplementary Fig. S21). These results are consistent with the observations described in the previous section obtained from MD simulations using the three-duplex DNA system (Fig. 7B and C). However, with the increased number of duplex DNA molecules in the new simulation system, we found that the distance between the COM of DNA to the ligands and the ions drastically diminished at the maximum possible ligand density in the system relative to the low ligand density simulation (Supplementary Fig. S22). The simulation results with the maximum possible ligand density in the 64-duplex DNA system (Supplementary Fig. S21) further support our experimental results and the view that the ligands induce DNA condensation.

## Discussion

DNA condensation is a fundamental phenomenon with wide-ranging roles in biology, including genome organization and packaging, gene regulation, gene-therapy systems, nanoparticle assembly, DNA self-assembly, and other higher-order structures [109]. This is facilitated by various factors, predominantly the presence of multivalent cationic small molecules [110, 111]. In this study, our experimental data demonstrate that bona fide Z-DNA inducers promote DNA condensation of CG-rich sequences (Fig. 1), and on this basis, we developed the NanoZ platform consisting of a DCGNP-based condensation sensor coupled to orthogonal spectroscopic validation and cell-based Z-DNA profiling as a sensitive discovery and characterization workflow. Using this screening platform, we experimentally showed that Alexidine as a cell-active Z-DNA inducer and that it drives B-to-Z conversion *in vitro* and increases Z-DNA formation in cells. We then propose a conceptual model in which the same tools that establish Alexidine as a Z-DNA inducer in cells also enable us to explore the hypothesized functional consequences of Z-DNA formation, namely transcriptional repression at promoters and cCREs, which we observe as a robust correlation in our genome-wide and qRT-PCR analyses.

DNA conjugation to the nanoparticles has been widely used for various purposes, including sensing, imaging, and diagnostics, because DNA self-assembly induces GNP agglomeration in response to environmental changes or the binding of ligands to the complex, which results in a bathochromic (red) shift in the LSPR of GNPs. In this study, DCGNP prepared with Z-DNA-forming self-complementary DNA functionalized on GNPs was introduced for screening DNA condensation inducers. In general, large numbers and long lengths of ODN molecules bound to each GNP surface are critical for the sensitivity and stability of DCGNP [112]. However, we did not observe improved sensing with a long 96 bp ODN (Supplementary Fig. S3), suggesting that the agglomeration of DCGNPs can be affected by various unknown factors, which can be further studied. We observed a size increment, line broadening of the UV–Vis spectra, and a bathochromic shift of the extinction maximum in DCGNP when DNA was conjugated onto the GNP surface (Fig. 1C and D). Therefore, we believe that the observed biophysical changes were caused by the clustering of NPs via the multivalent conjugation of DNA on the GNP surface, as shown in the scheme (Fig. 1G). Further enhanced agglomeration and red shift of UV–Vis spectra in the presence of Z-DNA inducers can be explained by the condensation of DNA, as shown in the model in Fig. 1H. We considered estimating DNA surface coverage on the DCGNPs using the UV–Vis method of Wang *et al.* [67], but obtained unrealistically high values (>10³ strands per 20 nm particle), consistent with NanoZ’s GC-rich, palindromic sequence promoting interparticle crosslinking rather than monodisperse monolayers. We therefore follow Taton’s geometric maximum-density approach to define DNA input per nanoparticle and, after removing excess thiolated ODNs by centrifugation, do not report an absolute “DNA per particle” value from UV–Vis measurements in this system.

The Z-DNA conformation is characterized by a unique zig–zag arrangement of the sugar phosphate backbone that enforces the proximity of the repulsive negative charges on the phosphates, and the nitrogenous bases are arranged toward the outside of the helix [113]. In addition, the co-crystal structures of DNA molecules in Z-DNA conformation tend to associate side-by-side due to attraction force enabled by phosphate bridges formed in the presence of Na^+^, Mg^2+^, or Z-DNA inducers like spermine that act like mortar between “DNA bricks” (Supplementary Fig. S11D), along with the hydrophobic interactions of the deoxy ribose sugar [114, 115]. Thus, Z-DNA might easily arrange in a side-by-side association, where the inducers act as a glue holding the individual Z-DNA molecules together. This hypothesis was supported by the computational analyses of Alexidine bound to Z-DNA. MD simulations of the complex revealed that the density of the ligands bridging the helices was higher with Alexidine than with spermine (Fig. 7C and Supplementary Figs S19–21). In the bridging model of DNA attraction [116, 117], it was proposed that the presence of a higher density of bridging polycations promoted stronger DNA attraction, which is consistent with what was observed in the computational models (Fig. 7C and Supplementary Fig. S21). Therefore, both computational and experimental approaches consistently support the idea that Alexidine promotes inter-Z-DNA attraction, as observed in the case of spermine. The lower binding energies of Alexidine to Z-DNA compared to spermine are in good agreement with our experimental observations, where we observed visible condensation of DNA in the presence of Alexidine, but to a lesser extent in the presence of spermine (Table 2, and Supplementary Fig. S8D and E).

Z-DNA is a transient, high-energy structure that is extremely challenging to study [118]. The Z-DNA conformation can be stabilized by several factors, including Z-DNA-binding proteins [5], organic solvents [119], a high ionic environment [2], negative supercoiling [120], base modification [121], and other chemicals such as polyamines [122] and porphyrin derivatives [123]. However, these Z-DNA inducers have not materialized as intervention strategies in the study of Z-DNA physiology implicated in several diseases, such as gene regulation, recombination events, and immune responses. Therefore, additional Z-DNA regulators are required for Z-DNA studies. Although there are studies that report individual Z-DNA modulators, there have been no serious attempts at large-scale screening, owing to the lack of methods for detecting conformational changes in DNA in a high-throughput setting. From this point of view, the NanoZ screening platform provides a new opportunity to screen for Z-DNA inducers with advantages in terms of time, cost, and efficiency. Positive hits, ligands that induce DNA condensation, are rapidly identified on the DCGNP sensor by monitoring ligand-induced spectral shifts. The putative false positives are then removed by orthogonal validation with CD and 2AP–BEFA, and, ultimately, ^19^F-NMR provides definitive confirmation of B-to-Z transition activity (Figs 3–5). In this study, we show that NanoZ not only enables rapid identification of Z-DNA modulators (Fig. 2) but also yields a cell-active, functionally validated Z-DNA inducer, which is associated with genomic Z-DNA formation and altered gene expression (Figs 5 and 6), thereby underscoring the platform’s utility. However, although the convergent results from CD spectroscopy, 2AP–BEFA, and ^19^F-NMR strongly support structural transitions consistent with Z-DNA, these approaches do not provide atomic-resolution structural confirmation. Therefore, we cannot formally exclude the possibility of mixed or Z-like non-canonical conformations under the present experimental conditions.

Alexidine is an alkyl bis(biguanide) antiseptic recognized by the US Food and Drug Administration (FDA) and widely used as a broad-spectrum antimicrobial agent [124]. Alexidine is also known to act as a potent and selective inhibitor of PTPMT1 and has been reported to induce apoptosis in cancer cell lines [125, 126]. In our study, we found that Z-DNA was preferentially induced by Alexidine at repeat-rich regions of the mouse genome, many of which were associated with or located near PolR2A-positive promoters and cCREs (Fig. 6C and D). Stabilization of Z-DNA by Alexidine in these regulatory regions was associated with transcriptional inhibition (Fig. 6E and F). Previous studies have shown that Z-DNA formation is dynamically associated with transcription, particularly at promoter and enhancer regions, where negative supercoiling generated during transcription facilitates B-to-Z transition [120]. Such transient Z-DNA structures have often been linked to transcriptional activation or chromatin accessibility.

In contrast, our results demonstrate that Alexidine-induced Z-DNA forms preferentially at repeat-rich, PolR2A-positive promoters and cCREs, and that its stabilization is associated with transcriptional repression (Fig. 6). One possible explanation for this observation is that the compound stabilizes B*/Z*-like intermediates, analogous to those proposed for protein-induced B-to-Z transitions, thereby facilitating the formation of extended left-handed tracts. In addition, the condensation and inter-helical bridging activity observed in our MD simulations suggest a potential, but as yet untested, mechanism by which Alexidine could lower the energetic barrier for such transitions. A second, non-exclusive hypothesis is that Z-DNA-binding proteins, such as ADAR1 or ZBP1, participate in relaying Alexidine-induced structural changes to the transcriptional machinery or chromatin regulators. Our current data do not directly address the involvement of specific Z-DNA readers, and we did not observe Alexidine-dependent enrichment at the loci encoding ADAR1 or ZBP1 in the Z22 ChIP-seq data. Future experiments will be required to test whether Alexidine recruits, redistributes, or functionally modulates these factors or other Z-DNA-interacting proteins.

This finding is consistent with previous reports showing that Z-DNA can act as a transcriptional silencer, as observed in the ADAM12 gene [14]. Together, these observations suggest that while transient Z-DNA may participate in transcriptional activation, persistent or chemically stabilized Z-DNA may exert an opposite regulatory effect, potentially by interfering with transcriptional machinery or chromatin organization. Further studies will be required to determine whether the observed repression arises from Z-DNA stabilization itself or from Alexidine binding to Z-DNA and its associated factors. Interestingly, Alexidine has been reported to trigger apoptosis and cell-cycle inhibition in various cancer systems [116, 117]. In parallel, our data indicate Z-DNA-linked repression of gene expression, including cell-cycle pathways (Fig. 6G). Therefore, it warrants investigation whether the Z-DNA-mediated regulation by Alexidine observed here contributes to its previously described anti-cancer mechanisms.

Taken together, we demonstrate that the NanoZ platform is an effective method for identifying new Z-DNA-inducing ligands. Through the identification and validation of Alexidine as a novel Z-DNA inducer, we demonstrated that the NanoZ platform is fast, cost-effective, and highly efficient, making it suitable for screening a wide variety of small-molecule libraries, irrespective of their physicochemical nature. However, NanoZ should be viewed as a platform optimized to identify Z-DNA inducers that are coupled to DNA condensation under the screening conditions. Consequently, Z-DNA stabilizers that do not promote measurable condensation or DCGNP agglomeration may not be captured by this platform and could represent a potential false-negative class. Importantly, by validating the cellular activity of NanoZ-identified Alexidine, we revealed for the first time that chemically induced Z-DNA formation can repress transcription in specific genomic contexts, uncovering an unrecognized regulatory role of Z-DNA in gene expression and thereby highlighting the potential of the NanoZ platform for Z-DNA studies. We believe that the simplicity and efficacy of the NanoZ platform will expand the Z-DNA chemical biology field, allowing the discovery of Z-DNA inducers and disruptors. Therefore, this platform can aid Z-DNA-targeted studies in cells and organisms of interest, leading to a better understanding of the physiological consequences of Z-DNA formation in cells.

## Supplementary Material

gkag281_Supplemental_Files

## Data Availability

The ChIP-seq experimental data are available from the Gene Expression Omnibus database (https://www.ncbi.nlm.nih.gov/geo) under the accession number GSE312055. UCSC Genome Browser session files for visualization of ChIP-seq tracks are available at https://genome-asia.ucsc.edu/s/kanshrute/Alex%2DChIP. The final small molecule–DNA complex structures from the MD simulations have been deposited in the Zenodo repository (https://doi.org/10.5281/zenodo.17402809). Derived data supporting the findings of this study are available from the corresponding author (K.K.K) on request.

## References

[1] Ghosh A, Bansal M. A glossary of DNA structures from A to Z. Acta Crystallogr D Biol Crystallogr. 2003;59:620–6. 10.1107/S0907444903003251. 12657780

[2] Pohl FM, Jovin TM. Salt-induced co-operative conformational change of a synthetic DNA: equilibrium and kinetic studies with poly (dG-dC). J Mol Biol. 1972;67:375–96. 10.1016/0022-2836(72)90457-3. 5045303

[3] Lafer EM, Sousa R, Rosen B et al. Isolation and characterization of Z-DNA binding proteins from wheat germ. Biochemistry. 1985;24:5070–6. 10.1021/bi00340a017. 4074677

[4] Zhang S, Lockshin C, Herbert AG et al. Zuotin, a putative Z-DNA binding protein in *Saccharomyces cerevisiae*. EMBO J. 1992;11:3787–96. 10.1002/j.1460-2075.1992.tb05464.x. 1396572 PMC556839

[5] Herbert AG, Alfken J, Kim YG et al. A Z-DNA binding domain present in the human editing enzyme, double-stranded RNA adenosine deaminase. Proc Natl Acad Sci USA. 1997;94:8421–6. 10.1073/pnas.94.16.8421. 9237992 PMC22942

[6] Schwartz T, Behlke J, Lowenhaupt K et al. Structure of the DLM-1-Z-DNA complex reveals a conserved family of Z-DNA-binding proteins. Nat Struct Biol. 2001;8:761–5. 10.1038/nsb0901-761. 11524677

[7] Kim YG, Muralinath M, Brandt T et al. A role for Z-DNA binding in vaccinia virus pathogenesis. Proc Natl Acad Sci USA. 2003;100:6974–9. 10.1073/pnas.0431131100. 12777633 PMC165815

[8] Kim D, Reddy S, Kim DY et al. Base extrusion is found at helical junctions between right- and left-handed forms of DNA and RNA. Nucleic Acids Res. 2009;37:4353–9. 10.1093/nar/gkp364. 19465399 PMC2715235

[9] Kim K, Khayrutdinov BI, Lee C-K et al. Solution structure of the Zbeta domain of human DNA-dependent activator of IFN-regulatory factors and its binding modes to B- and Z-DNAs. Proc Natl Acad Sci USA. 2011;108:6921–6. 10.1073/pnas.1014898107. 21471454 PMC3084098

[10] Kim D, Hur J, Park K et al. Distinct Z-DNA binding mode of a PKR-like protein kinase containing a Z-DNA binding domain (PKZ). Nucleic Acids Res. 2014;42:5937–48. 10.1093/nar/gku189. 24682817 PMC4027156

[11] Lee J, Kim YG, Kim KK et al. Transition between B-DNA and Z-DNA: free energy landscape for the B-Z junction propagation. J Phys Chem B. 2010;114:9872–81. 10.1021/jp103419t. 20666528

[12] Mahmoud M, Volodymyr B, Christopher R et al. Reaction path ensemble of the B–Z-DNA transition: a comprehensive atomistic study. Nucleic Acids Res. 2013;41:33–43. 23104380 10.1093/nar/gks1003 PMC3592462

[13] Yi J, Yeou S, Lee NK. DNA bending force facilitates Z-DNA formation under physiological salt conditions. J Am Chem Soc. 2022;144:13137–45. 10.1021/jacs.2c02466. 35839423 PMC9335521

[14] Ray BK, Dhar S, Shakya A et al. Z-DNA-forming silencer in the first exon regulates human ADAM-12 gene expression. Proc Natl Acad Sci USA. 2011;108:103–8. 10.1073/pnas.1008831108. 21173277 PMC3017174

[15] Rothenburg S, Koch-Nolte F, Rich A et al. A polymorphic dinucleotide repeat in the rat nucleolin gene forms Z-DNA and inhibits promoter activity. Proc Natl Acad Sci USA. 2001;98:8985–90. 10.1073/pnas.121176998. 11447254 PMC55360

[16] Georgakopoulos-Soares I, Victorino J, Parada GE et al. High-throughput characterization of the role of non-B DNA motifs on promoter function. Cell Genomics. 2022;2:100111. 10.1016/j.xgen.2022.100111. 35573091 PMC9105345

[17] Gagna CE, Lambert WC, Kuo HRR et al. Localization of B-DNA and Z-DNA in terminally differentiating fiber cells in the adult lens. J Histochem Cytochem. 1997;45:1511–21. 10.1177/002215549704501108. 9358853

[18] Sinclair PB, Parker H, An Q et al. Analysis of a breakpoint cluster reveals insight into the mechanism of intrachromosomal amplification in a lymphoid malignancy. Hum Mol Genet. 2011;20:2591–602. 10.1093/hmg/ddr159. 21487021

[19] Wang G, Vasquez KM. Non-B DNA structure-induced genetic instability. Mutat Res. 2006;598:103–19. 16516932 10.1016/j.mrfmmm.2006.01.019

[20] Meng Y, Wang G, He H et al. Z-DNA is remodelled by ZBTB43 in prospermatogonia to safeguard the germline genome and epigenome. Nat Cell Biol. 2022;24:1141–53. 10.1038/s41556-022-00941-9. 35787683 PMC9276527

[21] Takaoka A, Wang Z, Choi MK et al. DAI (DLM-1/ZBP1) is a cytosolic DNA sensor and an activator of innate immune response. Nature. 2007;448:501–5. 10.1038/nature06013. 17618271

[22] Ravichandran S, Vinod Kumar S, Kyeong Kyu K. Z-DNA in the genome: from structure to disease. Biophys Rev. 2019;11:383–7. 10.1007/s12551-019-00534-1. 31119604 PMC6557933

[23] Umerenkov D, Herbert A, Konovalov D et al. Z-flipon variants reveal the many roles of Z-DNA and Z-RNA in health and disease. Life Sci Alliance. 2023;6:e202301962. 10.26508/lsa.202301962. 37164635 PMC10172764

[24] Fuertes MA, Cepeda V, Alonso C et al. Molecular mechanisms for the B−Z Transition in the example of poly[d(G−C)·d(G−C)] polymers. A critical review. Chem Rev. 2006;106:2045–64. 10.1021/cr050243f. 16771442

[25] Kim KK, Subramani VK. Z-DNA: Methods and protocols. New York, NY, Springer US, 2023.

[26] Sela I, Wolf YI, Koonin EV. Theory of prokaryotic genome evolution. Proc Natl Acad Sci USA. 2016;113:11399–407. 10.1073/pnas.1614083113. 27702904 PMC5068321

[27] Chow MH, Yan KTH, Bennett MJ et al. Birefringence and DNA condensation of liquid crystalline chromosomes. Euk Cell. 2010;9:1577–87. 10.1128/EC.00026-10. PMC2950428 20400466

[28] Marenduzzo D, Micheletti C, Orlandini E et al. Topological friction strongly affects viral DNA ejection. Proc Natl Acad Sci USA. 2013;110:20081–6. 10.1073/pnas.1306601110. 24272939 PMC3864349

[29] Zhao H, Speir JA, Matsui T et al. Structure of a bacterial virus DNA-injection protein complex reveals a decameric assembly with a constricted molecular channel. PLoS One. 2016;11:e0149337. 10.1371/journal.pone.0149337. 26882199 PMC4755594

[30] Vijayanathan V, Thomas T, Shirahata A et al. DNA condensation by polyamines: a laser light scattering study of structural effects. Biochemistry. 2001;40:13644–51. 10.1021/bi010993t. 11695913

[31] Matulis D, Rouzina I, Bloomfield VA. Thermodynamics of DNA binding and condensation: isothermal titration calorimetry and electrostatic mechanism. J Mol Biol. 2000;296:1053–63. 10.1006/jmbi.1999.3470. 10686103

[32] Manning GS . Self-attraction and natural curvature in null DNA. J Biomol Struct Dyn. 1989;7:41–61. 10.1080/07391102.1989.10507751. 2684222

[33] Manning GS . Thermodynamic stability theory for DNA doughnut shapes induced by charge neutralization. Biopolymers. 1980;19:37–59. 10.1002/bip.1980.360190104. 7370395

[34] Manning GS . Packaged DNA. An elastic model. Cell Biophys. 1985;7:57–89. 10.1007/BF02788639. 2408756

[35] Guéron M, Demaret J, Filoche M. A unified theory of the B–Z transition of DNA in high and low concentrations of multivalent ions. Biophysical J. 2000;78:1070–83. 10.1016/s0006-3495(00)76665-3 PMC1300710 10653820

[36] Bae S, Son H, Kim Y-G et al. Z-DNA stabilization is dominated by the Hofmeister effect. Phys Chem Chem Phys. 2013;15:15829–32. 10.1039/c3cp52047a. 23995025

[37] Pan F, Roland C, Sagui C. Ion distributions around left- and right-handed DNA and RNA duplexes: a comparative study. Nucleic Acids Res. 2014;42:13981–96. 10.1093/nar/gku1107. 25428372 PMC4267617

[38] Bhattacharyya D, Mirihana Arachchilage G, Basu S. Metal cations in G-quadruplex folding and stability. Front Chem. 2016;4. 10.3389/fchem.2016.00038. PMC5016522 27668212

[39] Van de Sande JH, Jovin TM. Z* DNA, the left-handed helical form of poly[d(G-C)] in MgCl2-ethanol, is biologically active. EMBO J. 1982;1:115–20. 10.1002/j.1460-2075.1982.tb01133.x. 6232131 PMC553004

[40] Revet B, Delain E, Dante R et al. Three dimensional association of double-stranded helices are produced in conditions for Z-DNA formation. J Biomol Struct Dyn. 1983;1:857–71. 10.1080/07391102.1983.10507489. 6400905

[41] Castleman H, Specthrie L, Makowski L et al. Electronmicroscopy and circular dichroism of the dynamics of the formation and dissolution of supramolecular forms of Z-DNA. J Biomol Struct Dyn. 1984;2:271–83. 10.1080/07391102.1984.10507566. 6400936

[42] Castleman H, Erlanger BF. Electron microscopy of “Z-DNA.”. Cold Spring Harbor Symp Quant Biol. 1983;47–133–42. 10.1101/SQB.1983.047.01.018. 6574839

[43] Thomas TJ, Bloomfield VA. Toroidal condensation of Z DNA and identification of an intermediate in the B to Z transition of poly(dG-m5dC) X poly(dG-m5dC). Biochemistry. 1985;24:713–9. 10.1021/bi00324a026. 3994981

[44] Ma C, Sun L, Bloomfield VA. Condensation of plasmids enhanced by Z-DNA conformation of d(CG)n inserts. Biochemistry. 1995;34:3521–8. 10.1021/bi00011a005. 7893647

[45] Reich Z, Ghirlando R, Minsky A. Secondary conformational polymorphism of nucleic acids as a possible functional link between cellular parameters and DNA packaging processes. Biochemistry. 1991;30:7828–36. 10.1021/bi00245a024. 1868059

[46] Reich Z, Friedman P, Levin-Zaidman S et al. Effects of adenine tracts on the B-Z transition. Fine tuning of DNA conformational transition processes. J Biol Chem. 1993;268:8261–6. 10.1016/S0021-9258(18)53091-X. 8463336

[47] Sitko JC, Mateescu EM, Hansma HG. Sequence-dependent DNA condensation and the electrostatic zipper. Biophys J. 2003;84:419–31. 10.1016/S0006-3495(03)74862-0. 12524295 PMC1302623

[48] Patil SD, Rhodes DG, Burgess DJ. Biophysical characterization of anionic lipoplexes. Biochim Biophys Acta. 2005;1711:1–11. 10.1016/j.bbamem.2005.03.004. 15904657

[49] Zacharias W, Martin JC, Wells RD. A condensed form of (dG-dC)n.cntdot.(dG-dC)n as an intermediate between the B- and Z- conformations induced by sodium acetate. Biochemistry. 1983;22:2398–405. 10.1021/bi00279a015. 6860635

[50] Hasan R, Alam MK, Ali R. Polyamine induced Z-conformation of native calf thymus DNA. FEBS Lett. 1995;368:27–30. 10.1016/0014-5793(95)00591-V. 7615082

[51] Choi JK, Sargsyan G, Shabbir-Hussain M et al. Chiroptical detection of condensed nickel(II)-Z-DNA in the presence of the B-DNA via porphyrin exciton coupled circular dichroism. J Phys Chem B. 2011;115:10182–8. 10.1021/jp2047213. 21774503 PMC3177531

[52] Chaires JB, Norcum MT. Structure and stability of Z* DNA. J Biomol Struct Dyn. 1988;5:1187–207. 10.1080/07391102.1988.10506463. 3271507

[53] Jovin TM, McIntosh LP, Arndt-Jovin DJ et al. Left-handed DNA: from synthetic polymers to chromosomes. J Biomol Struct Dyn. 1983;1:21–57. 10.1080/07391102.1983.10507425. 6401113

[54] Jovin TM . In: Kim K. K., Subramani V. K. (eds.), Z-DNA: Methods and protocols. New York, NY, Springer US, 2023;pp.1–32.

[55] Herbert A . The simple biology of flipons and condensates enhances the evolution of complexity. Molecules. 2021;26:4881. 10.3390/molecules26164881. 34443469 PMC8400190

[56] Arndt-Jovin DJ, Robert-Nicoud M, Zarling DA et al. Left-handed Z-DNA in bands of acid-fixed polytene chromosomes. Proc Natl Acad Sci USA. 1983;80:4344–8. 10.1073/pnas.80.14.4344. 6410390 PMC384034

[57] Soyer-Gobillard MO, Géraud ML, Coulaud D et al. Location of B- and Z-DNA in the chromosomes of a primitive eukaryote dinoflagellate. J Cell Biol. 1990;111:293–304. 10.1083/jcb.111.2.293. 2380241 PMC2116181

[58] Thomas TJ, Thomas T. Direct evidence for the presence of left-handed conformation in a supramolecular assembly of polynucleotides. Nucl Acids Res. 1989;17:3795–810. 10.1093/nar/17.10.3795. 2660102 PMC317860

[59] Li W, Nordenskiöld L, Zhou R et al. Conformation-dependent DNA attraction. Nanoscale. 2014;6:7085–92. 10.1039/C3NR03235C. 24847505

[60] Schwartz T, Shafer K, Lowenhaupt K et al. Crystallization and preliminary studies of the DNA-binding domain Za from ADAR1 complexed to left-handed DNA. Acta Crystallogr D Biol Crystallogr. 1999;55:1362–4. 10.1107/S090744499900582X. 10393308

[61] Kim D, Hwang H-Y, Kim YG et al. Crystallization and preliminary X-ray crystallographic studies of the Z-DNA-binding domain of a PKR-like kinase (PKZ) in complex with Z-DNA. Acta Crystallogr F Struct Biol Cryst Commun. 2009;65:267–70. 10.1107/S1744309109002504. PMC2650443 19255480

[62] Shechter D, Dormann HL, Allis CD et al. Extraction, purification and analysis of histones. Nat Protoc. 2007;2:1445–57. 10.1038/nprot.2007.202. 17545981

[63] Kim KK, Subramani VK. In: Subramani V. K., Kim K. K. (eds.), Z-DNA: Methods and protocols. 2023;Vol. 2651:pp.33–51.

[64] Schwartz T, Rould MA, Lowenhaupt K et al. Crystal structure of the Zalpha domain of the human editing enzyme ADAR1 bound to left-handed Z-DNA. Science. 1999;284:1841–5. 10.1126/science.284.5421.1841. 10364558

[65] Kang H, Yoo J, Sohn B-K et al. Sequence-dependent DNA condensation as a driving force of DNA phase separation. Nucleic Acids Res. 2018;46:9401–13. 10.1093/nar/gky639. 30032232 PMC6182145

[66] Taton TA . Preparation of gold nanoparticle–DNA conjugates. Curr Protoc Nucleic Acid Chem. 2002;9:12.2.1–12.2.12. 10.1002/0471142700.nc1202s09 18428889

[67] Wang J, Wu L, Ren J et al. Visualizing human telomerase activity with primer-modified Au nanoparticles. Small. 2012;8:259–64. 10.1002/smll.201101938. 22083963

[68] Subramani VK, Kim D, Yun K et al. Structural and functional studies of a large winged Z-DNA-binding domain of *Danio rerio* protein kinase PKZ. FEBS Lett. 2016;590:2275–85. 10.1002/1873-3468.12238. 27265117

[69] Bao H-L, Masuzawa T, Oyoshi T et al. Oligonucleotides DNA containing 8-trifluoromethyl-2′-deoxyguanosine for observing Z-DNA structure. Nucleic Acids Res. 2020;48:7041–51. 32678885 10.1093/nar/gkaa505 PMC7367190

[70] Schindelin J, Arganda-Carreras I, Frise E et al. Fiji: an open-source platform for biological-image analysis. Nat Methods. 2012;9:676–82. 10.1038/nmeth.2019. 22743772 PMC3855844

[71] Chen S, Zhou Y, Chen Y et al. fastp: an ultra-fast all-in-one FASTQ preprocessor. Bioinformatics. 2018;34:i884–90.- 10.1093/bioinformatics/bty560i890. 30423086 PMC6129281

[72] Langmead B, Salzberg SL. Fast gapped-read alignment with Bowtie 2. Nat Methods. 2012;9:357–9. 10.1038/nmeth.1923. 22388286 PMC3322381

[73] Amemiya HM, Kundaje A, Boyle AP. The ENCODE Blacklist: identification of problematic regions of the genome. Sci Rep. 2019;9:9354. 10.1038/s41598-019-45839-z. 31249361 PMC6597582

[74] Danecek P, Bonfield JK, Liddle J et al. Twelve years of SAMtools and BCFtools. Gigascience. 2021;10:10.1093/gigascience/giab008. PMC7931819 33590861

[75] Zhang Y, Liu T, Meyer CA et al. Model-based analysis of ChIP-Seq (MACS). Genome Biol. 2008;9:R137. 10.1186/gb-2008-9-9-r137. 18798982 PMC2592715

[76] Li S, Olson WK, Lu XJ. Web 3DNA 2.0 for the analysis, visualization, and modeling of 3D nucleic acid structures. Nucleic Acids Res. 2019;47:W26–34. 10.1093/nar/gkz394. 31114927 PMC6602438

[77] Van Der Spoel D, Lindahl E, Hess B et al. GROMACS: fast, flexible, and free. J Comput Chem. 2005;26:1701–18. 10.1002/jcc.20291. 16211538

[78] Perez A, Marchan I, Svozil D et al. Refinement of the AMBER force field for nucleic acids: improving the description of alpha/gamma conformers. Biophys J. 2007;92:3817–29. 10.1529/biophysj.106.097782. 17351000 PMC1868997

[79] Joung IS, Cheatham TE., 3rd. Determination of alkali and halide monovalent ion parameters for use in explicitly solvated biomolecular simulations. J Phys Chem B. 2008;112:9020–41. 10.1021/jp8001614. 18593145 PMC2652252

[80] Jorgensen WL, Chandrasekhar J, Madura JD et al. Comparison of simple potential functions for simulating liquid water. J Chem Phys. 1983;79:926–35. 10.1063/1.445869.

[81] Yoo J, Aksimentiev A. Improved parametrization of Li+, Na+, K+, and Mg2+ ions for all-atom molecular dynamics simulations of nucleic acid systems. J Phys Chem Lett. 2012;3:45–50. 10.1021/jz201501a.

[82] Yoo J, Aksimentiev A. Improved parameterization of amine-carboxylate and amine-phosphate interactions for molecular dynamics simulations using the CHARMM and AMBER force fields. J Chem Theory Comput. 2016;12:430–43. 10.1021/acs.jctc.5b00967. 26632962

[83] Valdes-Tresanco MS, Valdes-Tresanco ME, Valiente PA et al. gmx_MMPBSA: a new tool to perform end-state free energy calculations with GROMACS. J Chem Theory Comput. 2021;17:6281–91. 10.1021/acs.jctc.1c00645. 34586825

[84] Tan C, Tan YH, Luo R. Implicit nonpolar solvent models. J Phys Chem B. 2007;111:12263–74. 10.1021/jp073399n. 17918880

[85] Lu Q, Luo R. A Poisson–Boltzmann dynamics method with nonperiodic boundary condition. J Chem Phys. 2003;119:11035–47. 10.1063/1.1622376.

[86] Meng EC, Goddard TD, Pettersen EF et al. UCSF ChimeraX: tools for structure building and analysis. Protein Sci. 2023;32:e4792. 10.1002/pro.4792. 37774136 PMC10588335

[87] Deng Z, Tian Y, Lee S-H et al. DNA-encoded self-assembly of gold nanoparticles into one-dimensional arrays. Angew Chem Int Ed. 2005;44:3582–5. 10.1002/anie.200463096. 15880749

[88] Hurst SJ, Hill H, Fau - Mirkin CA et al. “Three-dimensional hybridization” with polyvalent DNA-gold nanoparticle conjugates. J Am Chem Soc. 2008; 130:12192–200. 10.1021/ja804266j. 18710229 PMC8191498

[89] Sato K, Hosokawa K, Maeda M. Rapid aggregation of gold nanoparticles induced by non-cross-linking DNA hybridization. J Am Chem Soc. 2003;125:8102–3. 10.1021/ja034876s. 12837070

[90] Zhang H, Yu H, Ren J et al. Reversible B/Z-DNA transition under the low salt condition and non-B-form polydApolydT selectivity by a cubane-like europium-L-aspartic acid complex. Biophys J. 2006;90:3203–7. 10.1529/biophysj.105.078402. 16473901 PMC1432110

[91] Behe M, Felsenfeld G. Effects of methylation on a synthetic polynucleotide: the B-Z transition in poly(dG-m5dC).poly(dG-m5dC). Proc Natl Acad Sci USA. 1981;78:1619–23. 10.1073/pnas.78.3.1619. 6262820 PMC319183

[92] Springer T, Ermini ML, Spackova B et al. Enhancing sensitivity of surface plasmon resonance biosensors by functionalized gold nanoparticles: size matters. Anal Chem. 2014;86:10350–6. 10.1021/ac502637u. 25226207

[93] Snopok BA, Nizamov SN, Snopok TV et al. Agglomeration compaction promotes corrosion of gold nanoparticles. Nanoscale Adv. 2024;6:3865–77. 10.1039/D4NA00109E. 39050952 PMC11265584

[94] Basu S, Ghosh SK, Kundu S et al. Biomolecule induced nanoparticle aggregation: effect of particle size on interparticle coupling. J Colloid Interface Sci. 2007;313:724–34. 10.1016/j.jcis.2007.04.069. 17540397

[95] Wang Y, Quinsaat JEQ, Ono T et al. Enhanced dispersion stability of gold nanoparticles by the physisorption of cyclic poly(ethylene glycol). Nat Commun. 2020;11:6089. 10.1038/s41467-020-19947-8. 33257670 PMC7705015

[96] Subramani VK, Ravichandran S, Bansal V et al. Chemical-induced formation of BZ-junction with base extrusion. Biochem Biophys Res Commun. 2019;508:1215–20. 10.1016/j.bbrc.2018.12.045. 30558789

[97] Zhang T, Yin C, Fedorov A et al. ADAR1 masks the cancer immunotherapeutic promise of ZBP1-driven necroptosis. Nature. 2022;606:594–602. 10.1038/s41586-022-04753-7. 35614224 PMC9373927

[98] Ellison MJ, Feigon J, Kelleher RJ et al. An assessment of the Z-DNA forming potential of alternating dA-dT stretches in supercoiled plasmids. Biochemistry. 1986;25:3648–55. 10.1021/bi00360a026. 3718951

[99] McLean MJ, Wells RD, Kilpatrick MW et al. Consecutive A X T pairs can adopt a left-handed DNA structure. Proc Natl Acad Sci USA. 1986;83:5884–8. 10.1073/pnas.83.16.5884. 3016726 PMC386401

[100] Klysik J, Zacharias W, Galazka G et al. Structural interconversion of alternating purine-pyrimidine inverted repeats cloned in supercoiled plasmids. Nucl Acids Res. 1988;16:6915–33. 10.1093/nar/16.14.6915. 3405754 PMC338342

[101] Beknazarov N, Jin S, Poptsova M. Deep learning approach for predicting functional Z-DNA regions using omics data. Sci Rep. 2020;10:19134. 10.1038/s41598-020-76203-1. 33154517 PMC7644757

[102] Beknazarov N, Poptsova M. In: Kim K. K., Subramani V. K. (eds.), Z-DNA: Methods and protocols, New York, NY: Humana 2023, Vol 2651, pp.217–26.

[103] Nordheim A, Lafer EM, Peck LJ et al. Negatively supercoiled plasmids contain left-handed Z-DNA segments as detected by specific antibody binding. Cell. 1982;31:309–18. 10.1016/0092-8674(82)90124-6. 7159926

[104] Shin S-I, Ham S, Park J et al. Z-DNA-forming sites identified by ChIP-Seq are associated with actively transcribed regions in the human genome. DNA Res. 2016;23:477–86. 10.1093/dnares/dsw031. 27374614 PMC5066173

[105] Liang D, Yuguang M, Lars N et al. Molecular dynamics simulation of multivalent-ion mediated attraction between DNA molecules. Phys Rev Lett. 2008;100:118301. 18517834 10.1103/PhysRevLett.100.118301

[106] Luan B, Aksimentiev A. DNA attraction in monovalent and divalent electrolytes. J Am Chem Soc. 2008;130:15754–5. 10.1021/ja804802u. 18975864 PMC2903614

[107] Chatake T, Hikima T, Ono A et al. Crystallographic studies on damaged DNAs. II. N(6)-methoxyadenine can present two alternate faces for Watson-Crick base-pairing, leading to pyrimidine transition mutagenesis. J Mol Biol. 1999;294:1223–30. 10.1006/jmbi.1999.3304. 10600380

[108] Egli M, Williams LD, Gao Q et al. Structure of the pure-spermine form of Z-DNA (magnesium free) at 1-A resolution. Biochemistry. 1991;30:11388–402. 10.1021/bi00112a005. 1742278

[109] Teif VB, Bohinc K. Condensed DNA: condensing the concepts. Prog Biophys Mol Biol. 2011;105:208–22. 10.1016/j.pbiomolbio.2010.07.002. 20638406

[110] Victor AB . DNA condensation by multivalent cations. Biopolymers. 1997;44:269–82. 9591479 10.1002/(SICI)1097-0282(1997)44:3<269::AID-BIP6>3.0.CO;2-T

[111] Bloomfield VA, Crothers DM, Tinoco I et al. Nucleic acids: Structures, properties, and functions. 1 edition ed. University Science Books, Sausalito, California, University Science Books 2000.

[112] Demers LM, Mucic RC, Reynolds RA et al. A fluorescence-based method for determining the surface coverage and hybridization efficiency of thiol-capped oligonucleotides bound to gold thin films and nanoparticles. Anal Chem. 2000;72:5535–41. 10.1021/ac0006627. 11101228

[113] Wang AHJ, Quigley GJ, Kolpak FJ et al. Molecular structure of a left-handed double helical DNA fragment at atomic resolution. Nature. 1979;282:680–6. 10.1038/282680a0. 514347

[114] Egli M . DNA-cation interactions: quo vadis?. Chem Biol. 2002;9:277–86. 10.1016/S1074-5521(02)00116-3. 11927253

[115] Shamim A, Parveen N, Subramani VK et al. Molecular packing interaction in DNA crystals. Cryst. 2020;10:1093. 10.3390/cryst10121093.

[116] Raspaud E, Olvera de la Cruz M, Sikorav JL et al. Precipitation of DNA by polyamines: a polyelectrolyte behavior. Biophys J. 1998;74:381–93. 10.1016/S0006-3495(98)77795-1. 9449338 PMC1299390

[117] Yoo J, Aksimentiev A. The structure and intermolecular forces of DNA condensates. Nucleic Acids Res. 2016;44:2036–46. 10.1093/nar/gkw081. 26883635 PMC4797306

[118] Alexander R, Shuguang Z. Z-DNA: the long road to biological function. Nat Rev Genet. 2003;4:566–72. 12838348 10.1038/nrg1115

[119] Preisler RS, Chen HH, Colombo MF et al. The B form to Z form transition of poly(dG-m5dC) is sensitive to neutral solutes through an osmotic stress. Biochemistry. 1995;34:14400–7. 10.1021/bi00044a017. 7578044

[120] Peck LJ, Wang JC. Transcriptional block caused by a negative supercoiling induced structural change in an alternating CG sequence. Cell. 1985;40:129–37. 10.1016/0092-8674(85)90316-2. 2981624

[121] Moller A, Nordheim A, Patel DJ et al. Bromination stabilizes poly(dG-dC) in the Z-DNA form under low-salt conditions. Biochemistry. 1984;23:54–62. 10.1021/bi00296a009. 6691966

[122] Thomas TJ . Polyamine-induced Z-DNA conformation in plasmids containing (dA-dC)n.(dG-dT)n inserts and increased binding of lupus autoantibodies to the Z-DNA form of plasmids. Biochemical J. 1994;298–485–91. 10.1042/bj2980485. PMC1137966 8135759

[123] D’Urso A, Mammana A, Balaz M et al. Interactions of a tetraanionic porphyrin with DNA: from a Z-DNA sensor to a versatile supramolecular device. J Am Chem Soc. 2009;131:2046–7. 19159291 10.1021/ja808099u

[124] Coburn RA, Baker PJ, Evans RT et al. *In vitro* antiplaque properties of a series of alkyl bis(biguanides). J Med Chem. 1978;21:828–9. 10.1021/jm00206a024. 691009

[125] Kasikci E, Aydemir E, Yogurtcu BM et al. Repurposing of alexidine dihydrochloride as an apoptosis initiator and cell cycle inhibitor in human pancreatic cancer. ACAMC. 2020;20:1956–65. 10.2174/1871520620666200508085439. 32384037

[126] Li M, Wang Y, Li X et al. Pharmacological targeting of the mitochondrial phosphatase PTPMT1 sensitizes hepatocellular carcinoma to ferroptosis. Cell Death Dis. 2025;16:257. 10.1038/s41419-025-07581-5. 40189563 PMC11973169

