# Supplementary material for: Alexidine, identified as a Z-DNA inducer by the NanoZ screening platform, acts as a transcriptional regulator
Source: Nucleic Acids Res. 2026 Apr 8;54(6):gkag281. doi: 10.1093/nar/gkag281 (PMC13069682; doi:10.1093/nar/gkag281)
Supplement: gkag281_Supplemental_Files [file gkag281_supplemental_files.zip › Alexidine-Sup-revision2-f_3.docx]

**Supplementary Materials**

**Alexidine, identified as a Z-DNA inducer by the NanoZ screening platform, acts as a transcriptional regulator**

Vinod Kumar Subramani^1,†*^, Shrute Kannappan ^1, 2,†^, Shiyu Wang^3^, Yan Xu^3^, Subramaniyam Ravichandran^1^, Boi Hoa San^1^, Jung Heon Lee^2,4*^, and Kyeong Kyu Kim^1*^

^1^Department of Precision Medicine, Institute for Antimicrobial Resistance Research and Therapeutics, Sungkyunkwan University School of Medicine, Suwon 16419, Korea

^2^Research Center for Advanced Materials Technology, Core Research Institute, Suwon, 16419, Korea

^3^Division of Chemistry, Department of Medical Sciences, Faculty of Medicine, University of Miyazaki, Kiyotake, Japan.

^4^Department of Advanced Material Science and Engineering, Sungkyunkwan University, Suwon 16419, Korea.

^†^ The first two authors should be regarded as Joint First Authors

*To whom all the correspondence should be addressed: [kyeongkyu@skku.edu](mailto:kyeongkyu@skku.edu), [jhlee7@skku.edu](mailto:jhlee7@skku.edu), or [mailvinod@skku.edu](mailto:mailvinod@skku.edu) *

**Table S1. List of primers used for qRT-PCR analysis.**

| **Gene** | **Forward primer** | **Reverse primer** |
| --- | --- | --- |
| m-*Smyd2* | \| AAGGATTGTCAAAATGTGGACGG \| \| --- \| | ATGGAGGAGCATTCCAGCTTG |
| m-*Sfi1* | TTGGGGAGCAGCAGTTAGAGA | \| CGGACCAGGAACATTCGGC \| \| --- \| |
| m-*Smg6* | \| AACAAACCGGATAAAACCGAAT \| \| --- \| | \| CGGTAGCGATTCCTTCTCTTG \| \| --- \| |
| m-*Gtpbp4* | ATCACTACAAGTTGGCTCTAGGT | \| GCATCGGTACAGAGAATCACCAT \| \| --- \| |
| m-*Adcy8* | CAGCTACCGAGGGGTCATTTT | \| ACGTTCATCACTACCTCCGATT \| \| --- \| |
| m-*Pdcd4* | CCACTGACCCTGACAATTTAAGC | \| TTTTCCGCAGTCGTCTTTTGG \| \| --- \| |
| m-*Cdk6* | \| GGCGTACCCACAGAAACCATA \| \| --- \| | AGGTAAGGGCCATCTGAAAACT |
| m-*Efcab3* | ATTTGCGAGGATCTTTCTCAGAC | \| TGCTTATCAATCCGTGTGAATCA \| \| --- \| |
| m-*Atp6v1h* | \| GGATGCTGCTGTCCCAACTAA \| \| --- \| | \| TCTCTTGCTTGTCCTCGGAAC \| \| --- \| |
| m-*Ankib1* | \| ACAACCACCAAATTCCGCAAA \| \| --- \| | \| TGCTGATAGGGTTCTCCATAAGA \| \| --- \| |
| m-*Rgs20* | \| GCTGCTGCACCTGTTCTTG \| \| --- \| | \| GCCCAGGCACAGACTTCTTC \| \| --- \| |
| m-*Nlgn4l* | ATGGCGTCCACTGACATCAC | CAGCGTAGGCTGAGTTAGGG |
| m-*Akap17a* | AAGTGACTGTGGGGAGACCG | CTTGGTGATCGGCTTCAGGT |


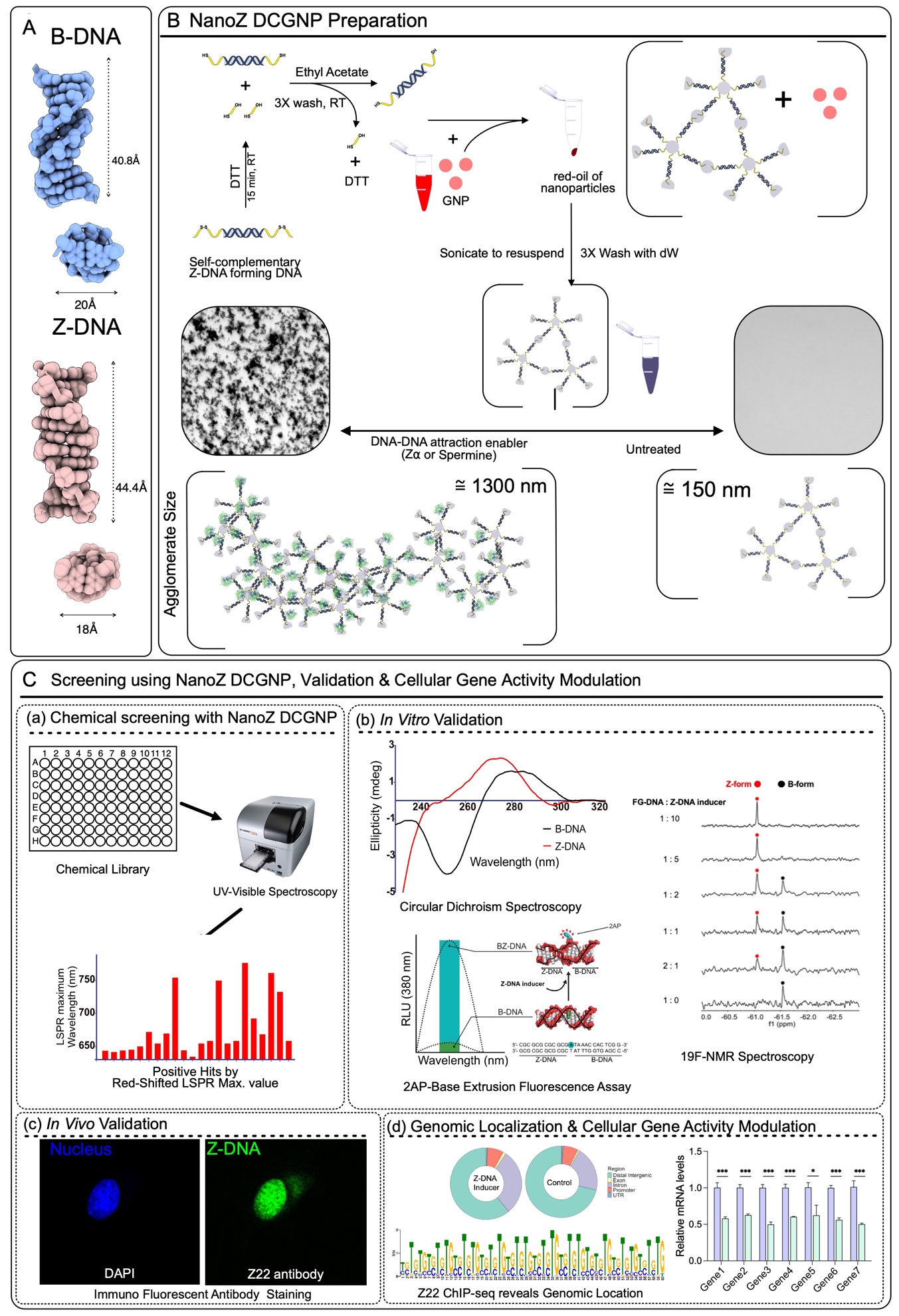


**Figure S1. Overview of experimental methods and validation process in the NanoZ platform. (A) Space-filling models of B-DNA (**PDB ID 1FQ2) **and Z-DNA** (PDB ID 4LB5)**. (B) Preparation of NanoZ DCGNP.** Thiolated DNA is first deprotected in the presence of DTT, followed by extraction with ethyl acetate. The DNA is then conjugated to colloidal gold nanoparticles to produce a red-oil suspension of nanoparticles, which are subsequently isolated and purified. The image illustrates the difference in agglomerate sizes when treated with Z⍺, which induces DNA condensation, compared to untreated NanoZ DCGNP. **(C) Screening, validation, and cellular gene activity modulation process. (a) Chemical screening with NanoZ DCGNP:** Potent DNA condensation inducers are identified from small-molecule chemical libraries. These inducers cause a red shift in the LSPR (extinction) maximum, detected using UV-Visible spectroscopy. **(b) *In vitro* validation:** Positive hits from the chemical screening are further validated using circular dichroism spectroscopy and the 2-aminopurine base extrusion fluorescence assay. **(c) *In vivo* validation:** Immunofluorescence staining of cultured animal cells is performed to detect the presence of Z-DNA in the presence of the identified Z-DNA inducers, providing confirmation of the *in vivo* effectiveness of the Z-DNA inducers. (d) Genomic localization and cellular gene activity modulation: Z22 based ChIP-seq of cells treated with Z-DNA inducer reveals enrichment of Z-DNA forming sites and their genomic location. The distribution of these sites across the genome is revealed, with emergent genes tested for activity modulation by the treated Z-DNA inducer.


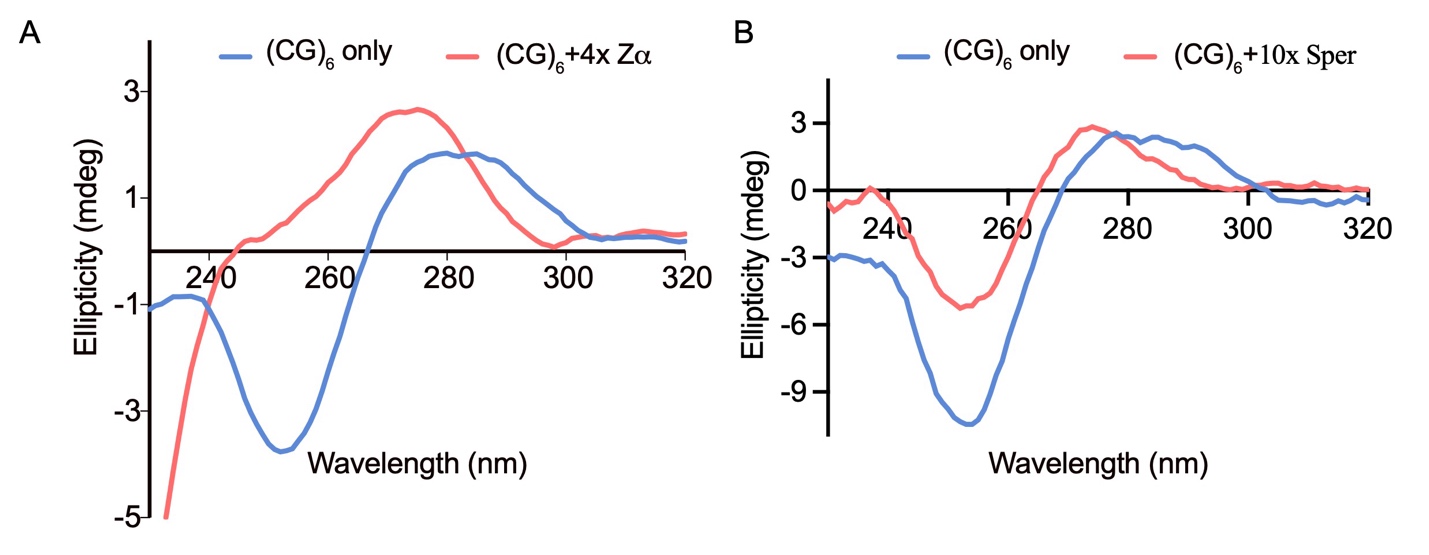


**Figure S2. B-to-Z transition by Z-DNA inducers.**

The circular dichroism (CD) spectrum of 10 µM (CG)₆, in the presence of 4-molar and 10-molar excess of Zα (A) and spermine (B). Labels: Z⍺ - Z⍺ domain from human ADAR1 protein.


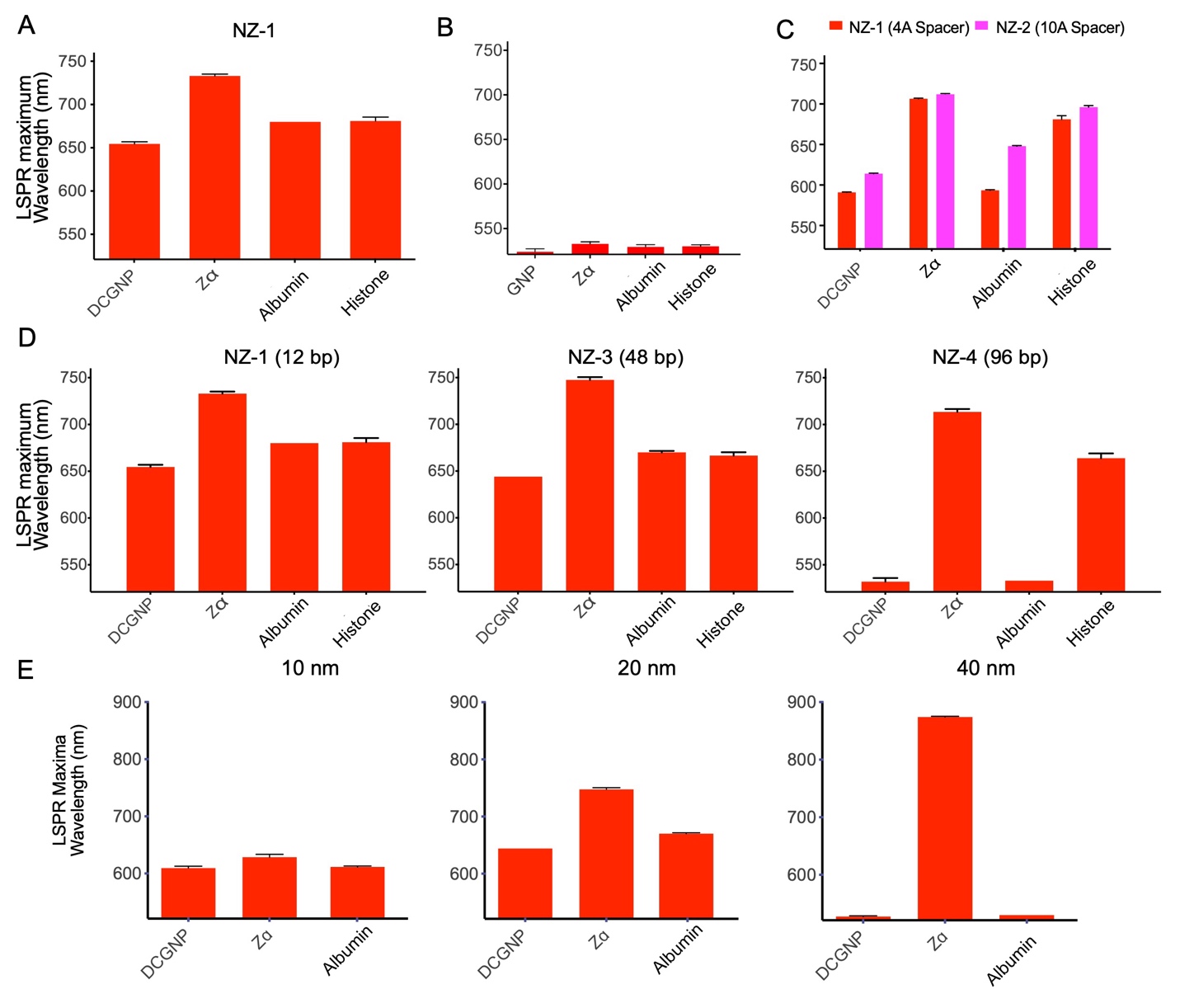


**Figure S3. Optimization of the NanoZ DCGNP for screening.**

**(A, B) The effects** Z⍺, bovine serum albumin and histone **on the** bathochromic shift **of** DCGNP with NZ-1 (A) and GNP (B). **(C) Effect of spacer length on sensor performance** **(D) Effect of ODN length** on sensor performance. (E) Effect of GNP size on sensor performance. Labels: LSPR - localized surface plasmon resonance; DCGNP - DNA conjugated with gold nanoparticle; GNP - gold nanoparticle; Zα - Zα domain from human ADAR1 protein.

**
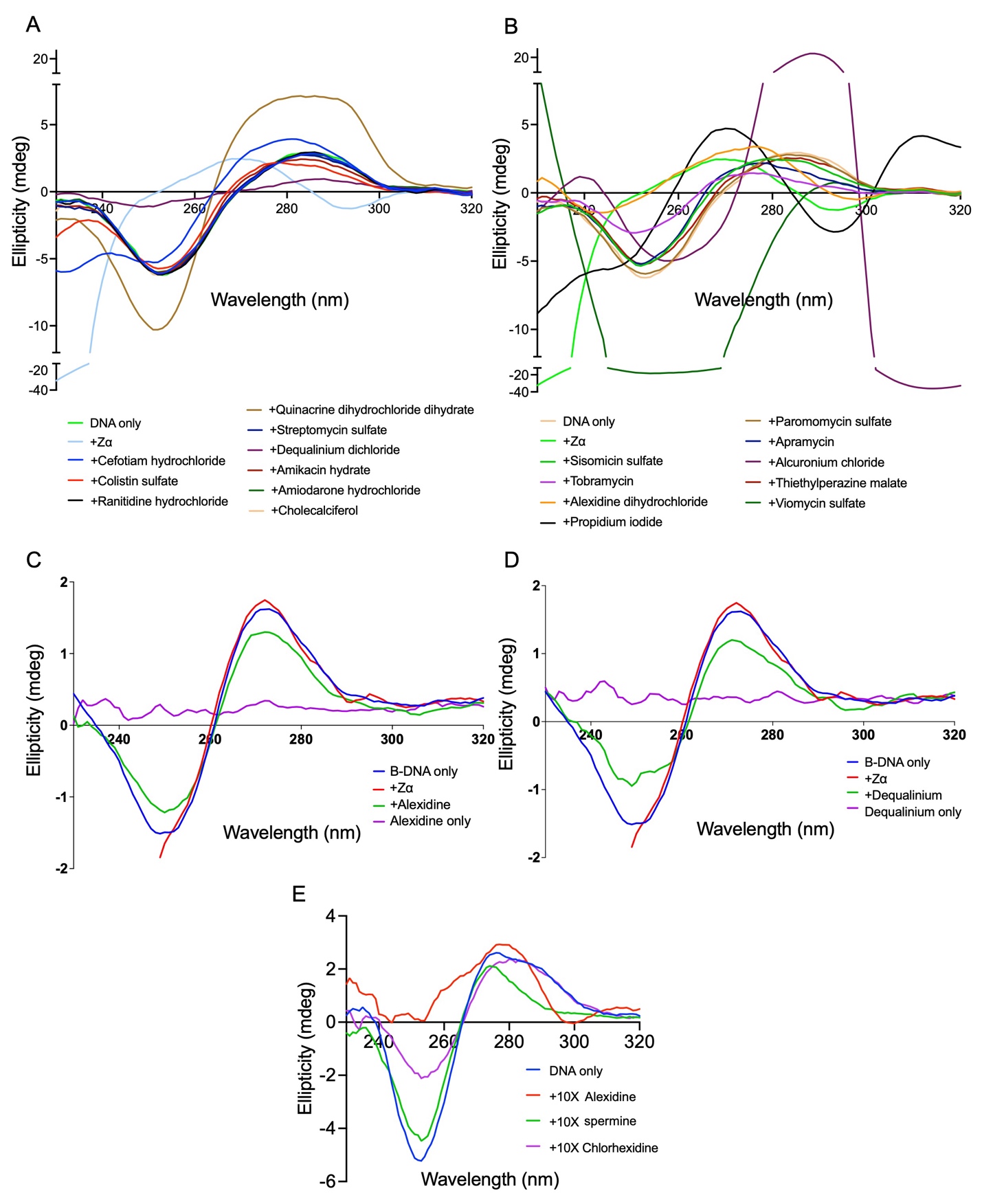
Figure S4. Circular dichroism (CD) spectra of (CG)_6_ upon treatment with identified DNA condensation inducers**.

(A, B) CD spectra of 15 µM (CG)_6_ in the presence of 150 µM (10 molar excess) of positive hits from NanoZ DCGNP screening experiments are shown. As a control, 60 µM Zα (4 molar excess) was used. (C, D) CD spectra of 15 µM (TA)_6_ in the presence of 150 µM Alexidine (C) and 150 µM Dequalinium (D), displaying the effect of the identified inducers on the B-DNA conformation. (E) CD spectrum investigation of BZ transition by CD spectroscopy in structurally related biguanide chlorhexidine. 15 µM of duplex (CG)_6_ DNA was incubated at 10 molar excess (150 µM) of the indicated small molecules

**
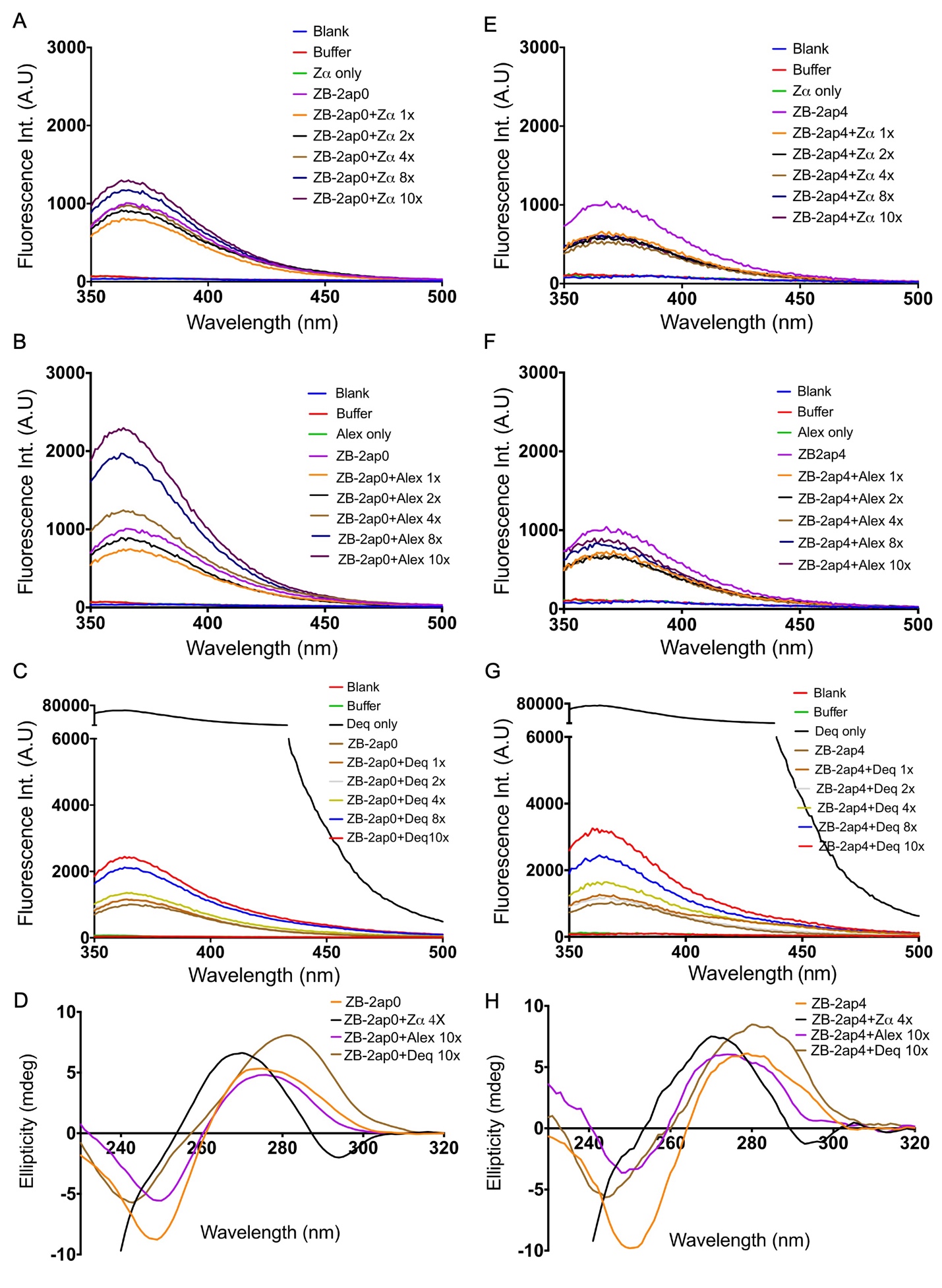
**

**Figure S5. 2-Aminopurine base extrusion fluorescence assay (2AP-BEFA) and circular dichroism (CD) spectroscopic analyses of the identified DNA condensation inducers.**

(A-C) The fluorescence emission spectrum was scanned after 320 nm excitation of 2AP in the probe ZB-2ap0, with increasing amounts of Zα (A), Alexidine (B), and Dequalinium (C), compared against the baseline from the probe only. (D) CD spectra for samples in the presence of candidate chemicals or Zα were used to test the chemicals' ability to induce the B-to-Z transition in ZB-2ap0. (E-G) Similar analyses were conducted with the ZB-2ap4 probe with increasing amounts of Zα (E), Alexidine (F), and Dequalinium (G). (H) CD spectra of candidate chemicals or Zα in ZB-2ap4. Labels: ZB-2ap0, BZ junction forming DNA labeled with 2AP at the base extrusion site; ZB-2ap4, BZ junction forming DNA labeled with 2AP at fourth base downstream of the extrusion site in the B-DNA forming region; Zα, Zα domain from human ADAR1; Blank, empty well; and Buffer, 5 mM HEPES pH 7.5, 10 mM NaCl.


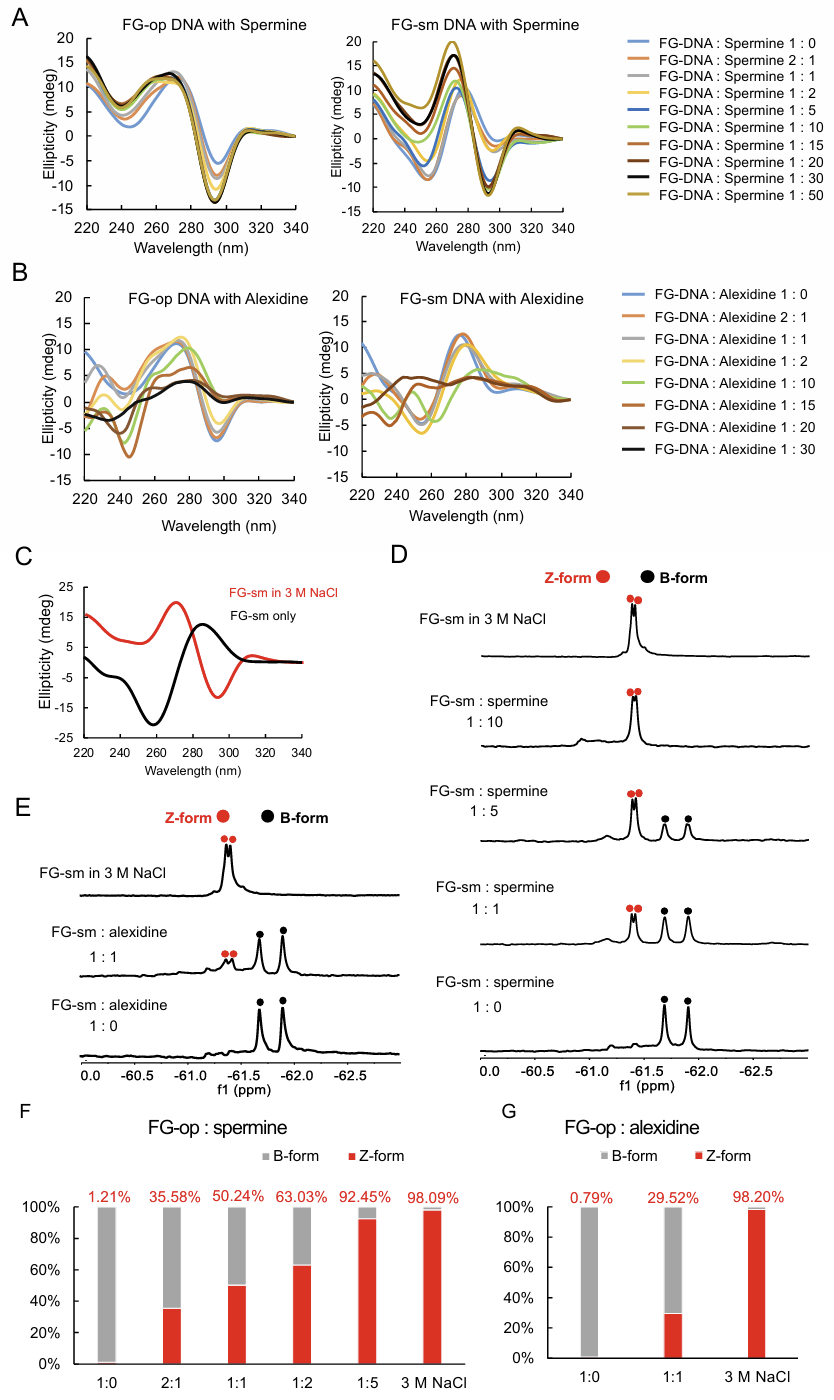


**Figure S6. Circular dichroism** (**CD) spectroscopy of 19F NMR DNA in the presence of Z-DNA inducers.**

CD spectra of both the FG-DNA were recorded in the presence of spermine (A) or Alexidine (B) at the indicated molar excess of the chemical. The plot of the left column indicates spectra of FG-op DNA, while the plot on the right column shows spectra of FG-sm DNA. FG-DNA was used at 10 µM, and the ratio indicates the molar excess of the respective chemicals used. (C) CD spectrum of the FG-sm DNA in the presence and absence of 3 M NaCl to show B-to-Z transition in this DNA. (D-E) ^19^F-NMR titration at the indicated molar excess of spermine (D) or Alexidine (E). FG-sm was used at 10 µM, and the ratio indicates the molar excess of the respective chemicals used. The reactions were buffered in a solution containing 10 mM NaCl, 1 mM Na-PO_4_ (pH 7.0) at 10℃. (F-G) Quantitative assay of ^19^F signal intensities of B- and Z-form DNA from ^19^F NMR spectroscopy during the investigation of B-Z transition of FG-op DNA in the presence of spermine (F), and Alexidine (G). The percentage values of Z form DNA are shown as red labels.


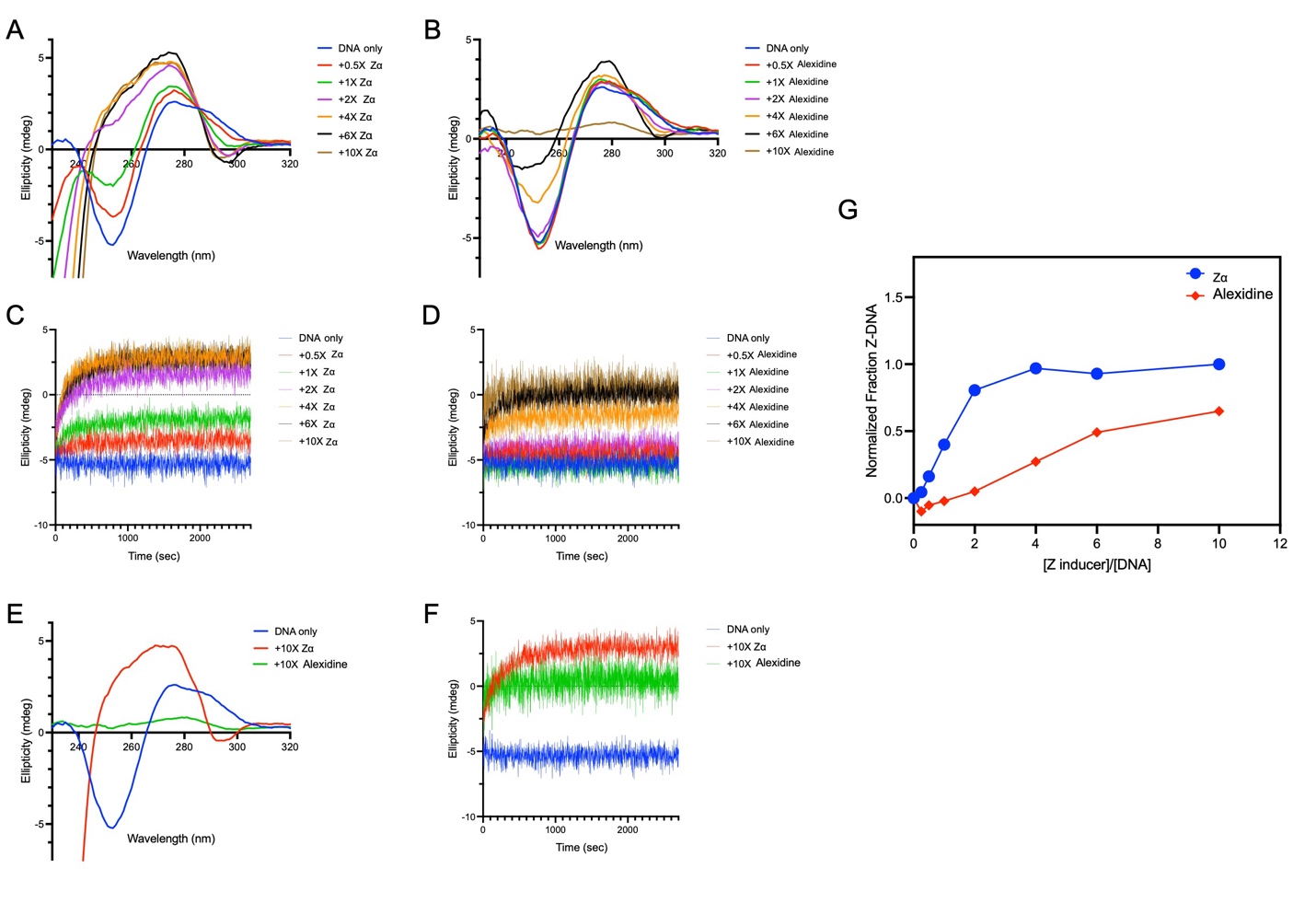


**Figure S7.** **Circular dichroism analysis of B-to-Z DNA transition in (CG)_6_ DNA induced by Zα, Alexidine, and their binding affinity**

(A, B) Equilibrium end-point CD spectra of (CG)_6_ DNA titrated with increasing molar equivalents of Zα (A), or Alexidine (B). (C, D) Time-course CD measurements at a fixed wavelength of 255 nm, monitoring the B-to-Z transition of (CG)_6_ DNA in the presence of increasing concentrations of Zα (C), or Alexidine (D). (E, F) Overlay of CD spectra (E) and corresponding 255-nm time courses (F) comparing the maximal molar excess (10× over DNA) of each Z-inducer. (G) Normalized fraction of Z-DNA, derived from equilibrium end-point data, plotted as a function of the ratio of Z-inducer (Zα, or Alexidine) concentration to DNA; apparent K_D_ values are 1.05 µM (R^2^ = 0.91) for Zα, 8.78 µM (R^2^ = 0.85) for Alexidine.

**
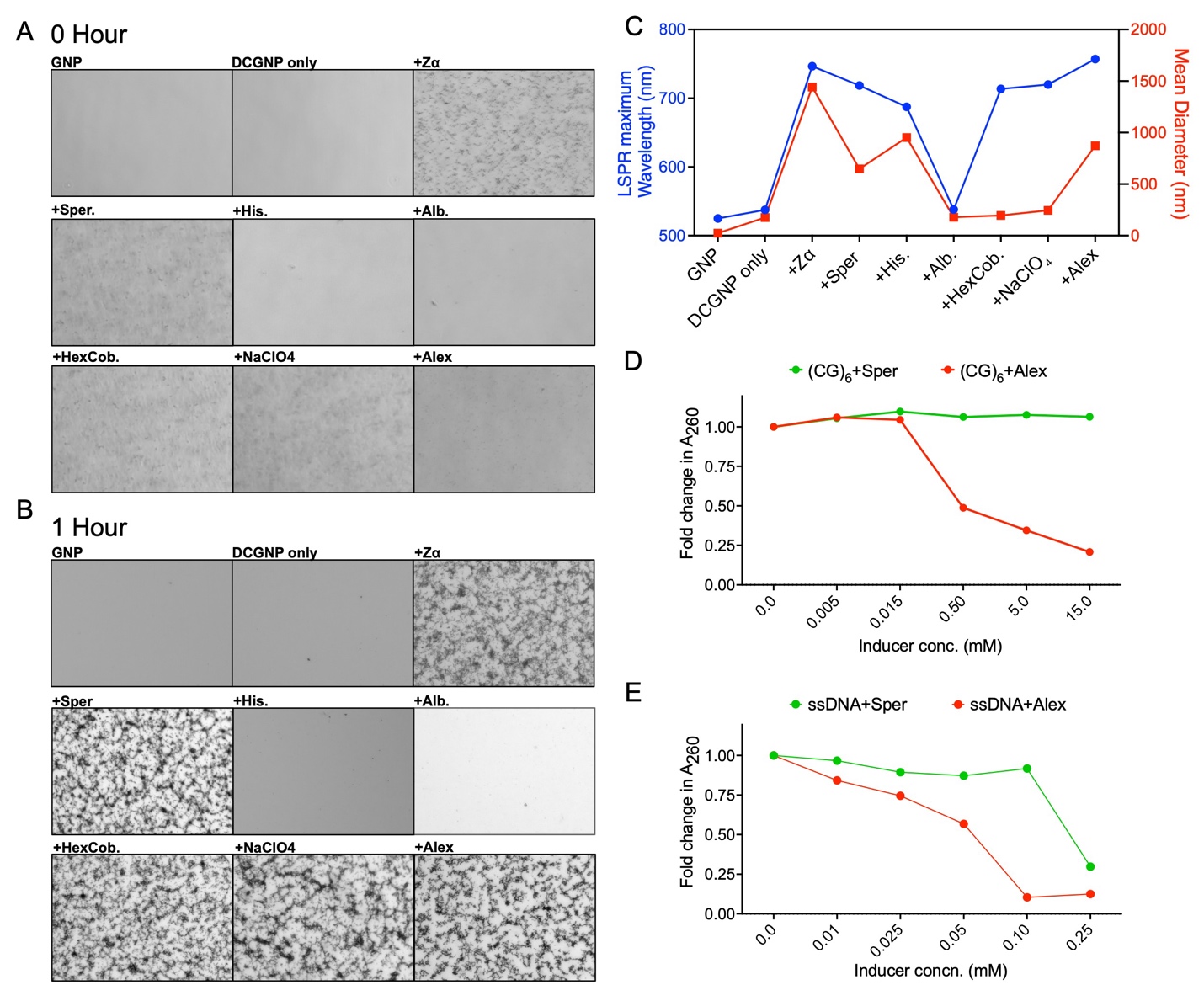
**

**Figure S8.** **Microscopic observation of DCGNP agglomeration and DNA condensation assay. (A-B)** Microscopic images of NanoZ DCGNP after adding the indicated Z-DNA inducers. Images were taken immediately after adding the inducers to DCGNP (**A**, 0 Hour) and after incubating for 1 hour at room temperature (**B**, 1 Hour). **(C)** Analysis of LSPR maximum and particle size in the reaction solution. The LSPR maximum was measured using UV-Vis spectroscopy, followed by dynamic light scattering (DLS) measurements to determine the size of particles. The line graph shows the LSPR maximum (blue) and mean hydrodynamic diameter (red). Pearson correlation analysis indicated a correlation coefficient (r) of 0.67 between the two measurements. **(D)** DNA condensation assay using 5 µg (= 15 µM) of (CG)₆ in the presence of varying molar concentrations of spermine and Alexidine. **(E)** DNA condensation assay in the presence of spermine and Alexidine using 5 µg of salmon sperm DNA as the substrate. Experimental procedures and their interpretation can be found in the Methods section (DNA condensation assay). Labels: Sper, spermine; Alex, Alexidine; HexCob, hexammine cobalt chloride; and ssDNA, salmon sperm DNA. The experimental detail and interpretation can be found in Methods as a title under “DNA Condensation assay”

**Figure S9. Motif enrichment of Z22-enriched regions from Alexidine-treated MEFs.**

The top 10 novel, ungapped motifs (recurring, fixed-length patterns) found either zero or once in the Z22 pulldown ChIP-seq peaks of Alexidine-treated MEFs predicted by MEME-suite. The motifs are ranked based on the E-value, which is a statistical estimate based on the log likelihood ratio, width, and number of sites of the motif in the sequences. The minimum and maximum motif widths were 5 and 100, respectively.


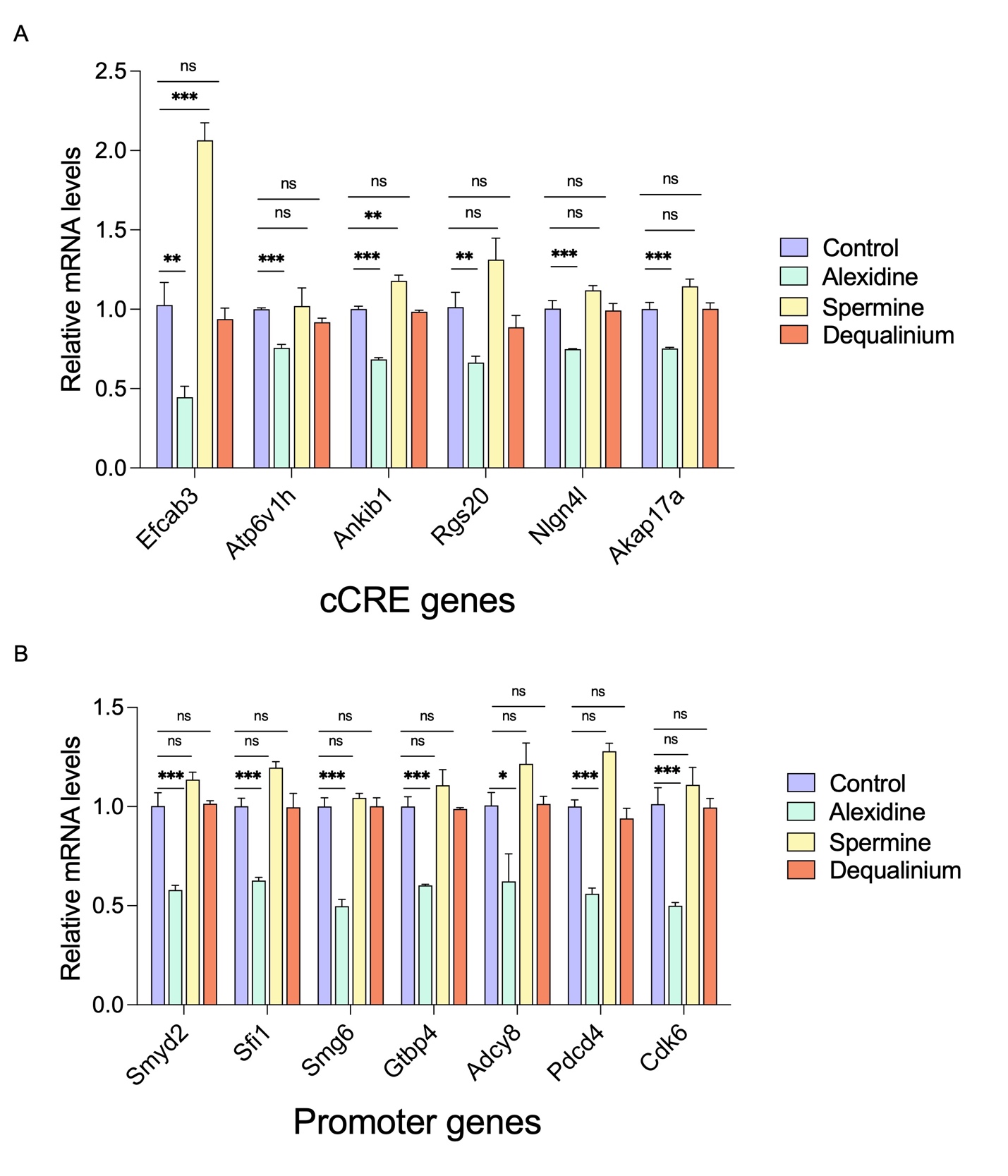


**Figure S10. Effect of Z-DNA/B-DNA binding small molecules on gene expression.**

RT-qPCR analysis of representative genes whose genomic regions overlapped with Z22-enriched (A) cCREs or (B) promoters in Alexidine, spermine, and Dequalinium-treated MEFs (n = 3).

**
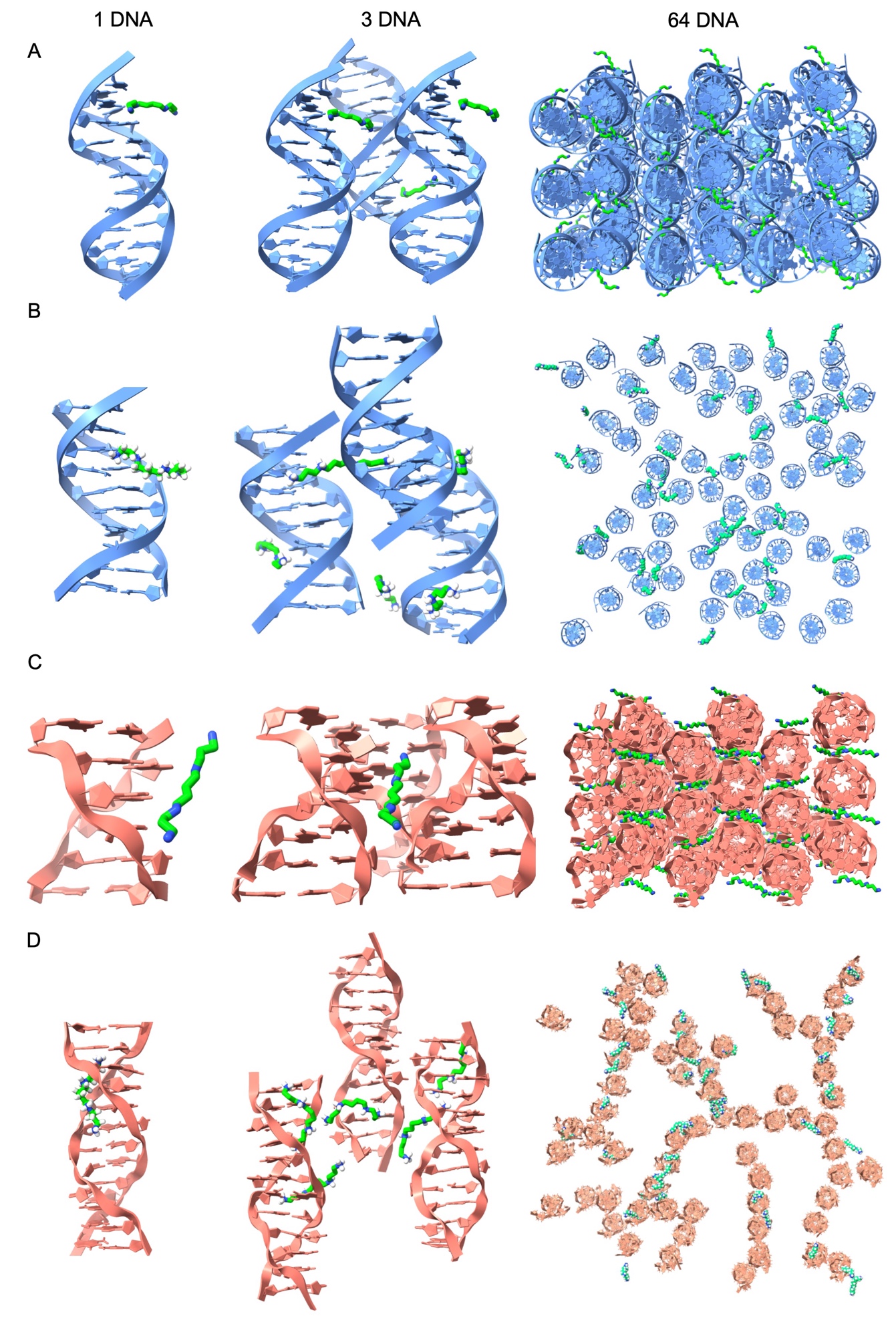
**

**Figure S11. Binding mode of spermine to DNA in the crystal structure and models generated from the MD simulations.**

Binding mode of (A) spermine to B-DNA (12 bp) in the crystal structure (PDB ID: 1EDR), (B) spermine to B-DNA (10 bp) as observed in the final MD snapshot at 50 ns in this study, (C) spermine to Z-DNA (6 bp) in the crystal structure (PDB ID: 1D48), (D) spermine to Z-DNA (12 bp) as observed in the final MD snapshot at 50 ns in this study. Binding mode of spermine to one DNA helix (left column), three DNA helices (middle column), 64 DNA helices (right column). DNA is shown as **ribbon models (**B-DNA – blue, Z-DNA – pink), and spermine molecules are shown in green stick models, colored by heteroatom.


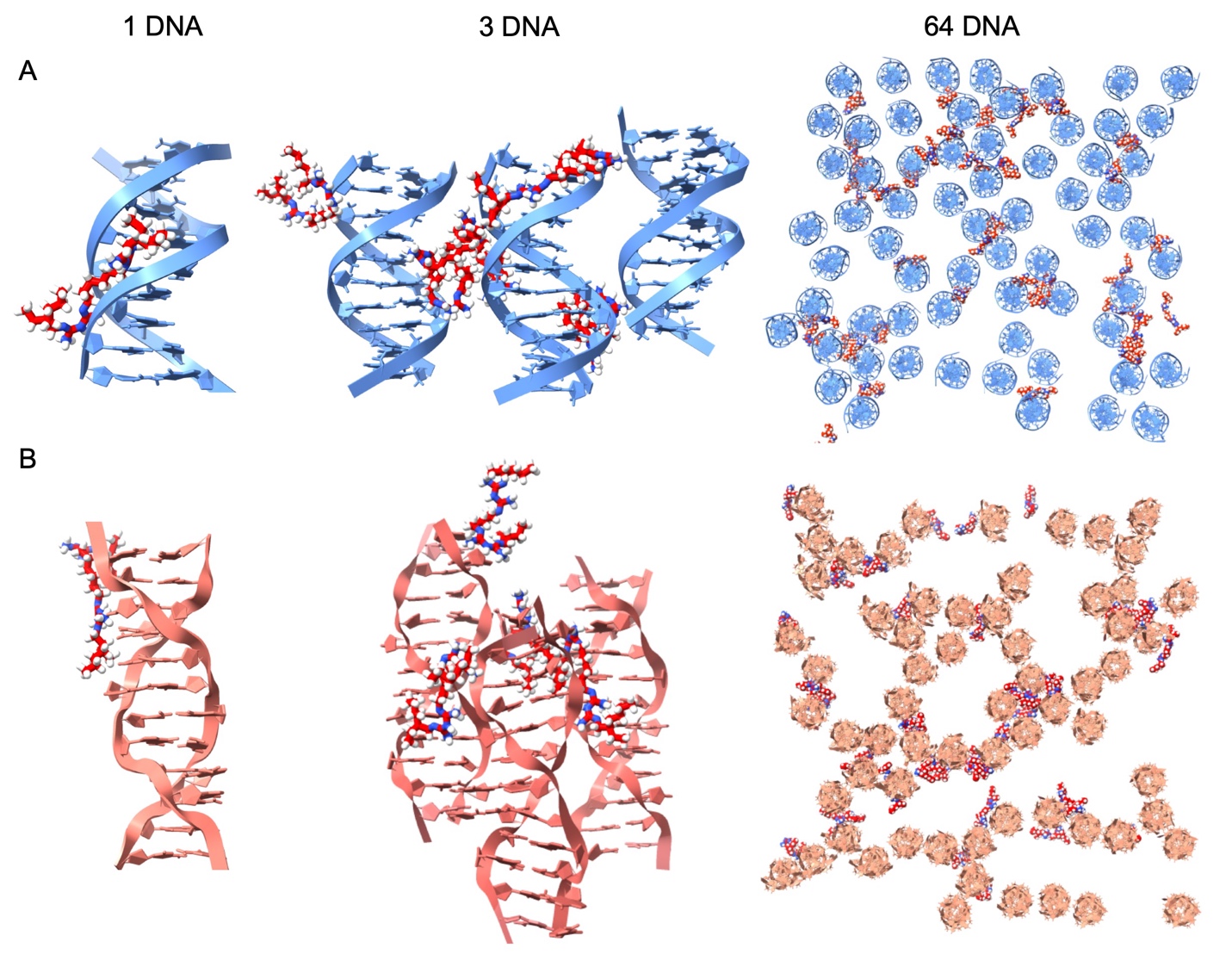
**Figure S12. The proposed binding modes of Alexidine to B-DNA and Z-DNA, based on models generated from the MD simulations.**

Binding mode of Alexidine to (A) B-DNA (10 bp) and (B) Z-DNA (12 bp) as observed in the final MD snapshot at 50 ns. Binding mode of spermine to one DNA helix (left column), three DNA helices (middle column), 64 DNA helices (right column). DNA is shown as **ribbon models (**B-DNA – blue, Z-DNA – pink), and Alexidine molecules are shown in red stick models, colored by heteroatom.


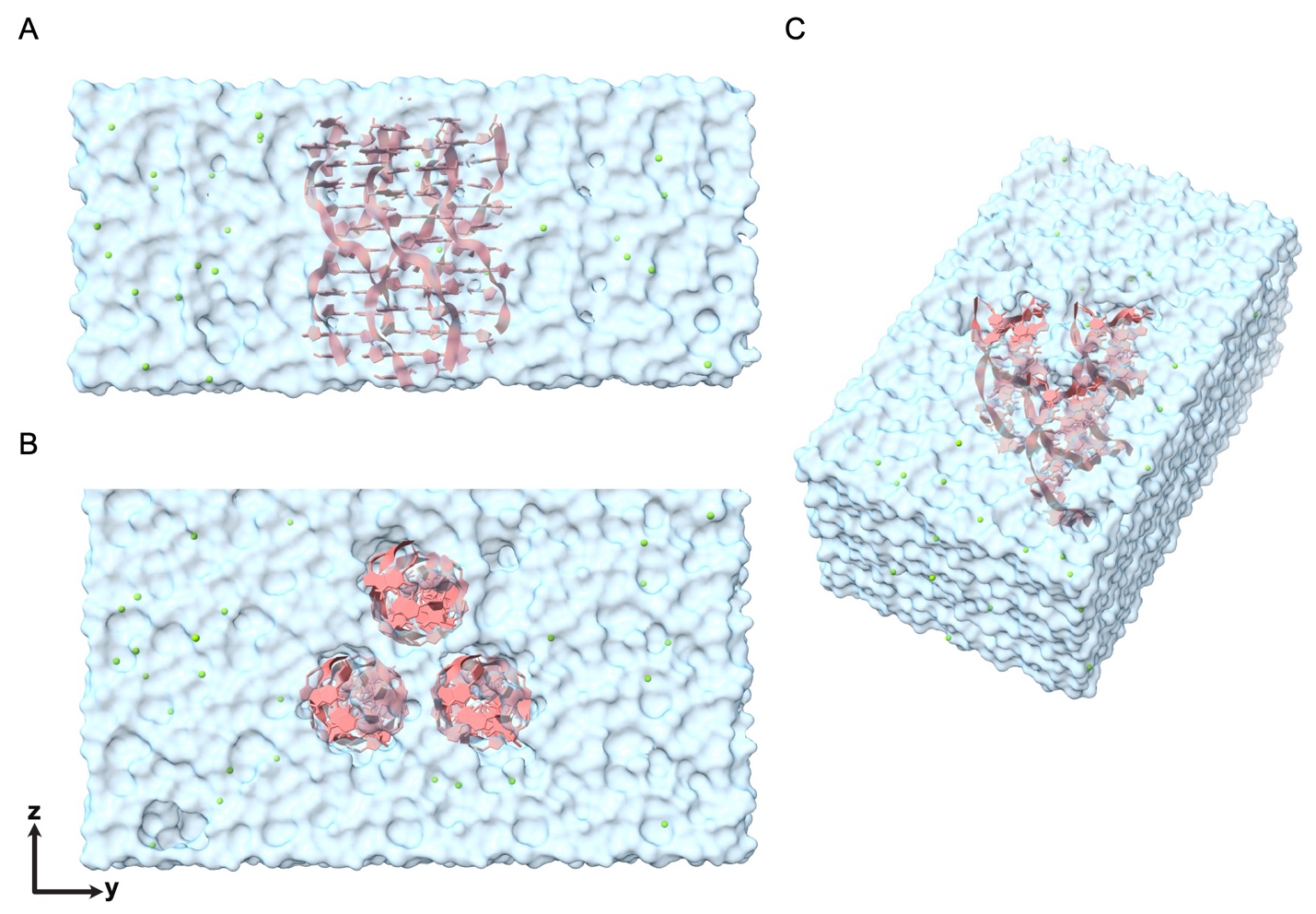


**Figure S13. MD experimental setup for simulating three Z-DNAs in the simulation box.**

(A) **Side view,** (B) **top view,** and (C) **diagonal view** of the system containing three identical Z-DNA double helices of (CG)_12_. The helices were enclosed within a cubic water box with dimensions 4.5 × 10.0 × 6.0 nm³, with the height of the box equal to the pitch of the DNA. The green spheres represent Mg²⁺ counter ions used for charge neutralization of the system.


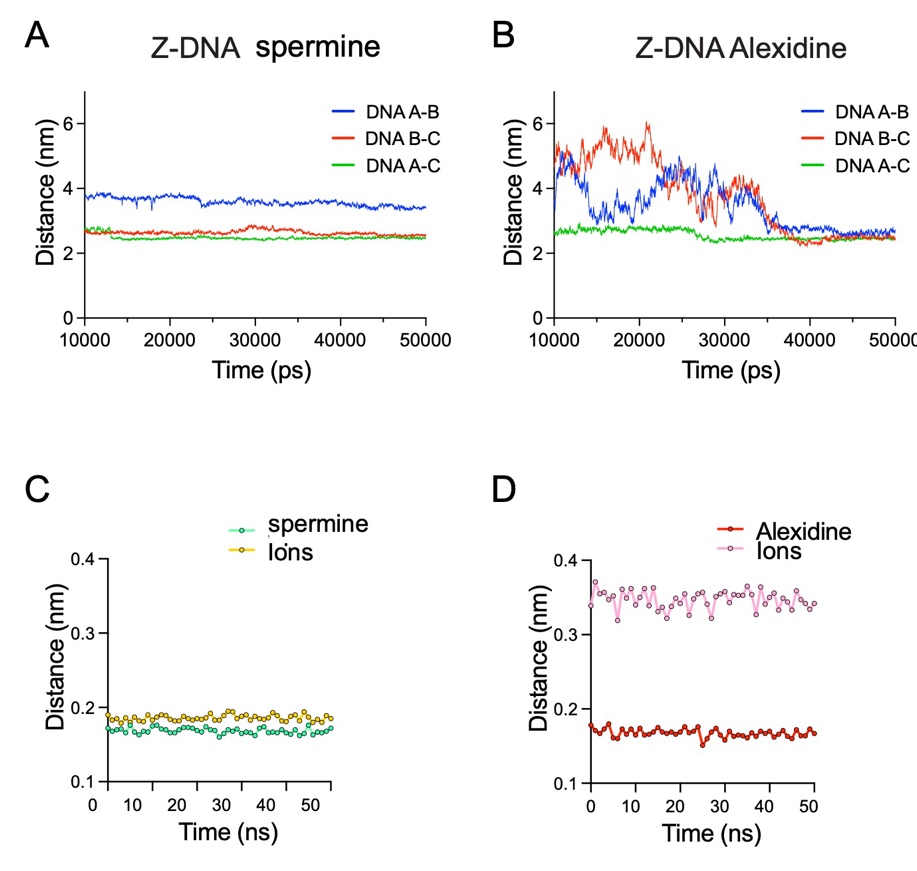


**Figure S14. Analysis of inter-Z-DNA duplex distance and Z-DNA-ions/ligand distances**.

(A, B) Inter-Z-DNA duplex distances amongst the three Z-DNA duplexes in the presence of spermine (A) and Alexidine (B) as a function of the simulation time. (C, D) The average distance between the ions and spermine (C) and Alexidine (D) from the center of mass of Z-DNA.


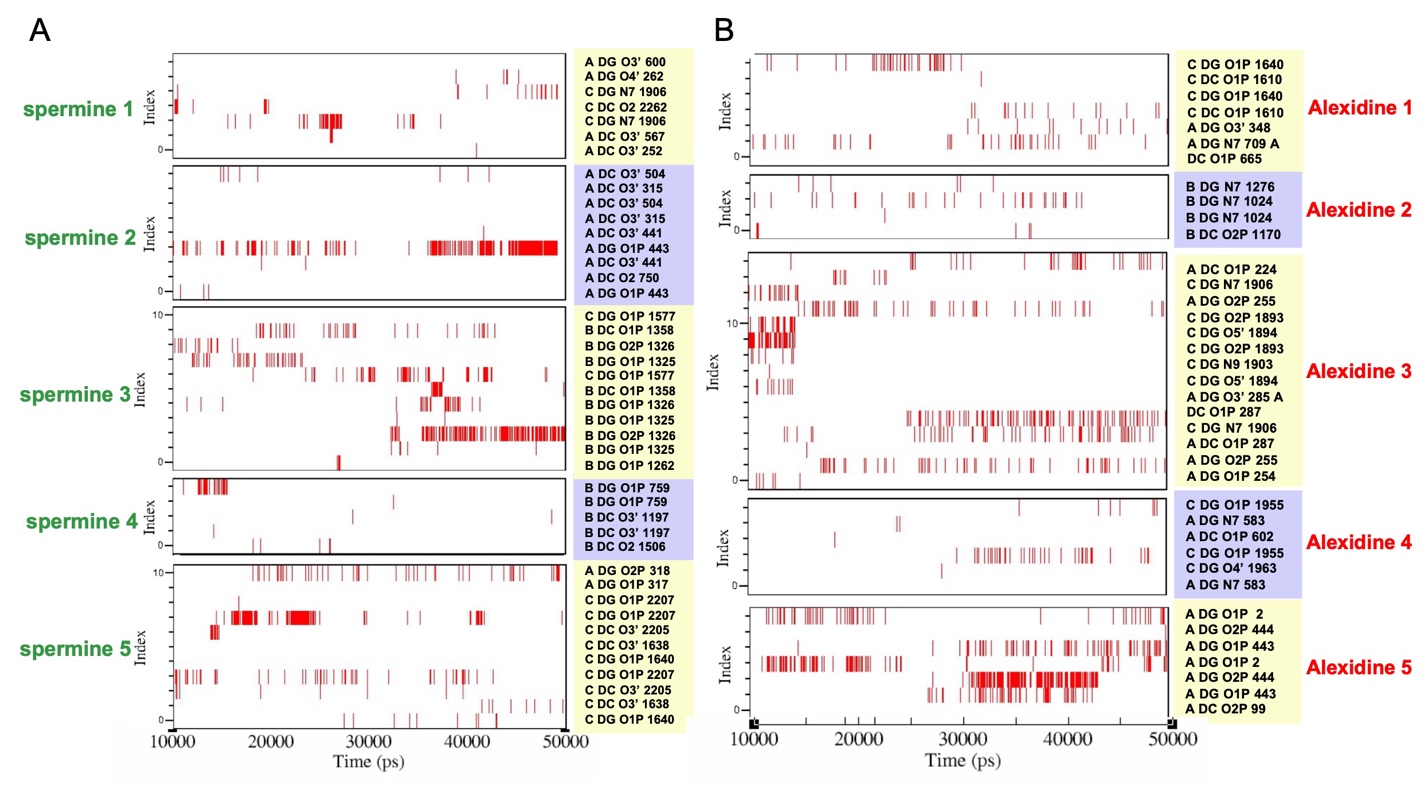


**Figure S15.** **Evaluation of the hydrogen-bond density map obtained from the MD simulations of three DNA duplexes.**

The density map of hydrogen bonds formed between the three Z-DNA duplexes and spermine (A) or Alexidine (B) during the final 40 ns of the MD simulation. The y-axis represents the nucleobase names and the respective atoms in the Z-DNA that are directly involved in forming the hydrogen bonds with the ligands.

**
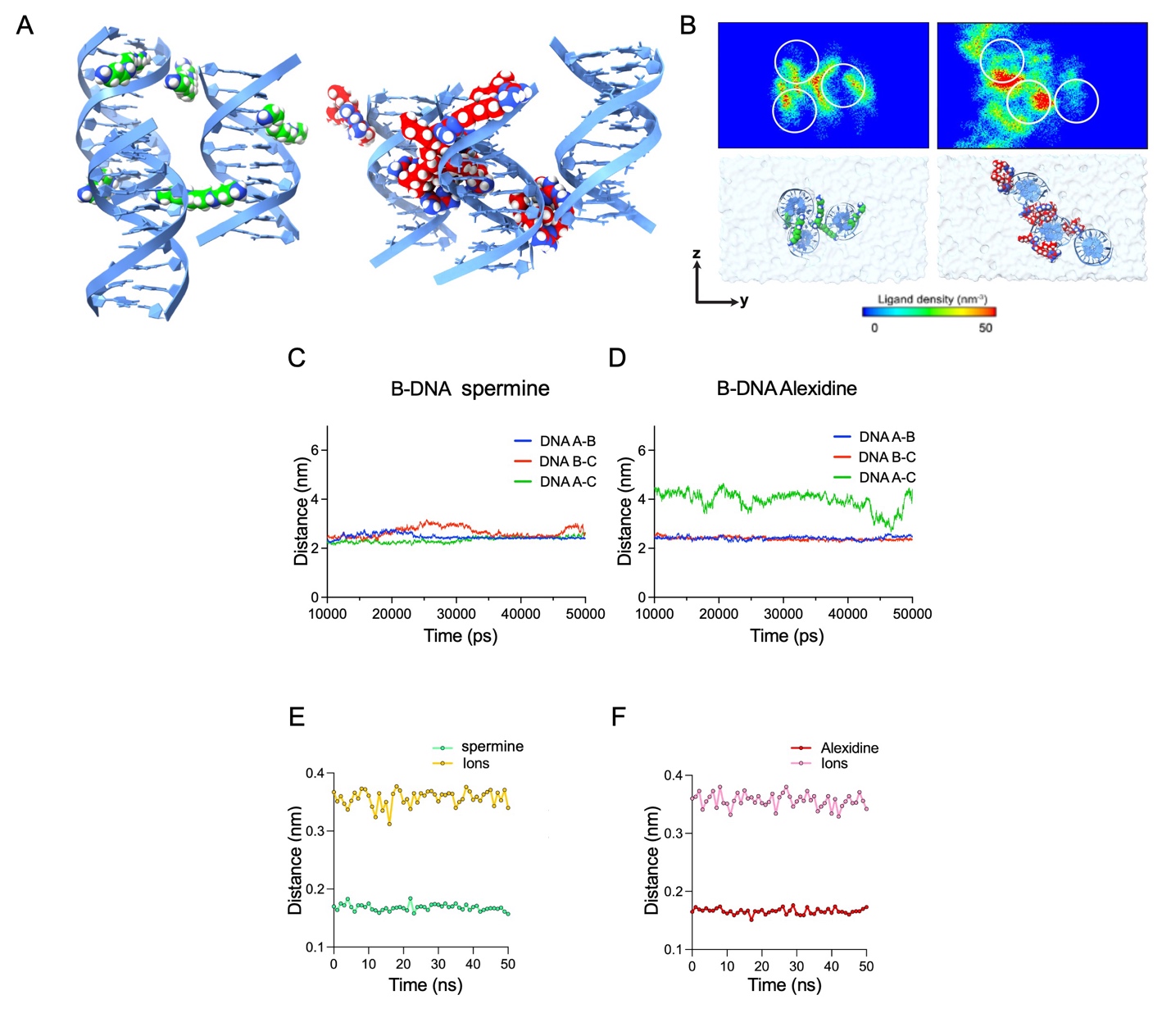
**

**Figure S16. Analysis of side-by-side interaction of B-DNA in the presence of Alexidine and spermine.**

(A) Final MD snapshot at 50 ns showing ligands in the presence of three duplexes of B-DNA, from left to right, shows models of spermine/B-DNA and Alexidine/B-DNA. (B) **Top:** Ligand density map of spermine (left) and Alexidine (right) projected along the B-DNA helical axes (x-axis), with DNA represented as white circles. **Bottom:** Representative simulation models of the three B-DNA duplexes system in an explicit water box, oriented to match the density maps above. **(C, D)** Inter-B-DNA duplex distances amongst the three B-DNA duplexes in the presence of spermine (C) and Alexidine (D) as a function of the simulation time. (E, F) The average distance between the ions and spermine (E) and Alexidine (F) from the center of mass of B-DNA. DNA is shown as **ribbon models**, and ligands are shown as space-filling models (carbon atoms in green for spermine and red for Alexidine).

**
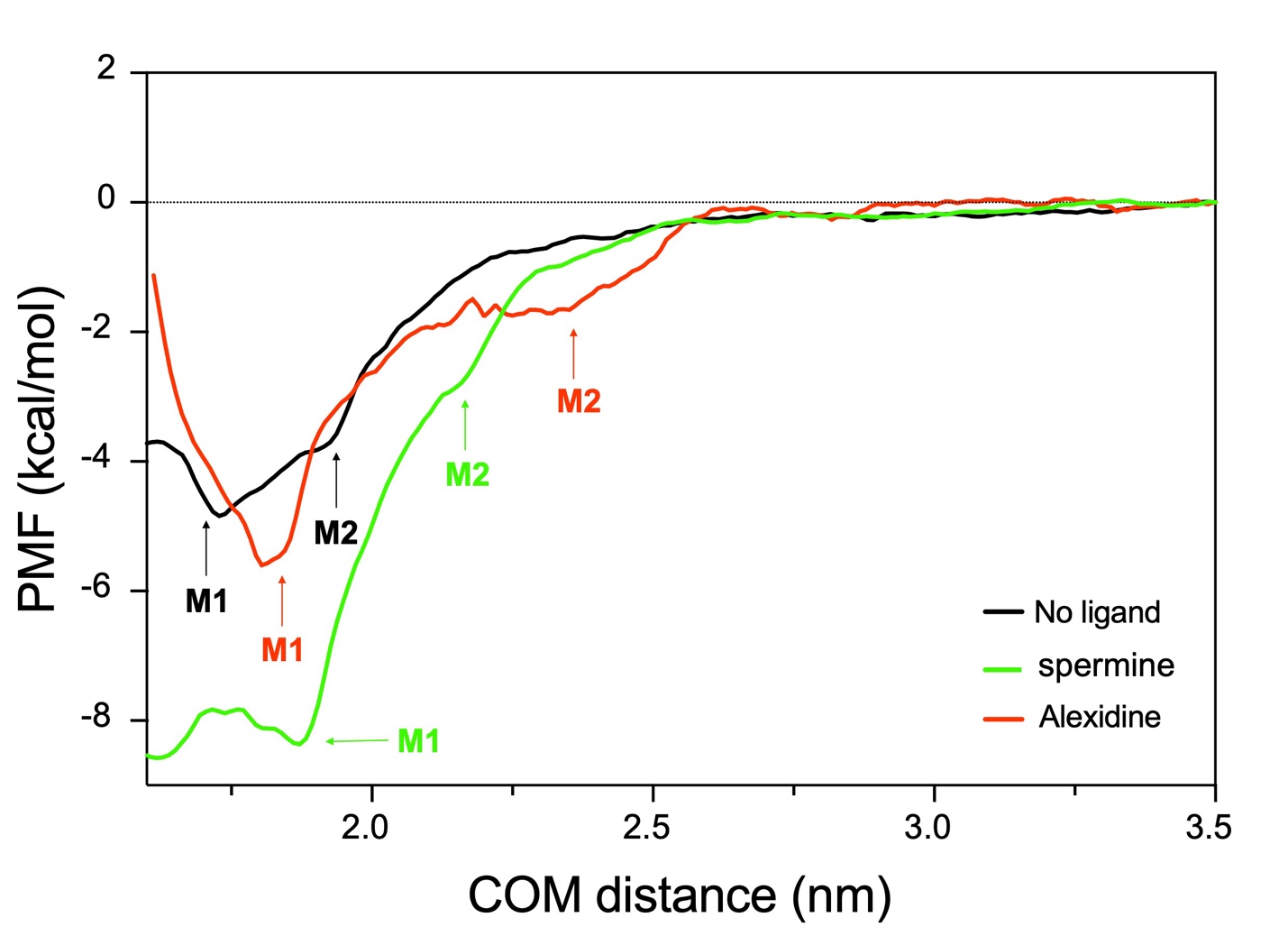
**

**Figure S17. MD simulation: potential of mean force (PMF).**

The PMF profile with respect to the inter-Z-DNA distance, both in the absence and presence of ligands. Umbrella sampling was performed at varying inter-Z-DNA distances ranging from 1.6 nm to 3.5 nm under different conditions. The arrows indicate the M1 and M2 states for each of the PMF profiles.


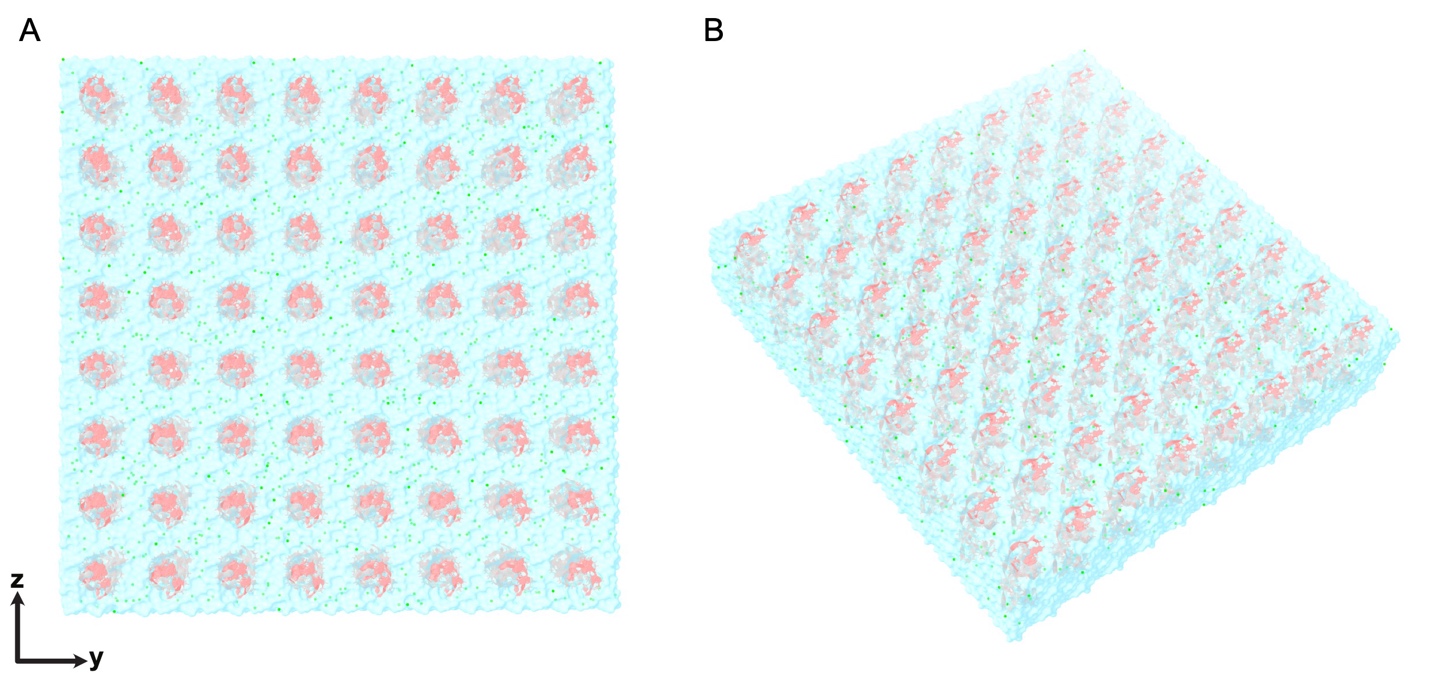


**Figure S18. MD simulation: 64 Z-DNA model system.**

(A) Top view and (B) diagonal view of the model system, with sixty-four identical Z-DNA double helices (12 bp), each consisting of (CG)_6_. The helices are enclosed within a cubic water box with dimensions 4.5 × 22.4 × 22.4 nm³.


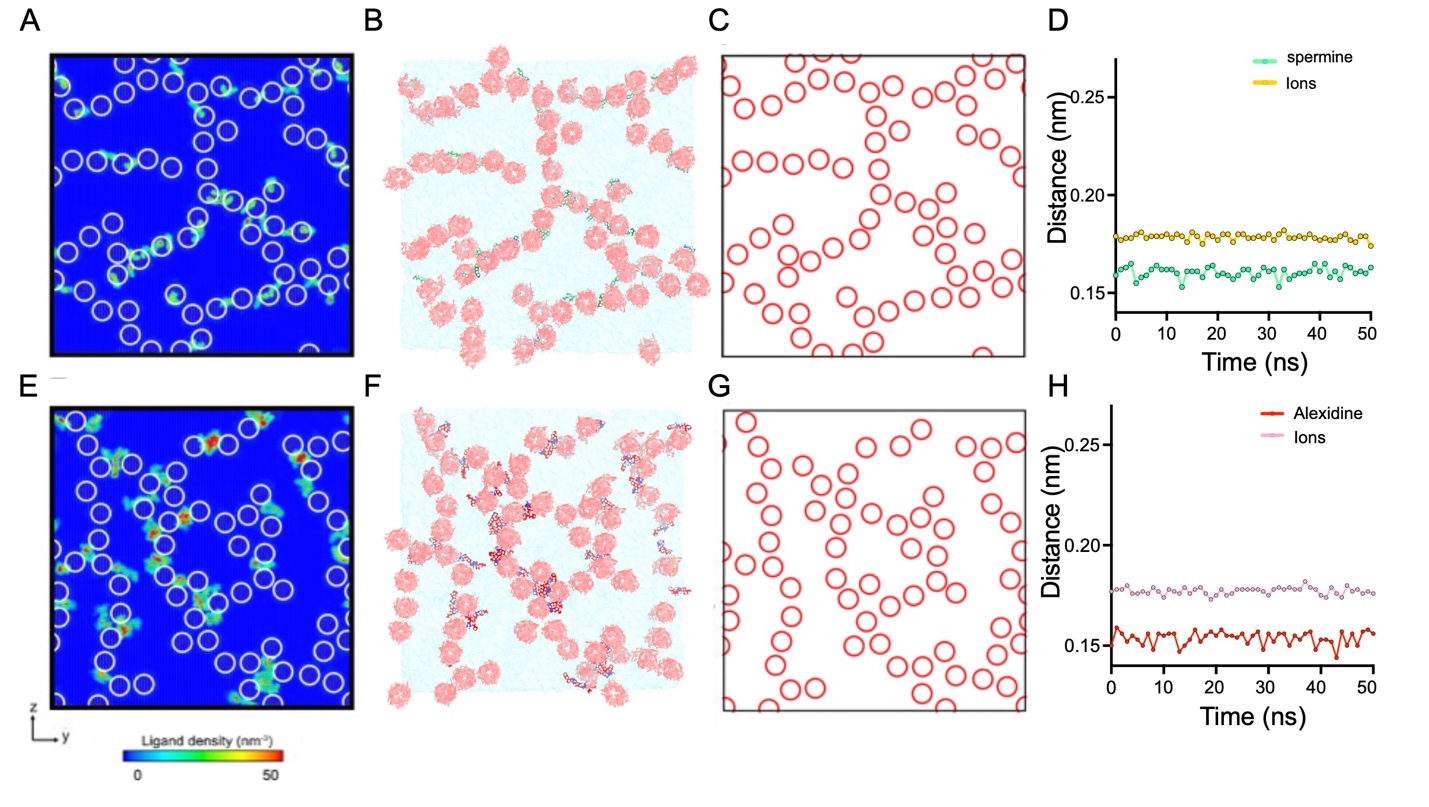


**Figure S19.** **MD simulation: low ligand density.**

50 ns MD simulation was performed for sixty-four copies of Z-DNA in the presence of low ligand density (40 molecules) of spermine (A-D) and Alexidine (E-H), respectively. The ligand density from a top view of the final snapshot after 50 ns of the MD simulation, where the white circle represents the width of the DNA double helix (A, E). Atomic models from the same snapshot are shown within the water box (B, F), where only the DNA is depicted as red open circles inside the water box (C, G). The average distance from the center of mass of the duplex DNA to the ligand or ions across the entire MD simulation duration (D and H).

**
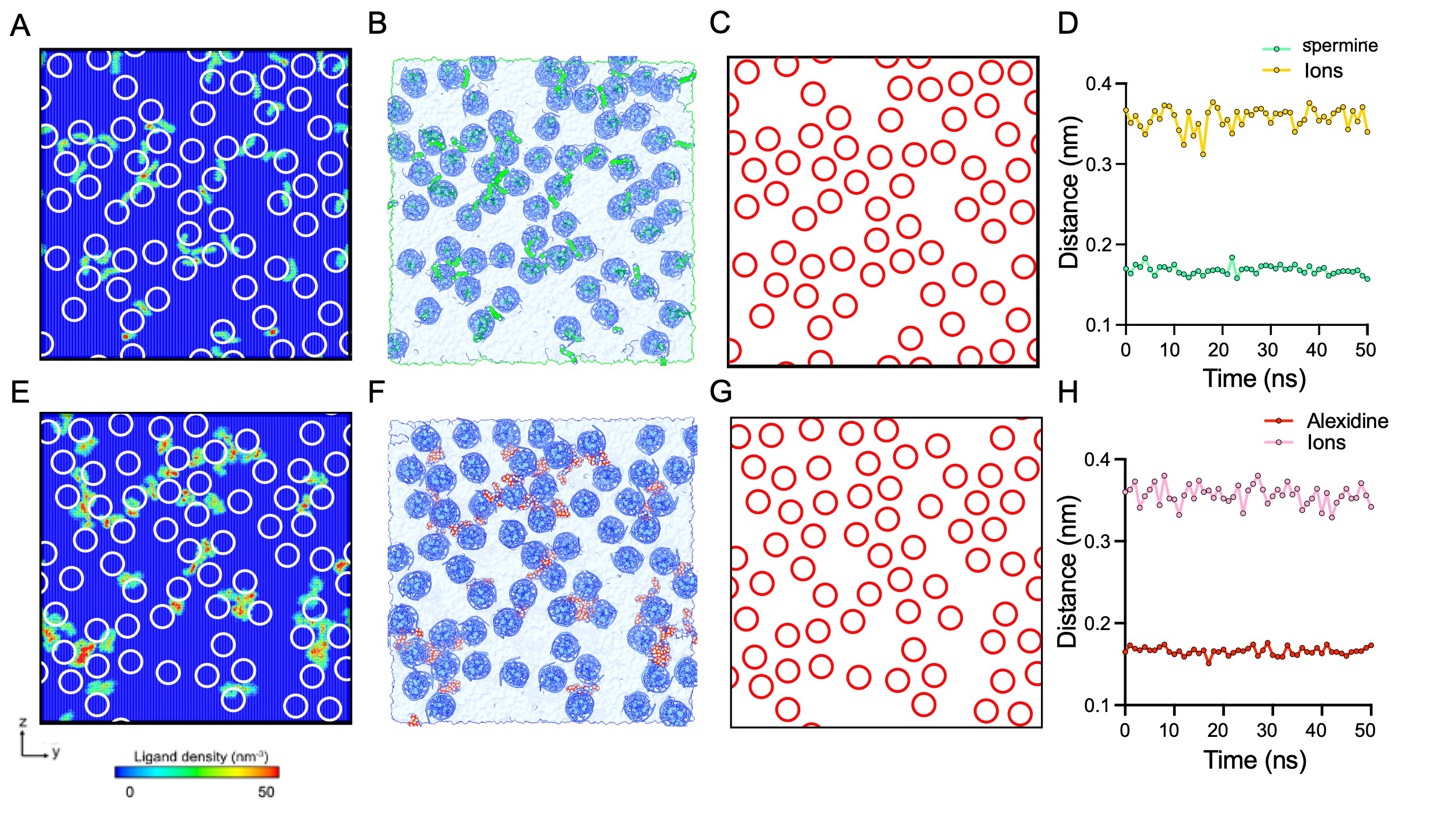
**

**Figure S20. MD simulation: B-DNA bundles.**

50 ns MD simulations were performed for sixty-four copies of B-DNA in the presence of 40 spermine (A-D) and Alexidine (E-H), respectively. The ligand density from a top view of the final snapshot after 50 ns of the MD simulation, where the white circle represents the width of the DNA double helix (A, E). Atomic models from the same snapshot are shown within the water box (B, F), where only the DNA is depicted as red open circles inside the water box (C, G). The average distance from the center of mass of the duplex DNA to the ligand across the entire MD simulation duration (D and H).


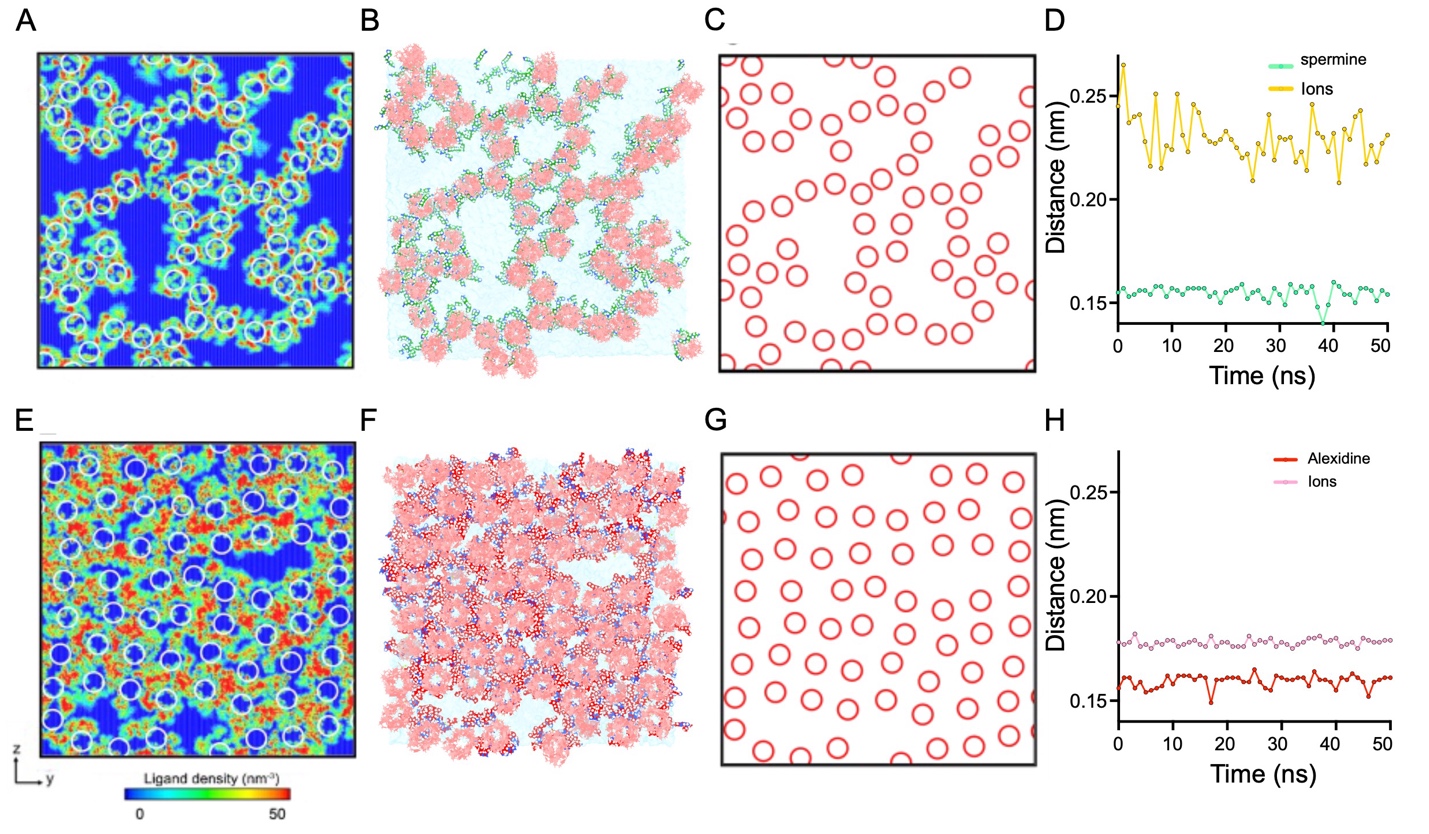


**Figure S21. Molecular dynamics simulation: maximum ligand density.**

50 ns MD simulations were performed for sixty-four copies of Z-DNA in the presence of maximum allowed ligands: 500 spermine molecules (A-D) and 322 Alexidine molecules (E-H), respectively. The ligand density from a top view of the final snapshot after 50 ns of the MD simulation, where the white circle represents the width of the DNA double helix (A, E). Atomic models from the same snapshot are shown within the water box (B, F), where only the DNA is depicted as red open circles inside the water box (C, G). The average distance from the center of mass of the duplex DNA to the ligand or ions across the entire MD simulation duration (D, H).

**
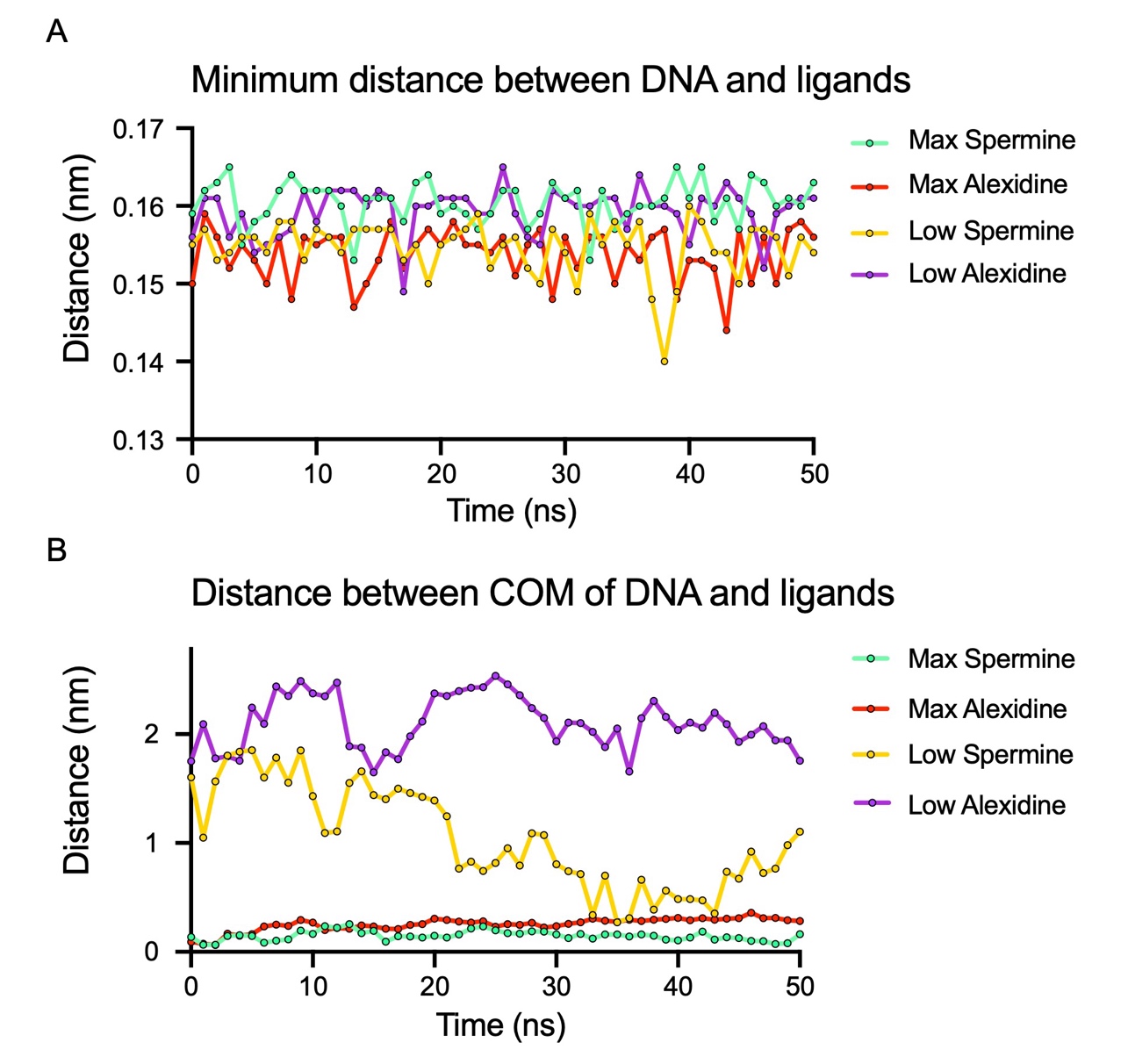
**

**Figure S22. MD simulation: distance between DNA and ligands.**

(A) The minimum distance between DNA and ligands under different ligand concentrations throughout the simulation time. (B) The average distance between the center of mass (COM) of duplex DNA and ligands throughout the simulation time. Labels: Max spermine - 500 spermines, Max Alexidine - 322 Alexidines, Low spermine or Alexidine - 40 molecules of the ligand were added in the simulation box randomly.**Movie S1:** **Timelapse clustering of DCGNP in the presence of Z⍺**
